# The Phylogeny of Rays and Skates (Chondrichthyes: Elasmobranchii) Based on Morphological Characters Revisited

**DOI:** 10.3390/d14060456

**Published:** 2022-06-06

**Authors:** Eduardo Villalobos-Segura, Giuseppe Marramà, Giorgio Carnevale, Kerin M. Claeson, Charlie J. Underwood, Gavin J. P. Naylor, Jürgen Kriwet

**Affiliations:** 1 Evolutionary Morphology Research Group, Department of Palaeontology, Faculty of Earth Sciences, Geography and Astronomy, University of Vienna, Josef-Holaubek-Platz 2,1090 Vienna, Austria; 2 Dipartimento di Scienze della Terra, Università degli Studi di Torino, Via Valperga Caluso 35, 10125 Torino, Italy; 3 Philadelphia College of Osteopathic Medicine, Philadelphia, PA 19103, USA; 4 School of Earth Sciences, Birkbeck College, Malet Street, London WC1E 7HX, UK; 5 Florida Museum of Natural History, University of Florida, 1659 Museum Road, Gainesville, FL 32611, USA

**Keywords:** character revision, phylogenetic analysis, morphology, Mesozoic, Cenozoic, fossils

## Abstract

Elasmobranchii are relatively well-studied. However, numerous phylogenetic uncertainties about their relationships remain. Here, we revisit the phylogenetic evidence based on a detailed morphological re-evaluation of all the major extant batomorph clades (skates and rays), including several holomorphic fossil taxa from the Palaeozoic, Mesozoic and Cenozoic, and an extensive outgroup sampling, which includes sharks, chimaeras and several other fossil chondrichthyans. The parsimony and maximum-likelihood analyses found more resolved but contrasting topologies, with the Bayesian inference tree neither supporting nor disfavouring any of them. Overall, the analyses result in similar clade compositions and topologies, with the Jurassic batomorphs forming the sister clade to all the other batomorphs, whilst all the Cretaceous batomorphs are nested within the remaining main clades. The disparate arrangements recovered under the different criteria suggest that a detailed study of Jurassic taxa is of utmost importance to present a more consistent topology in the deeper nodes, as issues continue to be present when analysing those clades previously recognized only by molecular analyses (e.g., Rhinopristiformes and Torpediniformes). The consistent placement of fossil taxa within specific groups by the different phylogenetic criteria is promising and indicates that the inclusion of more fossil taxa in the present matrix will likely not cause loss of resolution, therefore suggesting that a strong phylogenetic signal can be recovered from fossil taxa.

## 1 Introduction

Batomorpha (hereafter used to refer to a level above superorder level equivalent to Selachimorpha) is the largest subgroup of the Elasmobranchii *sensu* [1]; presently, they comprise 26 families and approximately 633 valid species [2]. Batomorphs differ from other elasmobranchs in having their five gill slits (six in one species) located on the ventral surface of the head; presenting a mostly dorsoventrally flattened body, with their eyes situated on the dorsal surface and their pectoral fins fused with the head and trunk forming a disc; and a lack of an anal fin [3].

Throughout their evolutionary history, batomorphs have successfully colonized various niches. Currently, most batomorph species are either benthic (living on the substrate) or benthopelagic (swimming close to the bottom but not resting on the substrate), with some members of the group being active pelagic swimmers and others living on the deep continental slope [3].

Systematically, batomorphs are a relatively well-studied group. Currently, they are considered monophyletic and are placed within the Elasmobranchii *sensu* [1,4], with several synapomorphies supporting this relationship [4–7]. However, several phylogenetic uncertainties surrounding them continue to pose problems. While some phylogenetic issues seem to be tackled with molecular data or addressed with detailed morphological descriptions, with their subsequent mapping on molecular phylogenies [8–10], the underlying fact remains that while these studies are of immense importance, they result in phylogenies without synapomorphies [11].

When dealing with more complex phylogenetic issues and in a wider evolutionary context, which requires the extensive inclusion of fossil taxa and the use of vast morphological data sets, the presence of phylogenies without synapomorphies becomes a significant problem for elasmobranch systematics, as it hinders the addition of fossil taxa. It is encouraging that in the last 40 years there has been a remarkable resurgence of interest in the reconstruction of the interrelationships of living sharks and rays from a paleontological perspective (e.g., [12–34]). However, many phylogenetic issues, such as the position of batomorphs within the Neoselachii *sensu* [7] (i.e., batomorphs as an offshoot of a branch of selachimorphs [35–38] or whether both modern selachimorphs and batomorphs are sister taxa [39–42]) and the ever-changing batoid intrarelationships recovered by morphological (e.g., [32,33]) and molecular (e.g., [41,42]) analyses, remain ignored. With most morphological phylogenetic works not finding a mutual monophyletic relation between selachimorphs and batomorphs, nor recovering clades recognized by some molecular analyses (e.g., Rhinopristiformes (guitarfishes, sawfishes, wedgefishes, banjo rays, panrays), Torpediniformes (thornbacks and electric), etc.), the current generalized acceptance among palaeoichthyologists and ichthyologists for the molecular phylogenetic hypotheses remains untested and unstudied under morphological characters.

Following a growing body of evidence derived from extant and extinct batomorphs and chondrichthyans in general (e.g., [8–10,12–34]), the overarching goal of the present study is to evaluate our current knowledge and to provide new hypotheses about the phylogenetic relationships of batomorphs using morphological characters. Detailed interpretations of the characters are included here to serve as a reference for future morphological works and to subsequently facilitate the inclusion of elasmobranch fossils into phylogenetic analyses. Different analytical approaches are employed and discussed in the present study, providing a novel look at the phylogenetic relationships of batomorphs. The present analysis also explores the phylogenetic relationships of batomorphs with their closest relatives (i.e., sharks) by including several characters for the selachimorphs and additional outgroups (e.g., holocephalians, symmoriids and hybodonts, and also highlighting issues that require future study.

## 2 Materials and Methods

A morphological data matrix of 87 terminal taxa and 142 characters was assembled in Mesquite V3.61 [43] (see data matrix in electronic Supplemental Material), of which 42 taxa are fossil species with relatively complete remains from different periods (Palaeozoic, Mesozoic and Cenozoic). Within these terminals, †*Ischyrhiza* is the most incomplete taxon with only 10% of the morphological characters scored. Considering the importance of outgroup sampling [44,45], we included 20 outgroup taxa comprising a non-acanthodian stem chondrichthyan (†*Doliodus latispinosus*), symmoriids (†*Cobelodus aculeatus* and †*Ozarcus mapesae*), holocephalian chimaeroids (*Chimaera* and *Harriotta*) hybodonts (†*Hamiltonichthys*, †*Hybodus reticulatus* and †*Tribodus*), as well as extant members of Hexanchiformes: Chlamydoselachidae (*Chlamydoselachus*) and Hexanchidae (*Hexanchus*); Heterodontiformes: Heterodontidae (*Heterodontus*); Orectolobiformes: Ginglymostomatidae (*Ginglymostoma*) and Hemiscylliidae (*Hemiscyllium*); Pristiophoriformes: Pristiophoridae (*Pristiophorus*); Carcharhiniformes: Triakidae (*Mustelus*) and Scyliorhinidae (*Scyliorhinus*); Squaliformes: Squalidae (*Squalus*); Squatiniformes: Squatinidae (*Squatina*); as well as some extinct sharks (†*Protospinax annectans* and †*Pseudorhina alifera*). Only Lamniformes of the known shark orders are not included in the present analysis, mainly because of the lack of specimens available for study. The recently discovered fossil of *Aquilolamna milarcea* is also not included. While the specimen is an excellent piece of exhibition, little to no phylogenetic features useful for our analyses are present in the specimen (JK, per. obser.).

The ingroup selection aimed to include as much batomorph diversity as possible. However, the current matrix is lacking two rajiform families (Gurgesiellidae, Anacanthobatidae), of which there were no specimens available for a detailed revision. The matrix thus includes 67 batoid taxa, including Jurassic batoids (Spathobatidae: †*Spathobatis,* †*Belemnobatis,* †*Kimmerobatis,* as well as †*Asterodermus*); Myliobatiformes (Zanobatidae: †*Plesiozanobatus, Zanobatus* and Myliobatoidei: †*Asterotrygon*, †*Heliobatis, Hexatrygon, Urolophus, Urobatis, Urotrygon, Plesiobatis, Hypanus, Potamotrygon, Neotrygon, Gymnura, Myliobatis, Aetobatus, Rhinoptera, Mobula*, †*Promyliobatis,* †*Arechia,* †*Lessiniabatis,* †*Tethytrygon);* Rajiformes (Rajoidei: *Raja, Bathyraja,* †*Ostarriraja,* †*Cyclobatis* and Sclerorhynchoidei: †*Ptychotrygon,* †*Asflapristis,* †*Onchopristis,* †*Ischyrhiza,* †*Sclerorhynchus,* †*Libanopristis);* Rhinopristiformes (†*“Rhinobatos” maronita,* †“*R*.” *latus,* †“*R*.” *hakelensis,* †“*R*.” *whitfieldi,* †*Stahlraja,* †*Tlalocbatus, Pristis, Rhynchobatus, Rhina, Glaucostegus, Rhinobatos, Pseudobatos,* †*Eorhinobatos,* †*Pseudorhinobatos, Trygonorrhina, Zapteryx, Aptychotrema,* †*Iansan* and †*Rhombopterygia);* Torpediniformes (Platyrhinoidei: *Platyrhina,* †*Eoplatyrhina, Platyrhinoidis,* †*Tingitanius,* †*Tethybatis* and Torpedinoidei: †*Titanonarke, Torpedo, Hypnos, Narcine, Narke* and *Temera).*

The phylogenetic analyses were carried out in TNT V1.5 [46,47], PAUP V4 [48] and MrBayes V3.2.7a [49]. For the parsimony analysis, the traditional parsimony search settings of TNT V1.5 with the following search parameters were used: mult = tbr replic 1000 hold 10 (random sequence addition, using tree bisection and reconnection algorithm for branch permutations with 1000 iterations, holding ten trees for each iteration). Jackknife and Bremer analyses were used to estimate clade support; the “resample” and “jak” commands were used for the support analysis under a regular Jackknife analysis (i.e., with independent deletion) with 1000 replications estimating the absolute frequencies of the groups on the strict consensus tree, leaving the remaining parameters in the default settings. The Bremer support was estimated based on suboptimal topologies 50 steps larger than the ones found in the parsimony analysis, collapsing groups with support lower than one (see script in electronic Supplemental Material).

The maximum-likelihood (ML) analysis was performed in PAUP V4, employing the Mkv model (Markov K model for discrete morphological data with only variable characters). The tree search used the tree bisection and reconnection algorithm for branch permutations with 10,000 replications, assuming a continuous gamma distribution across characters, using a neighbor-joining tree as starting point (see script in electronic Supplemental Material) and assuming a time limit for search of 2 h.

The Bayesian analysis was performed in MrBayes V3.2.7a under a Mkv model with gamma-distributed rates. This analysis combined the results of two independent runs, each employing four chains, and the following search options were used: ngen = 190,000,000, samplefreq = 500,000, printfr = 10,000, diagnfreq = 500,000, nruns = 2, checkfreq = 500,000, nchain = 4, temp = 1, stopval = 0.01, stoprule = yes, nperts = 2, savebrlens = yes, with a burnin fraction (discarded trees) of 35% (see script in electronic Supplemental Material).

Institutional Abbreviations—**AMNH:** American Museum of Natural History, Manhattan, NY, USA; **ANSP:** Academy of Natural Sciences of Drexel University, Philadelphia, PA, USA; **BHN:** Musée d’Histoire Naturelle de Boulogne-sur-Mer, Boulogne-sur-Mer, France; **BRC:** Birkbeck Reference Collection, London, UK; **BSP:** Bayerische Staatssammlung für Paläontologie und Geologie, Munich, Germany; **CAS:** California Academy of Sciencies, San Francisco, CA, USA; **CNPE-IBUNAM:** Colección Nacional de Peces del Instituto de Biología, Universidad Nacional Autónoma de México, Mexico; **CSIRO:** Commonwealth Scientific and Industrial Research Organization, Canberra, Australia; **DAE:** D.A. Ebert field number; **FMNH:** Field Museum of Natural History, Chicago, IL, USA; **GMBL:** College of Charleston, Grice Marine Biological Laboratory, Charleston, SC, USA; **HUMZ:** Hokkaido University Laboratory of Marine Zoology, Hokkaido, Japan; **IGM:** Colección Nacional de Paleontología del Instituto de Geologia, Universidad Nacional Autónoma de México, Mexico; **IPUW:** Palaeontological Collections of the University of Vienna, Wien, Austria; **JMSOS:** Jura Museum Eichtätt, Germany; **LACM:** Los Angeles County Museum of Natural History, Los Angeles, CA, USA; **MCZ:** Museum for Comparative Zoology, Cambridge, MA, USA; **MNHN** Muséum National d’Histoire Naturelle, Paris, France; **MSM:** Marine Science Museum, Tokai University, Tokyo, Japan; **MZUSP:** Universidade de Sao Paulo, Museu de Zoologia, Sāo Paulo, Brazil; **NHMUK PV P:** Natural History Museum United Kingdom, London, UK, Palaeontology Vertebrates; **SAM:** South African Museum, Cape Town, South Africa; **SIO:** Scripps Institution of Oceanography, La Jolla, CA, USA; **SMNS:** Staatliches Museum für Naturkunde Stuttgart, Stuttgart, Germany; **UF:** University of Florida, Florida State Museum, Gainesville, FL, USA; **UREJ:** Universidade do Estado do Rio de Janeiro, Rio de Janeiro, Brazil; **USNM:** National Museum of Natural History, Washington, DC, USA; **ZMB:** Museum für Naturkunde zu Berlin, Berlin, Germany.

## 3 Results

### 3.1 Matrix Revision

An extensive character review from over 100 scientific publications was carried out in the present study, which resulted in 142 matrix characters. This matrix includes 42 characters that correspond to features of the neurocranium; 21 characters that are features of the jaws and dentition; 19 characters that refer to the branchial skeleton; 40 characters that are features of the paired fins, pectoral and pelvic girdles and claspers; and the remaining 16 characters include features of the axial skeleton, dermal denticles and unpaired fins.

For the complete character list, see Supplemental Material. What follows is an account of the character modifications carried out in the present study and character additions, for which we provide new information and illustrations to facilitate their interpretation, along with a description of the character reconstruction in parsimony and maximum-likelihood phylogenetic trees indicating for which clades these characters are of importance. The characters are grouped according to their anatomical position; however, their numeration follows that of the character matrix.

#### 3.1.1 External Morphological Structures

31. **Anterior nasal lobe–mouth:** (0) Fails to reach the mouth; (1) reaches the mouth. Modified from Aschliman et al. [7] (char. 11), separated in two characters (31 and 32). Coding of Hexatrygon follows Heemstra and Smith Text-Figures 3, 4 and 6 in [50].

**Figure 1:**
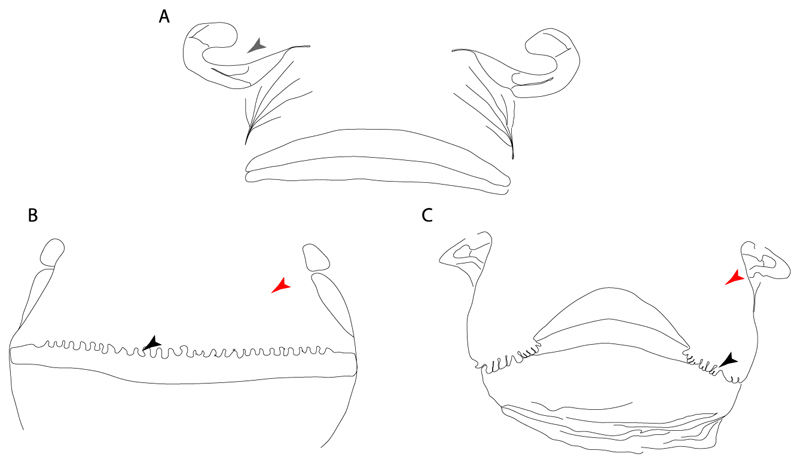
Interpretative drawings of the mouth and nostrils. State (0): **(A)** Zapteryx xyster modified from McEachran et al. Text-Figure 1B in [6]. State (1) **(B)** *Rhinoptera jayakari* modified from Pradeep et al. Text-Figure 3A in [51] and **(C)** *Raja clavata* modified from Steven Text-Figure 8 in [52]. Arrowheads: (red) nasal curtain, (back) nasal curtain fringes, (gray) anterior nasal lobe.

**Parsimony tree reconstruction (Ptr) and maximum-likelihood tree reconstruction (MLtr):** There is uncertainty regarding the plesiomorphic state for the tree, as there are several fossil taxa with an undetermined state. The revision of the extant material places an anterior nasal lobe not reaching the mouth (Figure 1A) as the plesiomorphic state for chondrichthyans. The presence of an anterior nasal lobe reaching the mouth (Figure 1B,C) is a shared feature of *Raja, Bathyraja, Torpedo, Narke, Temera, Trygonorrhina, Urolophus, Urobatis, Urotrygon, Plesiobatis, Hypanus, Potamotrygon, Neotrygon, Gymnura, Myliobatis, Aetobatus, Rhinoptera* and *Mobula.*

**MLtr (see discussion Maximum Likelihood tree):** A short anterior nasal lobe is the plesiomorphic state for Myliobatiformes, and within them there is a subsequent gain of the extension of the nasal lobe as a synapomorphy of clade 14.

32. **Anterior nasal lobe:** (0) Fails to cover most of the medial half of the naris; (1) covers more than the medial half of the naris.

**Figure 2:**
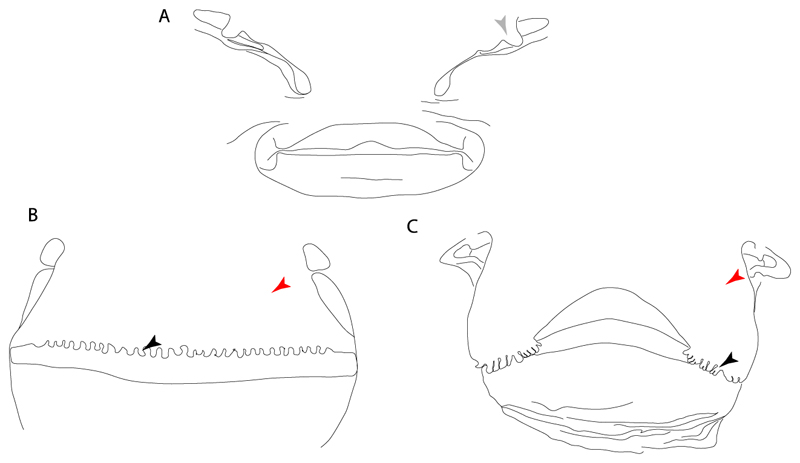
Interpretative drawings of the mouth and nostrils. State (0): **(A)** *Rhynchobatus immaculatus* redrawed from Last et al. Text-Figure 6 in [53]. State (1) **(B)** *Rhinoptera jayakari* redrawn from Pradeep et al. Text-Figure 3A in [51] and **(C)** *Raja clavata* redrawn and modified from Steven Text-Figure 8 in [52]. Arrowheads: (red) nasal curtain, (back) nasal curtain fringes, (gray) anterior nasal lobe.

**Ptr (see Parsimony tree):** An anterior nasal lobe covering more than the medial half of the naris (Figure 2B,C) is a synapomorphy of clade 26, with independent gains in *Zapteryx, Trygonorrhina, Rhina* and *Pristis.*

**MLtr (see discussion Maximum Likelihood tree):** An anterior nasal lobe covering more than the medial half of the naris is the plesiomorphic feature of clade 25, with independent gains in *Zapteryx, Trygonorrhina*, *Rhina, Pristis, Raja* and *Bathyraja.*

33. **Nasal curtain fringes:** (0) Absent; (1) present (new).

**Figure 3:**
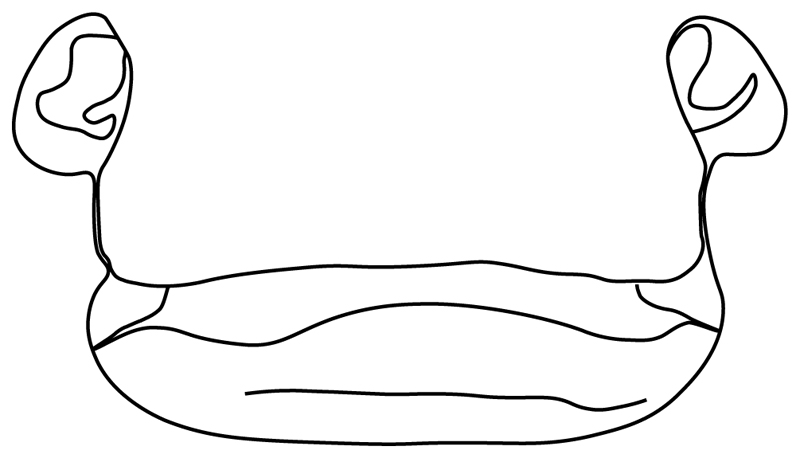
State (0): Interpretative drawings of dorsal mouth and nostrils of *Trygonorrhina fasciata* taken from (https://fishesofaustralia.net.au/home/genus/1555 (accessed on 10 February 2020)).

**Ptr and MLtr (see discussion Maximum Likelihood and Parsimony trees):** The use of reductive coding produces uncertainties in the reconstruction of the plesiomorphic state for this character. Fleshy fringes in the nasal curtain are present in *Raja, Bathyraja, Hypanus, Urobatis, Neotrygon, Urolophus Potamotrygon, Plesiobatis, Urotrygon, Myliobatis, Aetobatus,* *Mobula* and *Rhinoptera.* A nasal curtain without fringes is present in *Gymnura, Hexatrygon, Torpedo, Hypnos, Narcine, Narke, Temera* and *Trygonorrhina* (Figure 3). The wide distribution of these states among batomorphs possibly represents homoplasies.

#### 3.1.2 Lateral Line

83. **Abdominal canal on coracoid bar:** (0) Absent; (1) present. Modified from Aschliman et al. [7] (char. 24). The original character was split into two different ones (83–84) to increase the grouping information regarding the variation on the canals on the coracoid bar. The coding for *Pristis* was changed based on Wueringer et al. Text-Figure 1 in [54].

**Ptr and MLtr (see discussion Maximum Likelihood and Paroimony trees):** The lack of an abdominal canal on the coracoid bar is the plesiomorphic state for the chondrichthyan tree. Within batomorphs, the electric rays (clade 18;) and stingrays (clade 16) retain the plesiomorphic condition. The presence of a canal is widely distributed among batomorphs (Rajiformes: *Raja;* Torpediniformes: *Platyrhina, Platyrhinoidis;* Rhinopristiformes: *Rhynchobatus, Glaucostegus, Rhinobatos, Pseudobatos, Trygonorrhina, Zapteryx, Aptychotrema,* and Myliobatiformes: *Zanobatus*), and according to the present topology, this is considered a single gain between these groups.

**MLtr (see discussion Maximum Likelihood tree):** The loss of the canals is recovered as independent events and a synapomorphy of clades 18 and 16.

84. **Abdominal canal on coracoid bar (if present):** (0) Groove, cephalic lateral line forms abdominal canal on coracoid bar; (1) pores.

**Ptr (see discussion Parsimony tree):** The presence of pores in the canal is the plesiomorphic state for clade 27, being present in *Platyrhina, Platyrhinoidis* and *Zanobatus.* The presence of a groove is a shared feature for clade 19. However, there is uncertainty regarding the plesiomorphic state for Rhinopristiformes as *Trygonorrhina, Zapteryx* and *Aptychotrema* present pores.

**MLtr (see discussion Maximum Likelihood):** The presence of a groove is the plesiomorphic state for batomorphs. The presence of pores is recovered as shared feature for the taxa within clade 24, being present in *Trygonorrhina, Zapteryx, Aptychotrema, Platyrhina, Platyrhinoidis* and *Zanobatus* and inapplicable for the rest of myliobatiforms and electric rays.

#### 3.1.3 Neurocranium

**Figure 4:**
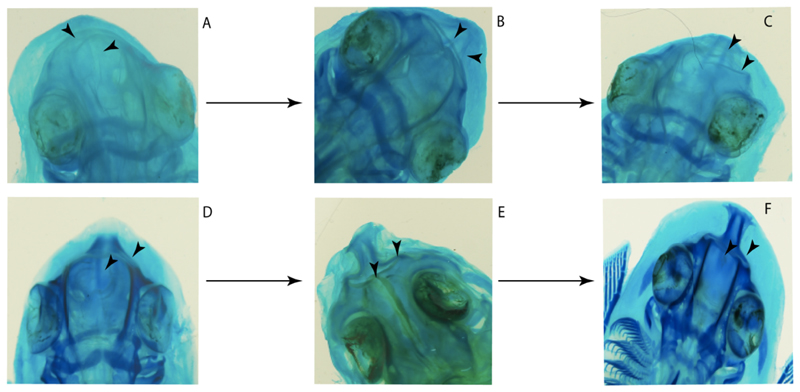
Generalization of the development of the rostral cartilages in Neoselachii using *Zapteryx brevirostris* (UREJ, unpublished data). **(A–F)** Letter sequences follow the order of the morphological development; arrowheads show the regions involved in the growth.

3. **Rostral cartilages:** (0) Arise from the medial area of the trabecula only; (1) medial area of the trabecula + lamina orbitonasalis. According to De Beer and Moy-Thomas [55] and Miyake et al. [56], no evidence suggests the homology between the rostral cartilages in elasmobranchs and holocephalians, as the rostral cartilages of holocephalians arise from the medial area of the trabecula possibly without any contribution from the lamina orbitonasalis. Conversely, in most modern elasmobranchs, these two embryological cartilages (medial trabecula and the lamina orbitonasalis) contribute to the formation of the rostral cartilages (Figure 4).

The coding for †*Cobelodus* and †*Ozarcus* follows Maisey [18] and Pradel et al. [22]. The reduced rostrum in these taxa seems to be a product of the outgrowth of the posterior portion of the ethmoidal region (possibly trabecula) with little to no contribution of the lamina orbitonasalis Text-Figures 2, 8,10,39 and 48A,B in [18]. The coding for hybodonts uses thee topological relations of the rostral bar and caudal internasal keel Text-Figure 2 in [57], Text-Figure 3a,b in [58]. In the fossil genera †*Ostarriraja* and †*Arechia*, due to the lack of preservation, we were unable to determine the state of this character (?) (see [30, 59]). However, being batomorphs, it is very likely that their rostral cartilages, although reduced, arose from the interaction between the medial area of the trabecula and the lamina orbitonasalis.

**Ptr and MLtr (see discussion Maximum Likelihood and Parsimony trees):** Rostral cartilages that topologically seem to have arisen for the medial area of the trabecula in interaction with the lamina orbitonasalis is a synapomorphy for the euselachian clade (Hybodontiformes + Elasmobranchii). The absence of these interactions in symmoriids and holocephalians is a common feature between these groups and the plesiomorphic feature for the chondrichthyan tree.

4. **Rostral cartilage:** (0) Well-developed rostral plate with various degrees of contribution from the lamina orbitonasalis; (1) reaches the tip of the snout (carried by the growth of the pectoral fin); (2) reaches the tip of the snout (growth of lamina orbitonasalis to support the cephalic fins). Modified from Aschliman et al. [7] (char. 26). This character aims to include the variation observed in Neoselachii (*sensu* [12]), following observations made by Miyake et al. [56], Maisey [57] and Lane [58]. Many taxa remain uncharacterized as the present coding provides grouping information for those taxa whose rostral cartilages arise from the interaction between the medial area of the trabecula and lamina orbitonasalis.

**Figure 5:**
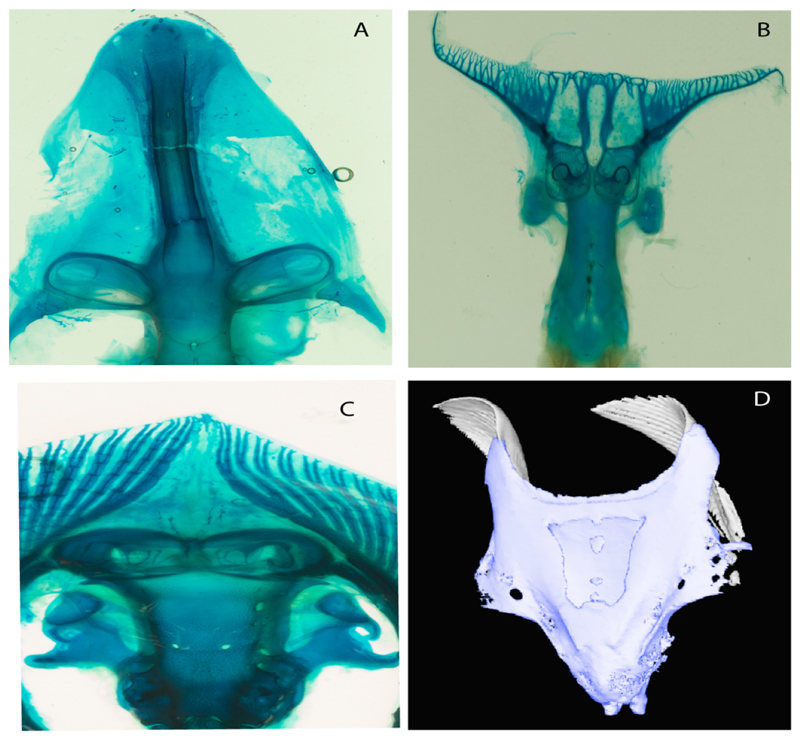
Variation on the development of the rostral cartilages among selected batoid taxa. **(A)** State (0): *Pseudobatos lentiginosus* (AMNH 8913), **(B)** *Torpedo ocellata* (AMNH 4128). State (1) **(C)** *Urotrygon venezuelae* (AMNH 55623). State (2) **(D)** *Mobula munkiana* (https://sharksrays.org/ (accessed on 15 March 2020)).

**Ptr and MLtr (see discussion Maximum Likelihood and Parsimony trees):** Presence of well-developed rostral cartilages (Figure 5B,D) is the plesiomorphic condition for euselachians. Within batomorphs, there is the appearance of two additional states: (1) rostral cartilages located between the tip of the pectoral fins that reach the tip of the snout (Figure5A), which are a shared feature among stingrays being present in *Gymnura, Potamotrygon, Urotrygon, Urolophus* and †*Asterotrygon;* (2) growth of lamina orbitonasalis to support the cephalic fins (Figure 5C), which is a synapomorphy foa the *Mobula* + *Rhinoptera* clade.

**MLtr (see discussion Maximum Likelihood tree):** The rostral cartilages located between the tip of the pectoral fins, reaching the tip of the snout (Figure 5A), are recovered as a synapomorphy of clade 29 with an additional gain in *Urotrygon.*

**Figure 6:**
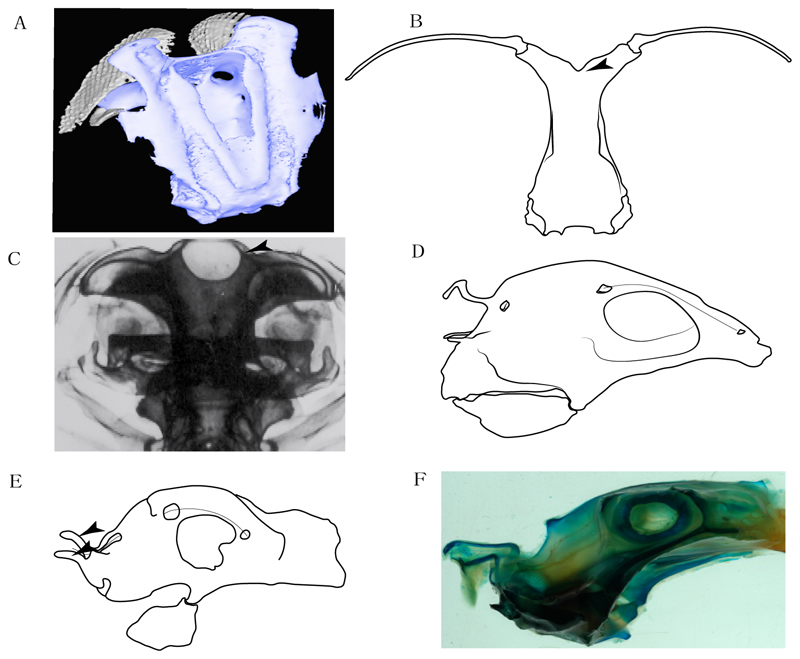
Variation on the development of rostral cartilages among selected chondrichthyan groups. State (0) **(A)** *Rhinoptera bonansus* (GMBL 73 https://sharksrays.org/ (accessed on 23 February 2020) and **(B)** *Hypnos monopterygius* (USNM 84374 https://sharksrays.org/ (accessed on 23 February 2020)). State (1) **(C)** *Zanobatus* sp. (MNHN 1989.12.91); Inapplicable (-) **(D)** *Chimaera cubana* (USNM 400700 https://sharksrays.org/ (accessed on 23 February 2020)) and **(E,F)** *Callorhinchus capensis* (AMNH 36943); Arrowheads indicate the lateral rostral process and medial rostral process in Holocephali, in *Zanobatus* the arrowhead the medial growth, and in *Hypnos* the medial region.

5. **Medial growth of rostral cartilage:** (0) Inconspicuous; (1) conspicuous (noticeable). Modified from Aschliman et al. [7] (char. 26), Villalobos-Segura et al. [32] (char. 27) and Claeson et al. [23] (char. 2). De Beer and Moy-Thomas [55] named the cranial projections observed in chimaeroids (Figure 6A–C) as “medial and lateral rostral processes”. This nomenclature is kept by Claeson [60] and used to recognize the structures on the rostral cartilages of electric rays. However, the topological homology of these structures is unclear. Because of this, we coded the medial growth of rostral cartilage in chimaeroids as inapplicable (-). The coding of this character for *Ginglymostoma* follows Motta and Wilga Text-Figures 8 and 9 in [61]. The character state is inconspicuous (0) for both *Torpedo* and *Hypnos* (Figure 6E,F); in *Hypnos,* the antorbital cartilages and nasal capsules support the anterior extension of the pectoral disc, and in *Torpedo,* the rostral cartilages seem to arise primary from the anterior wall of the nasal capsules and interact with the antorbital cartilages. In *Narke,* there are tripodal rostral cartilages with lateral and medial growths. *Narcine* also shows a noticeable development of its rostral cartilages. We consider the observations by Miyake [62] in *Urolophus, Urotrygon* and *Potamotrygon* to be correct, as these taxa present evident vestiges of the rostral cartilages, such as those in *Gymnura* Text-Figure 1 in [63], Text-Figure 1 in [64]. Aschliman et al.’s [7] coding for *Urobatis* was kept, as we were unable to confirm the observations of Miyake et al. [56] and McEachran et al. [6] on the presence of these vestiges. *Rhinoptera* and *Mobula* (Figure 6F) present a lateral growth on the trabecula and lamina orbitonasalis to support the cephalic fins [56]. *Zanobatus* and †*Plesiozanobatus* show a small medial growth of the rostrum (Figure 6D). De Carvalho et al. [65] noticed in specimens of †*Asterotrygon* (NHMUK P 61244; PF 15180) the rostral projections and medial growth. The rostral cartilages in †*Heliobatis, Plesiobatis, Hexatrygon, Hypanus, Potamotrygon, Neotrygon, Aetobatus, Myliobatis*, †*Lessiniabatis*, †*Tethytrygon* and †*Promyliobatis* are inconspicuous. The rostral cartilages in the most rajoids (not in *Sympterygia),* and in all sclerorhynchoids, extant and extinct platyrhinids and Rhinopristiformes, are noticeable.

**Ptr and MLtr (see discussion Maximum Likelihood and Parsimony trees):** †*Doliodus* presents medial growth of the rostral cartilages, identifying this as the plesiomorphic condition. However, as symmoriids and holocephalians are coded as inapplicable, there is uncertainty in this character-state reconstruction. There is a subsequent loss of growth in the clade comprising *Hypnos* and *Torpedo,* with additional losses in the Myliobatiformes clade (absent in *Hexatrygon, Plesiobatis*, †*Heliobatis*, †*Lessiniabatis, Urobatis*, †*Tethytrygon, Neotrygon, Hypanus, Promyliobatis, Myliobatis, Aetobatus, Mobula, Rhinoptera*).

**MLtr (see discussion Maximum Likelihood tree):** The lack of rostral-cartilage growth (Figure 6A) is an independent loss and a synapomorphy for [*Hypnos* + *Torpedo*] and clade 31.

6. **Different cartilage structures on rostrum (highly porous peripherical and fibrous at the central portion):** (0) Absent, (1) present (new).

**Figure 7:**
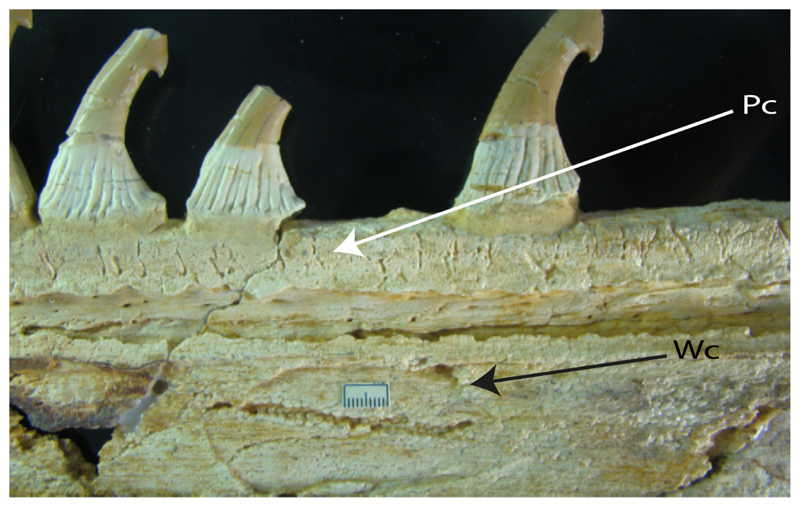
State (1): Rostrum section of: †*Onchopristis numidus* IPUW 353500. *Abbreviations:* Pc, peripheral cartilage; Wc, wood-like cartilage.

**Ptr and MLtr (see discussion Maximum Likelihood and Parsimony trees):** The presence of two noticeable “types” of cartilages in the rostrum is not exclusive of †*Onchopristis* and †*Ischyrhiza,* as different types of cartilage have been observed in Lamniformes [66]. However, the presence of a highly porous peripheral cartilage at the sides of the rostrum and fibrous wood-like cartilage at the central portion pattern across the rostrum is currently restricted to †*Onchopristis* and †*Ischyrhiza* (Figure 7).

7. **Rostral processes:** (0) Absent; (1) present. Modified from Aschliman et al. [7] (char. 29); Claeson et al. [23] (char. 12). The presence of hyaline and poorly calcified structures called “rostral processes”, are shared features of platyrhinids according to de Carvalho [67]. These structures are not considered homologous to the rostral appendices of Rajiformes and Rhinopristiformes [7]. The rostral appendix in skates and guitarfishes is formed de novo on the proximal sides of the growing rostral plate [68]. Meanwhile, according to Miyake et al. [56], the paired “rostral cartilages” that are equivalent to Holmgren’s [69] “rostral appendices”, develop during early ontogeny and arise from the ventromedial area of the lamina orbitonasalis (at least in *Torpedo*). The area of development of these structures in *Torpedo* is topologically similar to the areas where the rostral processes of platyrhinids are formed, and might indicate a homologous relationship between the “lateral rostral cartilages” of Baranes and Randal [70] and Claeson [60], which are present in *Torpedo, Electrolux, Typhlonarke, Temera* and *Narke,* and the “rostral processes” of *Platyrhina* and *Platyrhinoidis* of de Carvalho [67], The presence of rostral processes is unknown (?) in some fossils recognized as sister taxa (i.e., †*Britobatos*) or belonging to Platyrhinidae (i.e., †*Tingitanius*; [23]), but rostral processes are present in the Eocene platyrhinid †*Eoplatyrhina* [33]. We kept holocephalians as unknown (?) as we still have doubts about the homology of the “process” recognized by de Beer and Moy-Thomas [55].

**Ptr (see discussion Parsimony tree):** The rostral processes are a synapomorphy of Torpediniformes.

**MLtr (see discussion Maximum Likelihood tree):** The rostral processes are a shared feature between *Platyrhina, Platyrhinoidis,* †*Eoplatyrhina, Hypnos, Torpedo, Narcine,* †*Titanonarke, Temera* and *Narke*.

8. **Rostral processes (proximal articulation):** (0) Articulated with nasal capsules; (1) continuous with neurocranium; (2) articulated with ventral aspect of rostral cartilage. Based on Claeson [60] (Supporting Information char. 50) and Claeson [71] (char. 48). This character seeks to include the variation on the articulation between the rostral processes and the neurocranium following Marramà et al. [33].

**Figure 8:**
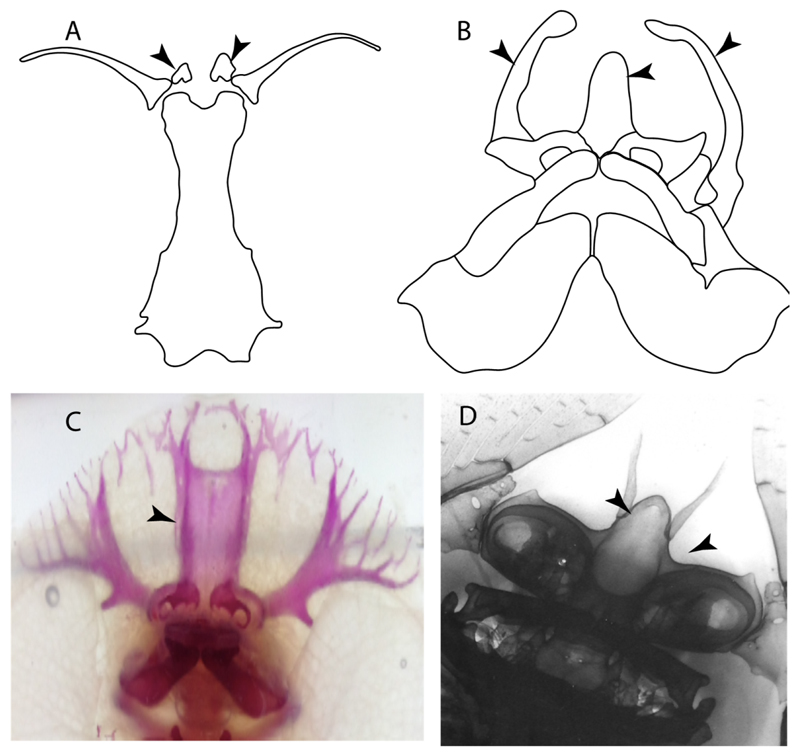
Neurocranium of selected torpediniforms State (0): **(A)** Interpretative line drawing of *Hypnos subnigrum* (MCZ S985, modified from Claeson Text-Figure 3.17A in [71]. State (0 and 1): **(B)** Interpretative line drawing of *Narke* sp. (ZMB 33911, modified from Claeson Text-Figure 3.17J in [71]. State (2): **(C)** *Narcine brasiliensis* (CNPE-IBUNAM 9280); **(D)** *Platyrhina triseriata* (MNHN 4329). Arrowheads indicate the lateral rostral process.

**Ptr and MLtr (see discussion Maximum Likelihood and Parsimony trees):** There is uncertainty in the plesiomorphic-state reconstruction caused by the inapplicable coding in the taxa with no rostral processes. The presence of an articulation between the ventral aspect of the rostral cartilage and the rostral process is a basal feature for Torpediniformes, with subsequent gains of the rostral process’s articulation with time nasal capsule in *Hypnos, Torpedo* and *Temera* (Figure 8A). *Narcine* and †*Titanonarke* retain the plesiomorphic state (articulation with the ventral aspect of the rostral cartilages) (Figure 8C,D), with a tripodal structure of *Narke* (0 and 1) (Figure 8B).

9. **Rostral appendix:** (0) Absent; (1) present. Modified from Aschliman et al. [7] (char. 28). The presence of rostral appendices is considered a shared feature between rhinopristisforms and rajiforms [7]. McEachran et al. [72] and Claeson et al. [23] also recognized their presence in fossil taxa such as †*“Rhinobatos”maronita,* †“*R*.” *latus*, †*Britobatos* and †*Rhombopterygia.* The coding from previous works [23,59] for †*Spathobatis* was changed to “present” after new observations were made on several fossil specimens (e.g., BMNH P. 12067, BSPG 1952-I-82, BSPG -AS-I-505) (EV, pers. observ.). Although rostral appendices might be present in *Diplobatis, Benthobatis, Narcine, Discopyge* and †*Titanonarke* [28,69,73,74], their homology with rostral appendices of skates and guitarfishes is unclear, and we therefore coded the state as (0) in these taxa. Considering that the subtriangular rostral extremity reported and observed in *Urolophus, Gymnura, Urotrygon, Plesiobatis* and *Potamotrygon* is in connection with the anterior medial outgrowth of the trabecula [67] (one of the embryological cartilages that forming the rostral cartilages along with the lamina orbitonasalis), it is very likely that these vestigial cartilages correspond to the rostral node and rostral shaft (sensu McEachran and Compagno [75]) and that the rostral appendices are involved, considering the presence of small posterior projections parallel to the rostral shaft. However, considering the lack of agreement about the presence of this structure in the literature [56,67,72] we coded this character as unknown for these taxa (?).

**Figure 9:**
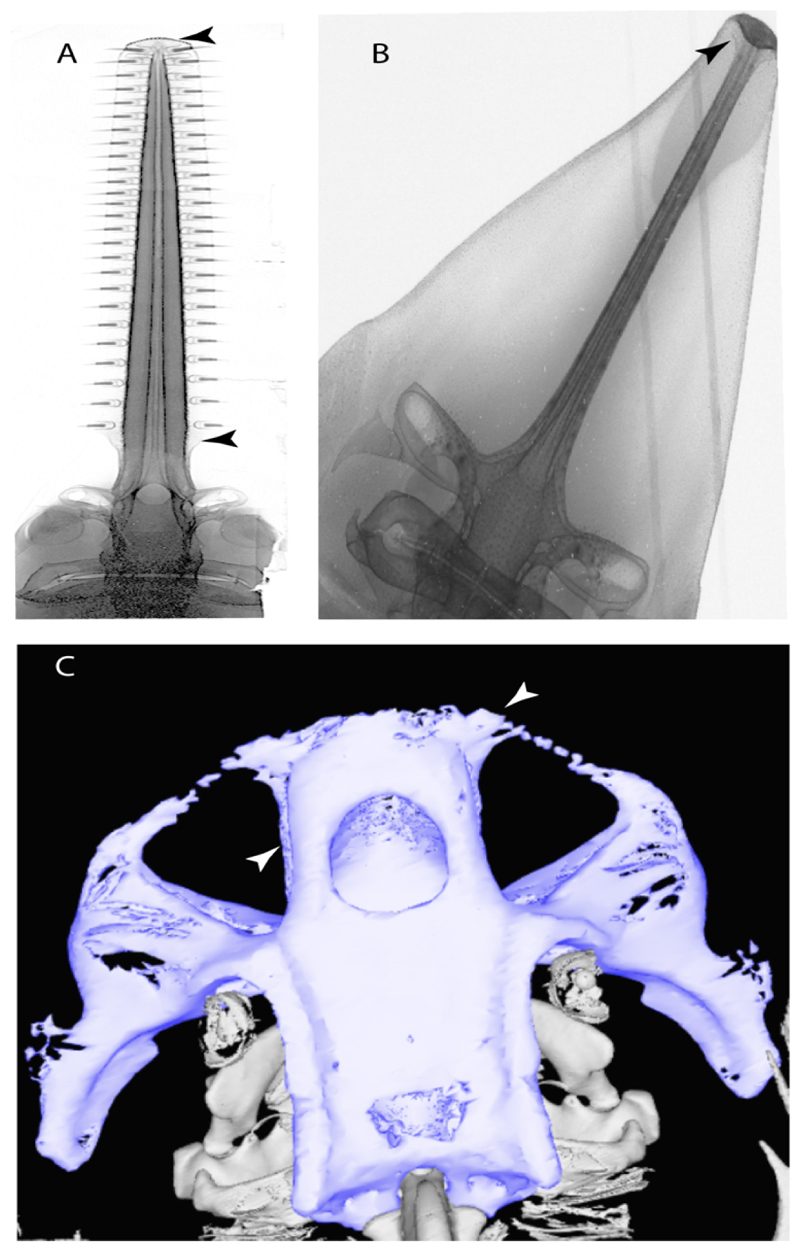
Rostrum of various Rhinopristiformes. State (1): **(A)** *Pristis* sp. (unpublished data); **(B)** *Glaucostegus granulatus* (NHMUK 2012.2.8.54); **(C)** *Rhina ancylostoma* (https://sharksrays.org/ (accessed on 23 March 2020)). Arrowheads indicate the rostral appendix at the base and tip or rostral cartilage.

**Ptr (see discussion Parsimony tree):** The presence of rostral appendices (Figure 9) is a synapomorphy for Rhinopristiformes with independent gains in clade 22 and *Bathyraja* and *Raja.*

**MLtr (see discussion Maximum Likelihood tree):** The presence of rostral appendices is a shared feature of †*Spathobatis*, †*Asterodermus*, †*Kimmerobatis, Bathyraja, Raja*, †*Britobatos*, †“*Rhinobatos” whitfieldi*, †*Rhombopterygia*, †*Iansan*, †*Pseudorhinobatos*, †“*Rhinobatos” hakelensis, Pseudobatos, Rhinobatos, Glaucostegus*, †“*Rhinobatos” maronita, Rhynchobatus*, †“*Rhinobatos” latus, Rhina, Pristis, Aptychotrema*, †*Tlalocbatur,* †*Stahlraja, Zapteryx* and *Trygonorrhina.* The absence of rastral appendices is a synapomorphy for clade 25.

**Figure 10:**
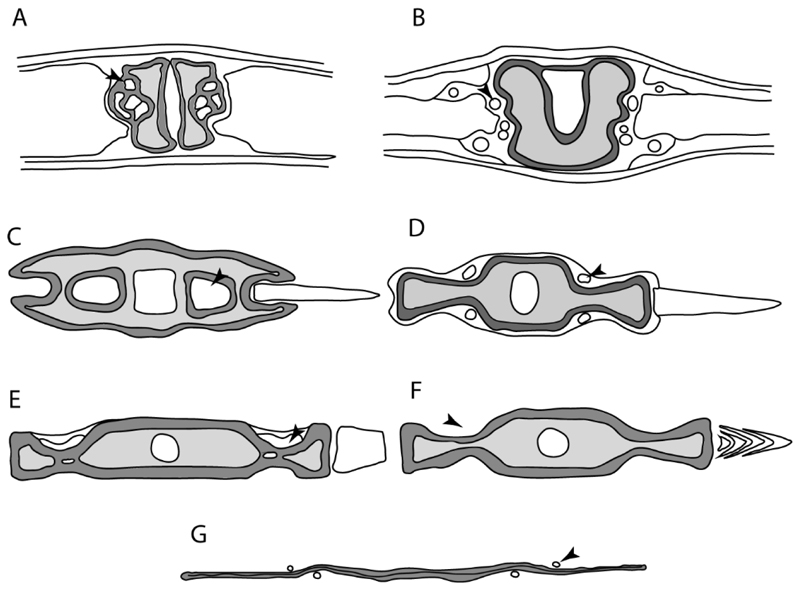
Interpretative line drawings of the lateral face of the neurocranium and sections of the rostral cartilages with the passage of superficial ophthalmic nerve in different chondrichthyan groups State (0): **(A)** of *Rhinobatos typus;* **(B)** *Aptychotrema rostrata* based on Wueringer et al. Text-Figure 4 in [76]; **(C)** *Pristis* sp. (BRC–Pristis). State (1): **(D)** *Pristiophorus nudipinnis* based on Wueringer et al. Text-Figure 4 in [76]; **(E)** *Onchopristis numidus* based on Cappetta Text-Figure 4 in [77] and Wueringer et al. Text-Figure 4, NHMUK PV P 75503 in [76]; **(F)** †*Schizorhiza stromeri* aster Smith et al. Figures 1a–l and 2a–f; NHMUK PV P 73625 in [80]; **(G)** *Libanopristis hiram* based on Cappetta Text-Figure 3 in [77]. Arrowheads indicate the passage of the superficial ophthalmic nerve through the ethmoidal region.

11. **Rostral passage of superficial ophthalmic nerve:** (0) Covered; (1) open. Based on Wueringer et al. [76] and Cappetta [77]. In *Chimaera* and *Harriotta,* the ramus of the superficial ophthalmic nerves runs across the anterior and posterior opening of the ethmoidal canal and is covered by the lamina orbitonasalis anteriorly and by the orbital cartilage posteriorly [55]. Coding for *Chlamydoselachus* follows Allis’s [78] observations, coding in *Torpedo* follows Ewart’s [79] observations. *Rhinobatos, Aptychotrama* and *Pristis* were used to illustrate the state found in most batomorphs [76] (Figure 10A–C). In the rostral cartilage of sclerorhynchoids, the supraophthalmic nerve canal is open, like in *Pristiophorus* (Figure 10D–G) [76,77].

**Ptr and MLtr (see discussion Maximum Likelihood and Parsimony trees):** An open rostral passage of the superficial ophthalmic nerve is a synapomorphy of the †Sclerorhynchoidei clade, with independent gains in *Pristiophorus* and *Torpedo.*

**Figure 11:**
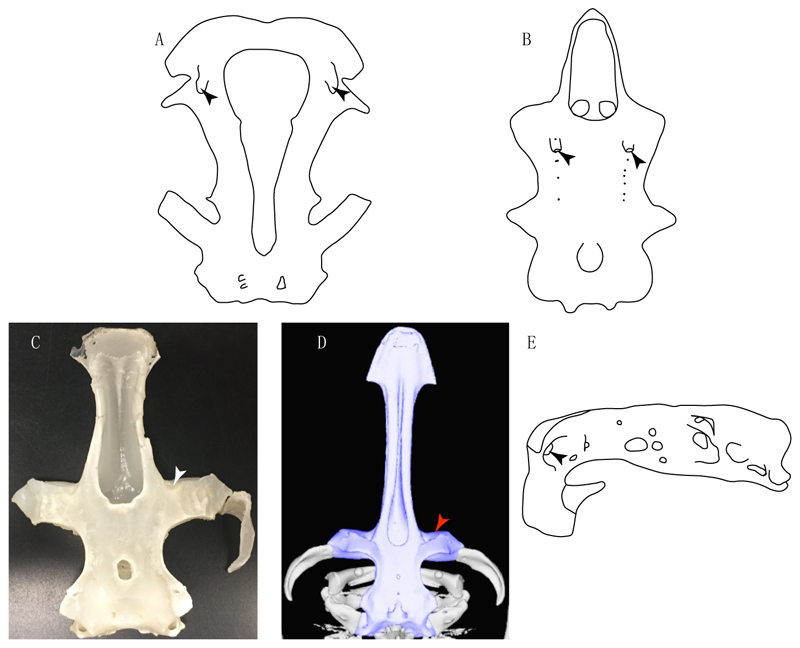
Neurocranium of selected Neoselachii. State (0): **(A)** *Potamotrygon mototro* (AMNH 97428, https://sharksrays.org/ (accessed on 23 March 2020)); **(B)** *Squalus acanthias* based on Thomas et al. Text-Figure 1 in [81]. State (1): (**C**) *Zapteryx exasperate* (CNPE-IBUNAM 20528); **(D)** *Aptychotrema rostrata* (CSIRO 101, https://sharksrays.org/ (accessed on 15 May 2020)); **(E)** *Myliobatis tobijei* based on Nishida Text-Figure 19 in [82]. Arrowheads indicate the position of the preorbital foramen.

12. **Anterior preorbital foramen:** (0) Dorsally located; (1) anteriorly located. Modified from Aschliman et al. [7] (char. 35); Villalobos-Segura et al. [32] (char. 37). We modified the previous coding for Rhinopristiformes, as the foramina in †*Spathobatis*, †*Stahlraja*, †*Tlalocbatus, Pristis, Rhynchobatus, Glaucostegus*, †*“Rhinobatos” latus, Rhina, Rhinobatos, Pseudobatos*, †*Eorhinobatos*, †*Pseudorhinobatos*, †*Iansan, Trygonorrhina, Zapteryx* and *Aptychotrema* are located near the base of the rostral cartilage, but more anteriorly directed in *Myliobatis, Aetobatus, Rhinoptera* and *Mobula* (Figure 11C–E). Aschliman et al. [7] coding was kept, except for *Chimaera, Harriotta, Temera* and *Torpedo,* as we could not observe the foramen (?).

**Ptr and MLtr (see discussion Maximum Likelihood and Parsimony trees):** An anterior preorbital foramen located anteriorly is a synapomorphy of Rhinopristiformes with an independent gain in the clade [*Hemiscyllium + Ginglymostoma*], in clade 16 and †*Spathobatis.*

**MLtr (see discussion Maximum Likelihood tree):** An anterior preorbital foramen located anteriorly is recovered as a synapomorphy of the clade [*Hemiscyllium* + *Ginglymostoma*] with independent gains in †*Spathobatis* and being the basal state in clades 10,10′ and 14.

13. **Preorbital process:** (0) Present; (1) absent. Modified from Aschliman et al. [7] (char. 33), based on a new re-interpretation of the specimens in the literature [24] and a reexamination of the specimens (BSP AS 1952-I-82 and AS-I-505), the coding of Villalobos-Segura et al. [32] for †*Kimmerobatis* and †*Spathobatis* was changed from absent (1) to present (0).

**Ptr and MLtr (see discussion Maximum Likelihood and Parsimony trees):** The presence of a postorbital process is a synapomorphy for the Euselachii clade, with subsequent independent losses in the clade [*Mobula* + *Rhinoptera*] and *Temera*, †*Rhombopterygia, Holocephali*, †*Doliodus*, †*Ozarcus* and †*Cobelodus.*

34. **Nasal capsules:** (0) Laterally expanded; (1) ventrolaterally expanded; (2) anterolaterally expanded; (3) prolonged interorbitonasal region, which forms a pedicel (“trumpet shaped nasal capsule”). Aschliman et al. [7] (char. 31) was modified, adding Maisey et al.’s [83] (char. 4) observations on the nasal capsules in *Squatina* and †*Pseudorhina* (2) and Compagno’s [7] and Shirai’s [35,37] characterization of these structures in the orectolobids and heterodontids (3).

**Figure 12:**
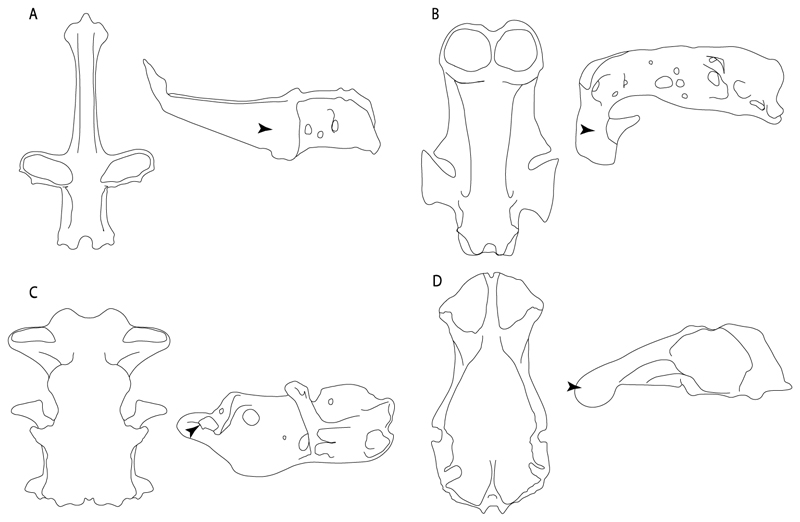
Interpretative drawings of ventral and lateral view of the neurocranium in ventral and lateral view. State (0): **(A)** *Rhinobatos glaucostigma* (CNPE-IBUNAM 17810). State (1): **(B)** *Myliobatis tobijei* redrawn from Nishida Text-Figures 16A and 19D in [82]. State (2): **(C)** *Squatina nebulosa* (AMNH 258172, https://sharksrays.org/ (accessed on 5 May 2020)). State (3): **(D)** *Heterodontus francisci.* (AMNH 217862). Arrowheads indicate the nasal capsules.

**Ptr and MLtr (see discussion Maximum Likelihood and Parsimony trees):** Laterally expanded nasal capsules (Figure 12A) are the plesiomorphic state for chondrichthyans, with the subsequent gain of trumpet-shaped nasal capsules (Figure 12D) as a synapomorphy of clade 2. The anterolateral expansion of the nasal capsules (Figure 12C) is a synapomorphy of [†*Pseudorhina* + *Squatina],* and the ventrolateral expansion of the nasal capsules (Figure 12B) is a synapomorphy for clade 18.

**Ptr (see discussion Parsimony tree):** There is an independent gain of the ventrolateral expansion of the nasal capsules representing the plesiomorphic feature of clade 14, except for *Hexatrygon* and *Plesiobatis,* which present the basal state.

**MLtr (see discussion Maximum Likelihood tree):** It presents a similar-state reconstruction for this feature. However, the additional gain of the ventrolateral expansion of the nasal capsules is a synapomorphy for clade 29.

35. **Nasal capsule margin:** (0) Straight; (1) horn-like process. Based on Villalobos-Segura et al. [32] (char. 83).

**Figure 13:**
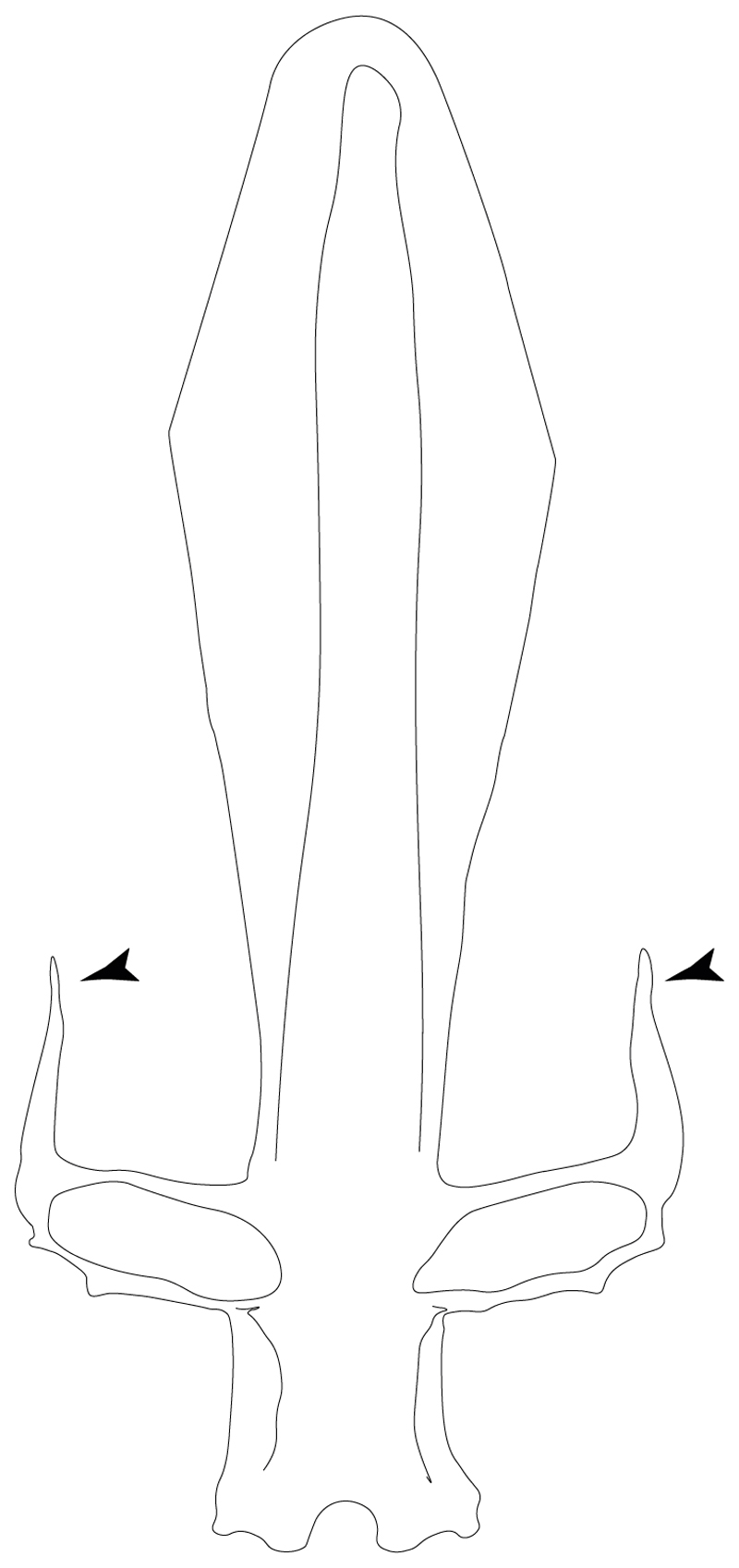
Interpretative drawings of ventral view of the neurocranium in ventral view of †“*Rhinobatos*” *maronita* (MNHN 1946.17.274): State (1). Arrowheads: horn-like process of nasal capsules.

**Ptr (see discussion Parsimony tree):** The presence of a horn-like process (Figure 13) is a synapomorphy of clades 11 and 24. There are independent gains of the horn process in †*Tlalocbatus*, †*Stahlraja* and †*Britobatos.*

**MLtr (see discussion Maximum Likelihood tree):** Presents a similar reconstruction for this character as in the parsimony tree, but in this case, the paraphyletic state of the thornback clade makes the recovery of this feature as a synapomorphy for the group impossible.

110. **Position of the articulation of the antorbital cartilage on nasal capsule:** (0) Lateral, (1) anterolateral; (2) posterolateral. Modified from de Carvalho [67] (char. 2).

**Figure 14:**
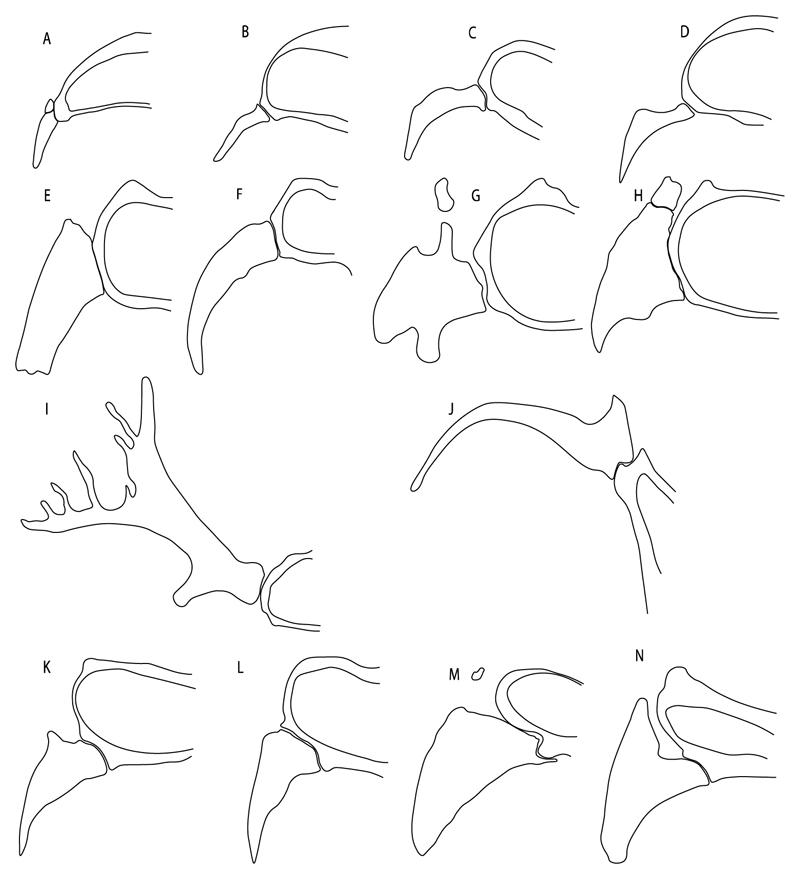
Interpretative drawings of ventral views of the nasal capsules and antorbital cartilages in ventral views. State (0): **(A)** *Zanobatus* sp. (MNHN 1989. 12. 91); **(B)** *Urotrygon chilensis* (FMNH 93737) redrawn and modified from de Carvalho Text-Figure 33B in [67]; **(C)** *Raja clavata* (BRC–Raja); **(D)** *Bathyraja leucomelanos* (MNHN 2005-2740) redrawn and modified from Iglésias and Hartmann Text-Figure 11 in [84]; **(E)** *Zapteryx xyster* (CNPE IBUNAM 16661); **(F)** *Aptychotrema vincentiana* (CSIRO 101, https://sharksrays.org/ (accessed on 12 April 2020)); **(G)** *Platyrhinoidis triseriata* (MNHN 3211); **(H)** *Platyrhina sinensis* (MNHN 1307). State (1) **(I)** *Narcine brasiliensis* (AMNH 77069, https://sharksrays.org/ (accessed on 12 April 2020)); **(J)** *Torpedo fuscomarulata* (USNM, https://sharksrays.org/ (accessed on 12 April 2020)). State (2) **(K)** *Glaucostegus granulatus* (NHMUK 2012.2.8.54); **(L)** *Rhinobatos productus* (CNPE-IBUNAM 17829); **(M)** *Pristis pristis* (CAS-SU 12670); **(N)** *Rhynchobatus springeri* (https://sharksrays.org/ (accessed on 12 April 2020)).

**Ptr and MLtr (see discussion Maximum Likelihood and Parsimony trees):** The lateral position of the articulation between the antorbital cartilage and the nasal capsule (Figure 14A–H) is the plesiomorphic condition for batomorphs, being present among several taxa and groups: Rajiformes (*Raja, Bathyraja,* †*Ostarriraja,* †*Cyclobatis,* †*Ptychotrygon,* †*Sclerohynchus,* †*Libanopristis,* †*Asflapristis* and †*Onchopristis;* Torpediniformes: *Platyrhina,* †*Eoplatyrhina, Platyrhinoidis* and †*Tingitanius*); Rhinopristiformes (†*Stahlraja,* †*Tlalocbatus, Trygonorrhina, Zapteryx, Aptychotrema* and †*Britobatos*); Myliobatiformes (*Zanobatus,* †*Plesiozanobatus,* †*Asterotrygon,* †*Heliobatis, Urolophus, Urobatis, Urotrygon, Plesiobatis, Hexatrygon, Hypanus, Potamotrygon, Neotrygon, Gymnura, Myliobatis, Aetobatus, Rhinoptera, Mobula,* †*Arechia,* †*Lessiniabatis,* †*Tethytrygon,* †*Arechia,* †*Lessiniabatis,* †*Promyliobatis* and †*Tethytrygon*); Jurassic batomorphs (†*Kimmerobatis,* †*Asterodermus,* †*Spathobatis* and †*Belemnobatis).* An anterolateral position of the articulation between the antorbital cartilage and the nasal capsules (Figure 14I,J) is a synapomorphy of clade 18. The posterolateral placement of the articulation between the antorbital cartilage and the nasal capsules (Figure 14K–N) is a synapomorphy of clade 10.

**Figure 15:**
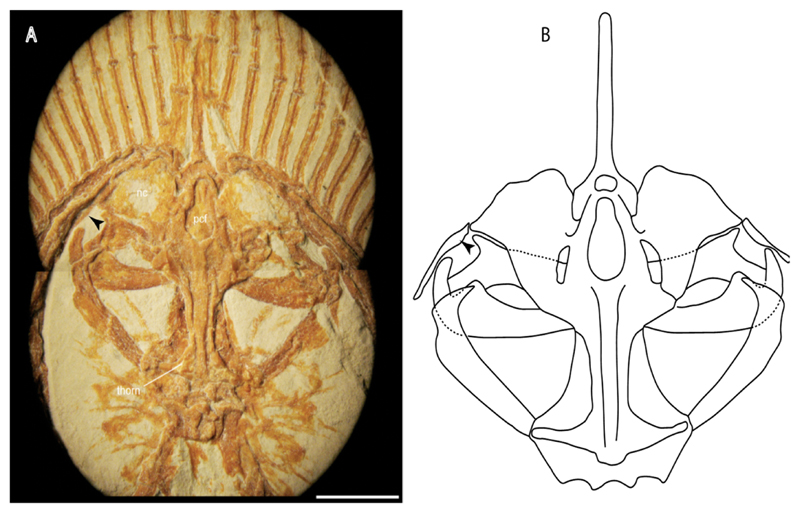
Dorsal surface of neurocranium in **(A)** †*Cyclobatis* sp. (MNHN HAK 550) Text-Figure 5.5 in [80]; **(B)** Interpretative line drawing of †*Cyclobatis major* (MNHN 1939-13-334A) and †*Cyclobatis* sp. (MNHN HAK 550) based on Cappetta [85]. Arrowheads: antorbital cartilage.

23. **Antorbital cartilages:** (0) Absent; (1) present. Modified from Aschliman et al. [7] (char. 83). Shirai [35] suggests the presence of antorbital cartilages in *Pristiophorus.* However no evidence of these cartilages was observed in the *Pristiophorus* specimens CSIRO 3731 and CAS 4942. The previously illustrated lack of antorbital cartilages in †*Cyclobatis* in Cappetta Text-Figure 355A in [85] seems to be caused by the position of these cartilages, which appear to be overlapped by the propterygium, like in stingrays (Figure 15).

**Ptr and MLtr (see discussion Maximum Likelihood and Parsimony trees):** The presence of antorbital cartilages is recovered as a synapomorphy of batomorphs.

24. **Antorbital cartilage (shape):** (0) Triangular-shaped with regular outline; (1) variously shaped and with an irregular outline. Modified from Villalobos-Segura et al. [32] (char. 9), based on observations on †*Titanonarke, Narcine, Narke, Temera*, †*Eoplatyrhina, Platyrhinoidis* and †*Tingitanius,* which present antorbital cartilages with an irregular outline and various projections. Due to preservation or damage, †*Kimmerobatis*, †*Promyliobatis* and †*Ischyrhiza* are coded as (?).

**Figure 16:**
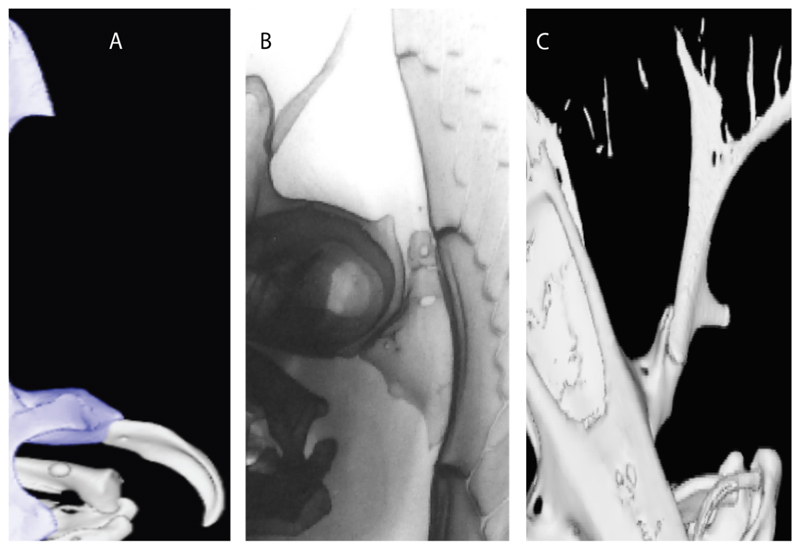
Portion or the neurocranium and antorbital cartilages. State (0): **(A)** *Aptychotsema vincentiana* (CSIRO 101, https://sharksrays.org/ (accessed on 25 May 2020)). State (0): **(B)** *Platyrhina sinensis.* (MNHN 1307). State (1) **(C)** *Narcine brasiliensis* (AMNH 77069, https://sharksrays.org/ (accessed on 25 May 2020)).

**Ptr (see discussion Parsimony tree):** Irregular-shaped antorbital cartilages (Figure 16) are a synapomorphy with independent gains in clades 25 and 19.

**MLtr (see discussion Maximum Likelihood tree):** The presence of irregular-shaped antorbital cartilages is a synapomorphy of clade 26.

25. **Antorbital cartilages (with regular outline):** (0) Well-developed; (1) reduced. Modified from Villalobos-Segura et al. [32] (char. 9), split in two characters (25 and 26). This character includes the variation observed in the size of the antorbital cartilages with regular outlines in batomorphs. Taxa with irregular outlines of antorbital cartilages (i.e., †*Titanonarke, Narcine, Narke, Temera*, †*Eoplatyrhina, Platyrhinoidis* and †*Tingitanius*) and taxa lacking antorbital cartilages (i.e., holocephalians and sharks), were coded as inapplicable (-). Due to taphonomic loss or damage in †*Kimmerobatis*, †*Promyliobatis* and †*Ischyrhiza,* this character is unknown (?).

**Ptr and MLtr (see discussion Maximum Likelihood and Parsimony trees):** The presence of small antorbital cartilages is a synapomorphy for the Myliobatiformes, with an independent gain in †*Cyclobatis.*

26. **Anterior process of antorbital cartilage (if regular outline):** (0) Absent; (1) present. This character includes the variation observed in the anterior portion of the antorbital cartilage of batomorphs.

**Ptr and MLtr (see discussion Maximum Likelihood and Parsimony trees):** The presence of an anterior process in the antorbital cartilages is a synapomorphy of clade 16 with independent gains in *Platyrhina,* †“*Rhinobatos”whitfieldi* and *Zanobatus.*

27. **Postorbital precess:** (0) Well-developed; (1) reduced. Based on Claeson et al. [23] (char. 12). This process cannot be observed in †*Rhombopterygia*, †*Ischyrhiza* and †*Lessiniabatis* (?).

**Figure 17:**
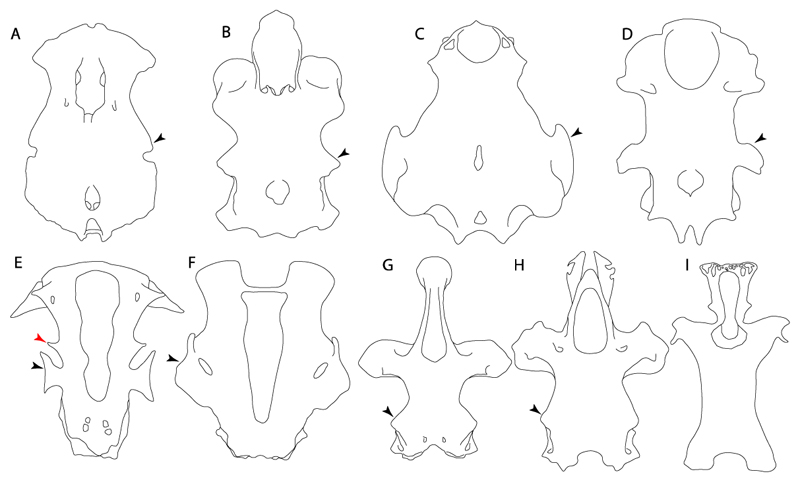
Interpretative drawings of the neurocranium of various chondrichthyans in dorsal view. State (0): **(A)** *Heterodontus francisci*. (AMNH 217862); **(B)** *Squalus acanthias* redrawn from Thomas et al. Text-Figure 1 in [81] and Maisey Text-Figure 15A in [86]; **(C)** †*Egertonodus* (†*Hybodus*) *basanus* redrawn and modified from Maisey Text-Figure 15C in [86]; **(D))** *Chlamydoselachus africana* (SAM 36076, redrawn and modified from Ebert and Compagno Text-Figure 4 in [87]; **(E)** *Gymnura japonica* (HUMZ 4830), redrawn and modified from Nishida Text-Figure 15A in [82]; **(F)** *Rhinoptera javanica* (HUMZ 97698), redrawn and modified from Nishida Text-Figure 17A in [82]; **(G)** *Trygonorrhina fasciata* (MCZ 982S), after McEachran et al. Text-Figure 7 in [38]; **(H)** *Platyrhinoidis* sp. redrawn and modified from Nishida Text-Figure 7G in [82]. State (1): **(I)** *Torpedo ocellata* (AMNH 4128). Black arrowheads: postorbital process; red arrowheads: triangular process.

**Ptr and MLtr (see discussion Maximum Likelihood and Parsimony trees):** A reduced postorbital process is recovered (Figure 17I) as a synapomorphy of clade 16, with independent gains in †*Plesiozanobatus*,, †*Ostarriraja, Chimaera, Harriotta* and †*Ozarcus.*

28. **Postorbital process:** (0) Narrow; (1) broad and shelf-like. Taken from Aschliman et al. [7], (char. 36). †*Cyclobatis* presents a very narrow and laterally projected postorbital process.

**Figure 18:**
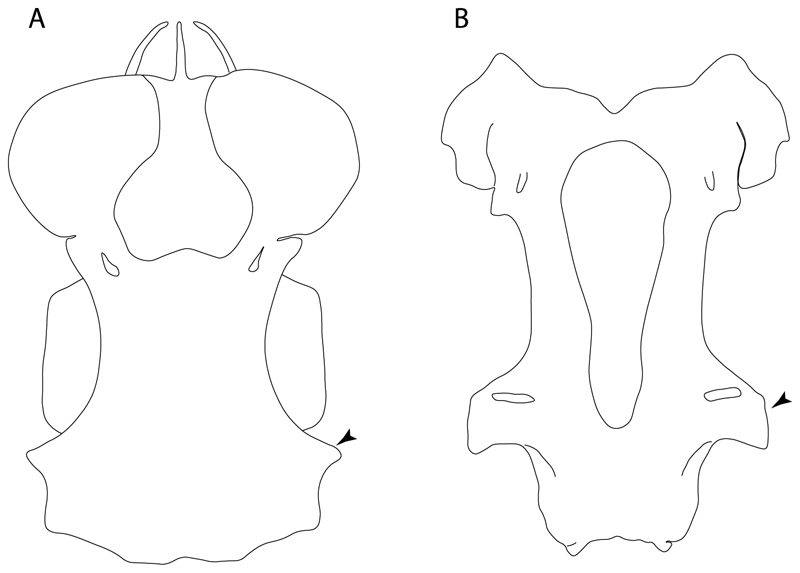
Interpretative drawings of the neurocranium in dorsal view. State (0): **(A)** *Scyliorhinus cabofriensis* (UERJ 2231.4) redrawn and modified from Soares et al. Text-Figure 7A in [88]. State (1): **(B)** *Plesiobatis daviesi* redrawn and modified from Nishida Text-Figure 10 in [82]. Arrowheads: postorbital process.

**Ptr and MLtr: (see discussion Maximum Likelihood and Parsimony trees):** A broad shelf-like postorbital process (Figure 18B) is independently a gain as well as a synapomorphy for the Hybodontiformes and clade 14, and with additional independent gains as the basal feature in clade 3 and in †*Britobatos* as an autapomorphy.

**MLtr (see discussion Maximum Likelihood tree):** A broad postorbital process is a synapomorphy for members of clade 3. Consequently, the narrow state in *Pristiophorus* is interpreted as an independent gain and thus an autapomorphy.

40. **Suborbital shelf:** (0) Absent; (1) present. Based on observations by Shirai [35,37] and Klug [20]. The suborbital shelf is a horizontal plate on the ventral junction of the orbital wall and basal plate that is the floor of the orbit. It runs from the nasal capsule to the otic capsule and is penetrated posteriorly by the stapedial foramen and sometimes laterally by a notch, foramen, or fenestra for the palatine branch of the facial nerve.

**Figure 19:**
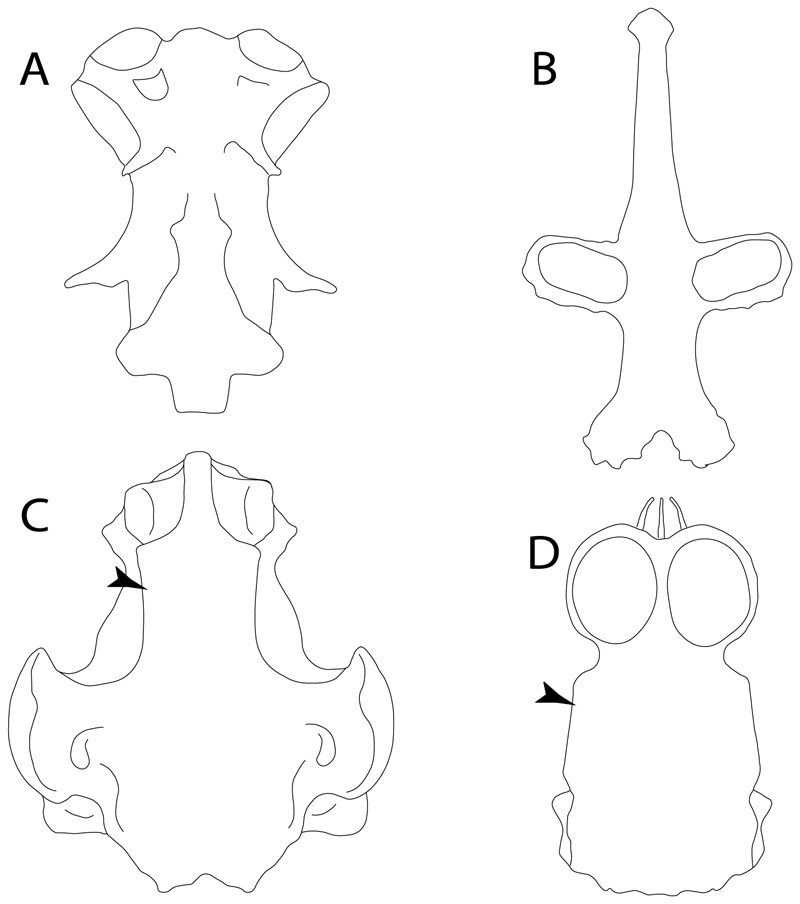
Interpretative drawings of the neurocranium in ventral view. State (0): **(A)** *Hexanchus nakamurai* (DAE 881504) redrawn and modified from Ebert et al. Text-Figure 8B in [89]; **(B)** *Glaucostegus granulatus* (NHMUK 2012.2.8.54). State (1): **(C)** †*Hybodus* (†*Egertonodus*) *basanus* redrawn and modified from Maisey Text-Figure 9B in [85]; **(D)** *Scyliorhinus cabofriensis* (UERJ 2231.4) redrawn from Soares et al. Text-Figure 7B in [87]. Arrowhead: suborbital shelf.

**Ptr and MLtr (see discussion Maximum Likelihood and Parsimony trees):** The presence of a suborbital process (Figure 19A,B) is a synapomorphy for Galeomorphii, with independent gains in [†*Hybodus* + †*Hamiltonichthys*] and †*Doliodus.*

41. **Basitrabecular process:** (0) Absent (1) present. Based on de Carvalho [38] (char. 11), de Carvalho and Maisey [15] (char. 21) and Klug [20] (char. 10). This character is interpreted as a separate feature from the suborbital shelf based on its topographic relationships and development [90]. The basitrabecular process derives from a lateral expansion of the polar cartilage just anterior to the auditory capsules and articulates anteriorly with the orbital process of the palatoquadrate [15,90,91].

**Figure 20:**
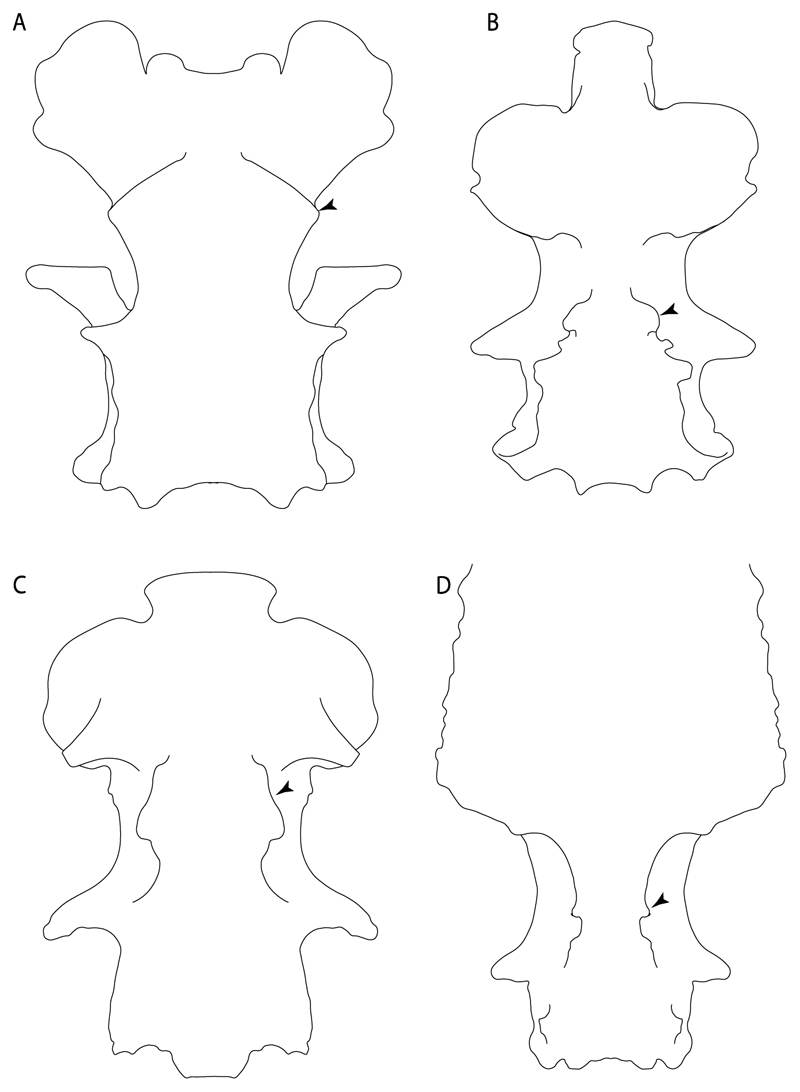
Interpretative drawings of the neurocranium in ventral view. State (1): **(A)** *Squatina japonica* (HUMZ 91670) redrawn and modified from Shirai plate 13A in [35]; **(B)** *Squalus acanthias* (GMBL 7313, https://sharksrays.org/ (accessed on 14 April 2020)); **(C)** *Chlamydoselachus anguineus* (MSM-88-40) redrawn and modified from Shirai plate 1B in [35]; **(D)** *Pristiophorus nudipinnis* (CSIRO 3731, https://sharksrays.org// (accessed on 14 April 2020)). Arrowhead: basitrabecular process.

**Ptr and MLtr (see discussion Maximum Likelihood and Parsimony trees):** The presence of a basitrabecular process (Figure 20A–D) is a synapomorphy for clade 3.

#### 3.1.4 Viceral Arches

14. **Jaw support:** (0) Holostyly; (1) hyostyly; (2) archaeostylic. Based on observations by Maisey [92] and Wilga and Ferry [93]. In holocephalians, the neurocranium and the palatoquadrate are fused (i.e., holostylic jaw suspension). The term archaeostylic (*sensu* Maisey [94]) refers to those taxa with a postorbital articulation on the ventrolateral part of the lateral commissure. Both †*Cobelodus* and †*Ozarcus* lack a distinct hyomandibular facet [18,94], indicating a loose attachment to the neurocranium. Hybodontiforms and elasmobranchs present various articulation patterns between the upper jaw (palatoquadrate) and the neurocranium, but all share a close inferaction with the hyomandibula (i.e., hyostylic) [87].

**Ptr and MLtr (see discussion Maximum Likelihood and Parsimony trees):** The archaeostylic state is the plesiomorphic condition for chondrichthyans and present in †*Doliodus*, †*Ozarcus* and †*Cobelodus.* There is uncertainty regarding the nodal state for holocephalians and euselachians, as holocephalians present a holostylic support as their plesiomorphic condition, while it is hyostylic in euselachians.

15. **Ethmoidal articulation:** (0) Absent; (1) present. Modified from Shirai [35] (char. 11), based on observations by Maisey [92,94], Lane and Maisey [21] and Wilga and Ferry [93].

†*Doliodus* and †*Cobelodus* were coded as in Pradel et al. [13] (char. 25), and †*Ozarcus* was coded according to Pradel et al. [22]. In Holocephali this character is inapplicable (-), considering the fusion of the palatoquedrate to the neurocranium.

**Figure 21:**
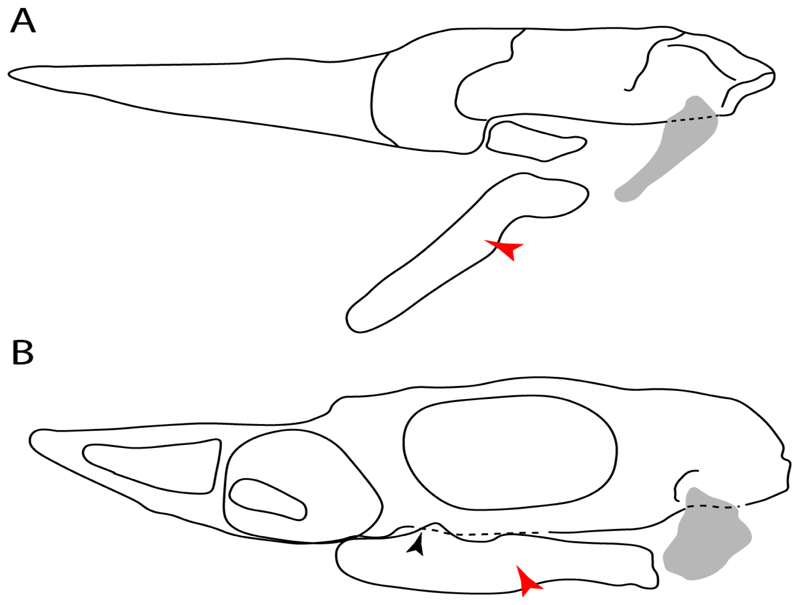
Interpretative drawings of the neurocranium of selected neoselachians in lateral view. State (0): **(A)** *Raja* sp. based on Maisey Text-Figure 6 in [92]. State (1). **(B)** *Mustelus manazo* (https://sharksrays.org/ (accessed on 26 May 2020)). Red arrowheads indicate the palatoquadrate; black arrowhead indicates the ethmoidal articulation. Hyomandibula in gray color.

**Ptr and MLtr (see discussion Maximum Likelihood and Parsimony trees):** An ethmoidal articulation (Figure 21A) is an independent gain and a synapomorphy of the clades 15 and [†*Hybodus* + †*Hamiltonichthys*]).

17. **Postorbital articulation:** (0) Absent; (1–2) present. Modified from Klug [20] (char. 11), based on observations by Maisey [92]. An additional character state is proposed to include the variation observed in †*Doliodus,* symmoriids (2) and hexanchids (1).

**Ptr and MLtr (see discussion Maximum Likelihood and Parsimony trees):** The presence of a postorbital articulation located further posteroventroleterally of the chondrified lateral commissure is recovered as the basal state for chondrichthyans, being present in †Doliodus, †*Cobelodus* and †*Ozarcus*, with the subsequent gain of a postorbital articulation where the articulation surface is in the proximal part of the process in *Hexanchus* (Figure 15).

**Figure 22:**
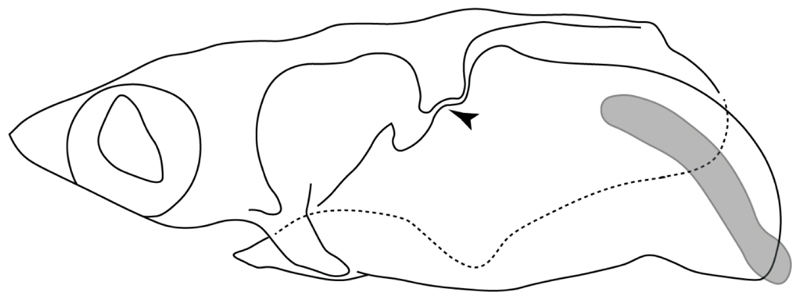
State (1): Interpretative drawing of the neurocranium of *Heptranchias perlo* redrawn and modified from Maisey Text-Figure 3C in [92]. Arrowhead: Postorbital articulation. Hyomandibula in gray color.

**MLtr:** The absence of a postorbital articulation is a synapomorphy of holocephalians and Euselachii, with a subsequent gain of the articulation in *Hexanchus* (Figure 22).

**Figure 23:**
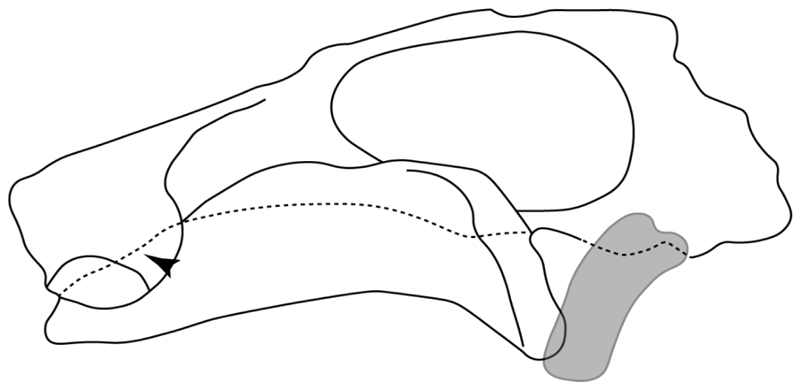
State (1): Interpretative drawing of neurocranium and palatoquadrate of *Heterodontus francisci* redrawn and modified from Maisey Text-Figure 6D in [92]. Arrowhead: ethmoidal articulation. Hyomandibula in gray color.

18. **Downturned ethmoidal articulation:** (0) Absent; (1) present. Based on de Carvalho [38] (char. 4) and Klug [20] (char. 3). In *Heterodontus* and orectolobids, the ethmoidal articulation is modified, retaining a downturned embryonic posture [68,92–96] (Figure 23).

**Ptr and MLtr (see discussion Maximum Likelihood and Parsimony tregs):** The presence of a downwardly directed ethmoidal articulation postorbital articulation is a synapomorphy of clade 2.

**Figure 24:**
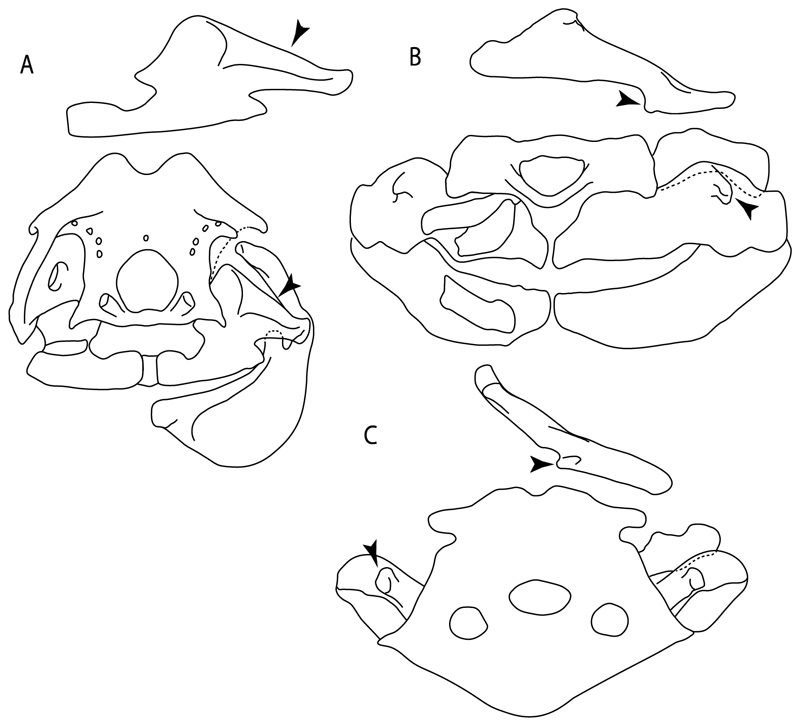
Interpretative drawings of neurocranium and dorso-frontal view of left antimere of palatoquadrate. State (1): **(A)** †*Egertonodus* (†*Hybodus*) *basanus* redrawn and modified from Maisey Text-Figure 3 in [98]; **(B)** *Squatina nebulosa* (AMNH 258172, https://sharksrays.org/ (accessed on 5 May 2020)); **(C)** *Pristiophorus nudipinnis* (CSIRO 3731, https://sharksrays.org/ (accessed on 5 May 2020)) Arrowheads: Quadrate flange (quadrate process).

19. **Quadrate flange:** (0) Absent; (1) present. Modified from Maisey et al. [83] (char. 6). The flange on the palatoquadrate is a characteristic feature in hybodontiforms (Figure 24A). This ledge is located laterally to the mandibular cartilage and does not interact with the Meckel’s cartilage. This process corresponds to the “quadrate process” of de Carvalho et al. [97] and Maisey et al. [83] in squatinids and pristiophorids (Figure 24B,C).

**Ptr and MLtr (see discussion Maximum Likelihood and Parsimony trees):** The presence of this ledge-like process in the palatoquadrate is an independent gain and shared feature for Hybodontiformes and clade 5 (Figures 1 and 2).

44. **Basihyal:** (0) Present; (1) absent. Modified from Aschliman et al. [40] (char. 48), Villalobos-Segura et al. [32] (char. 48)) and Claeson et al. [23] (char. 27). Previous analyses placed both basihyal and first hypobranchial together in a single character, resulting in a mix of neomorphic and transformational characters. We therefore propose independence among these structures, providing separate characters for their presence/absence and interaction (char. 45–46).

**Ptr and MLtr (see discussion Maximum Likelihood and Parsimony trees):** The lack of a basihyal is a synapomorphy of clade 18, with independent losses in †Ozarcus, *Myliobatis, Aetobatus, Rhinoptera.* and *Mobula.*

39. **Fourth hypobranchial:** (0) Well-developed; (1) reduced (new).

**Figure 25:**
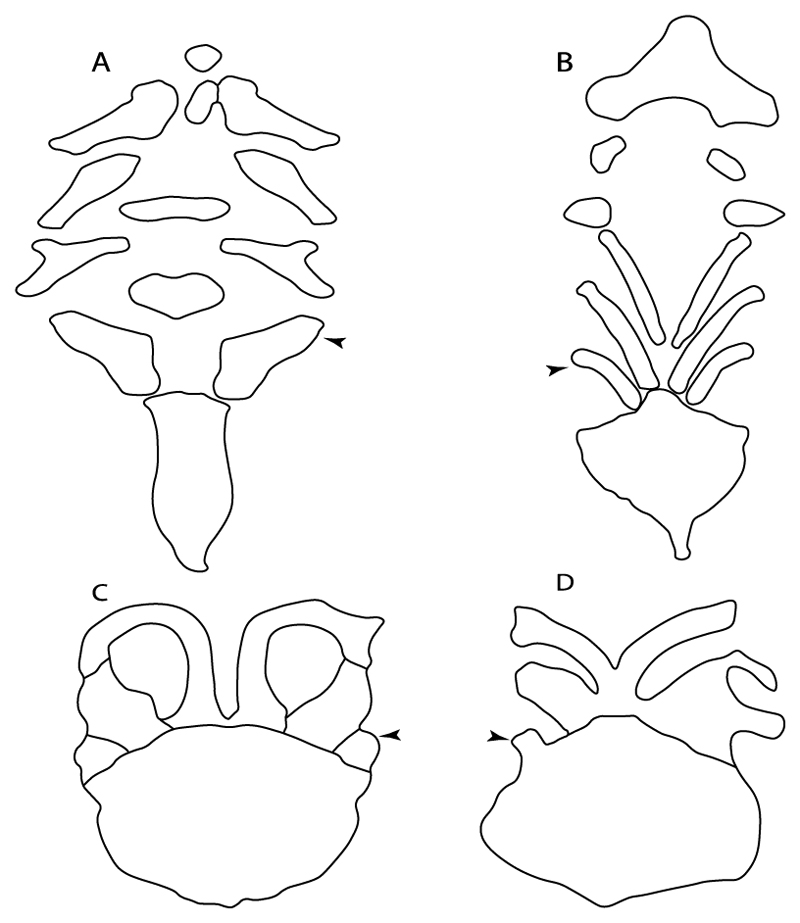
Interpretative drawings of basibranchial and hypobranchials in ventral view. State (0): **(A)** *Callorhinchus capensis* (ANSP 174852) redrawn and modified from de Carvalho et al. Text-Figure 9A in [99]; **(B)** *Hemiscyllium ocellatum* (AMNH 38151) redrawn and modified from de Carvalho et al. Text-Figure 9E in [99]. State (1): **(C,D)** *Zapteryx exasperata* (CNPE-IBUNAM 20528), †*Spathobatis moorbergensis* (BHN 2Pl) redrawn and modified from Cavin Text-Figure 4 in [100] Arrowhead: fourth hypobranchial.

**Ptr and MLtr (see discussion Maximum Likelihood and Parsimony trees):** A well-developed fourth hypobranchial (Figure 25A) is the plesiomorphic state for chondrichthyans. The reduction of the fourth hypobranchial (Figure 25B) is a synapomorphy of batomorphs.

37. **Basibranchial:** (0) Segmented; (1) unsegmented (new). According to Shirai [35] *Pristiophorus* presents an unsegmented basibranchial.

**Figure 26:**
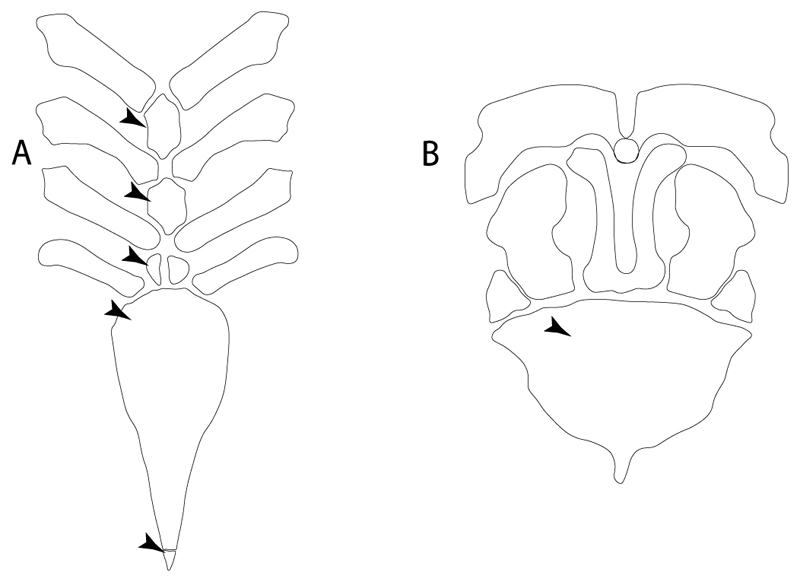
Interpretative drawings of basibranchial and hypobranchials in ventral view. State (1): **(A)** *Heterodontus zebra* (HUMZ 37666) redrawn from Shirai plate 32D in [35]. State (0): **(B)** *Rhynchobatus djiddensis* (MCZ 806) redrawn and modified from Miyake and McEachran Text-Figure 5D in [101] Arrowheads: basibranchial.

**Ptr and MLtr (see discussion Maximum Likelihood and Parsimony trees):** An un-segmented basibranchial (Figure 26B) is recovered additionally as a synapomorphy for batomorphs with independent gains in *Pristiophorus* and *Hemiscyllium.*

#### 3.1.5 Jaws and Branchial Muscles

65. **Spiracularis:** (0) Undivided; (1) divided. Modified from Aschliman et al. [7] (char. 85), divided here into two separate characters, 65 and 66.

**Ptr and MLtr (see discussion Maximum Likelihood and Parsimony trees):** The presence of a divided spiracularis is an independent gain and a synapomorphy of clades 14 and 18.

66. **Spiracularis (if divided):** (0) One bundle enters the dorsal oral membrane underlying the neurocranium; (1) spiracularis splits into lateral and medial bundles, with the medial bundles inserting onto the posterior surface of the Meckel’s cartilage and the lateral bundle onto the dorsal edge of the hyomandibula; (2) spiracularis subdivided proximally and inserts separately into the palatoquadrate and the hyomandibula. This character is proposed to include the character states recognized by Aschliman et al. [7] (char. 85) except for the third state, which seems to be a variation of the first state (splits into lateral and medial bundles).

**Ptr and MLtr (see discussion Maximum Likelihood and Parsimony trees):** A divided spiracularis, in which one muscle bundle enters the dorsal oral membrane underlying the neurocranium, is a shared feature of *Torpedo, Hypnos, Narcine, Narke* and *Temera*. The spiracularis splits into lateral and medial bundles, with the medial bundle inserting onto the posterior surface of the Meckel’s cartilage and the lateral bundle onto the dorsal edge of the hyomandibula, which is a shared feature in *Urolophus, Urobatis, Urotrygon, Plesiobatis, Hypanus, Potamotrygon* and *Neotrygon.* There is a subsequent gain of the spiracularis sub-divided proximally, inserting separately onto the palatoquadrate and the hyomandibula, *Rhinoptera.*

68. **Coracohyomandibularis:** (0) Single origin; (1) separate origins. Modified from Aschliman et al. [7] (char. 88). The character is separated here into two separate characters, 68 and 69, aiming to increase the grouping information on the separate origins of the coracohyomandibularis.

**Ptr (see discussion Parsimony tree): Ptr (see discussion Parsimony tree):** A single origin of the coracomandibularis is the plesiomorphic state for chondrichthyans. There is a subsequent gain of the separate origin sate as a synapomorphy for [*Narke* + *Temera*] and an independent gain in Myliobatiformes, which is present in *Urolophus, Urobatis, Urotrygon, Plesiobatis, Hypanus, Potamotrygon, Neotrygon, Gymnura, Myliobatis, Aetobatus, Rhinoptera* and *Mobula*.

**MLtr (see discussion Maximum Likelihood tree):** Recovers a similar character reconstruction as the parsimony analysis. However, the more resolved topology of the Myliobatiformes also identifies the separate origin as a synapomorphy of clade 14.

69. **Coracohyomandibularis (if separate origins):** (0) Originates in the facia supporting the insertion of the coracoarcualis and on the pericardial membrane; (1) originates on the anterior portion of the ventral gill arch region and on the pericardial membrane.

**Ptr and MLtr (see discussion Maximum Likelihood and Parsimony trees):** A coracohyomandibularis that originates in the facia supporting the insertion of the coracoarcualis and on the pericardial membrane is a shared feature of *Narke* and *Temera.* The coracohyomandibularis originating on the anterior portion of the ventral gill arch region and the pericardial membrane is a shared feature of *Urolophus, Urobatis, Urotrygon, Plesiobatis, Hypanus, Potamotrygon, Neotrygon, Gymnura, Myliobatis, Aetobatus, Rhinoptera* and *Mobula,* and a synapomorphy of Myliobatiformes when excluding fossil taxa.

70. **Coracohyoideus:** (0) Present; (1) absent. Modified from Aschliman et al. [7] (char. 89). The character is separated into two different characters, 70 and 71, aiming to increase the grouping information.

**Ptr and MLtr (see discussion Maximum Likelihood and Parsimony trees):** The plesiomorphic state for chondrichthyans is the presence of a coracohyoideus. The lack of this muscle is a synapomorphy of clade 18 (Figures 1 and 2).

71. **Coracohyoideus (if present):** (0) Parallel to body axis; (1) runs parallel to the body axis and is very short; (2) runs diagonally from the wall of the first two gill slits to the posteromedial aspect of the basihyal or first basibranchial; (3) each muscle fuses with its antimere at a raphe near its insertion on the first hypobranchial.

**Ptr (see discussion Parsimony tree):** A coracohyoideus parallel to the body axis is recovered as the plesiomorphic condition for chondrichthyans, being present across several groups and taxa (*Chimaera, Harriotta, Chlamydoselachus, Hexanchus, Heterodontus, Squatina, Pristiphoridae, Squalus, Ginglymostoma, Raja, Bathyraja, Rhynchobatus, Glaucostegus, Rhina, Rhinobatos, Pseudobatos, Trygonorrhina* and *Zapteryx*). A very short coracohyoideus that runs parallel to the body axis is an autapomorphy of *Pristis.* A coracohyoideus running diagonally from the wall of the first two gill slits to the posteromedial aspect of the basihyal or first basibranchial is a synapomorphy of the clade 27, with the subsequent gain of the coracohyoideus fusing with its antimere at a raphe near its insertion on the first hypobranchial as a shared state for clade 16.

**MLtr (see discussion Maximum Likelihood tree):** Presents a similar reconstruction for this character as the parsimony tree. The diagonal arrangement of the coracohyoideus from the wall of the first two gill slits to the posteromedial aspect of the basihyal is not a synapomorphy due to the polytomic state of the thornbacks within clade 27.

#### 3.1.6 Synarcual and Axial Skeleton

48. **Cervicothoracic vertebrae:** (0) Unfused; (1) vertebral centra fused in a synarcual; (2) neural/basidorsal and hemal/basiventral elements fused. Modified from Aschliman et al. [7] (char. 5) to include Johanson et al.’s [102] observations.

**Ptr and MLtr (see discussion Maximum Likelihood and Parsimony trees):** There is uncertainty in reconstructing the basal state, as †*Doliodus,* symmoriids and hybodontiforms lack calcified vertebral centra. The presence of a “synarcual” formed by the fusion of the neural/basidorsal and hemal/basiventral elements is shared by the Holocephali. A synarcual characterized by the fusion of the cervicothoracic vertebral centra is a shared feature of batomorphs. All selachimorphs have unfused vertebral centra.

49. **Expanded basiventral process of cervical vertebrae:** (0) Absent; (1) present. Taken from Maisey et al. [63] (chars. 16–18).

**Figure 27:**
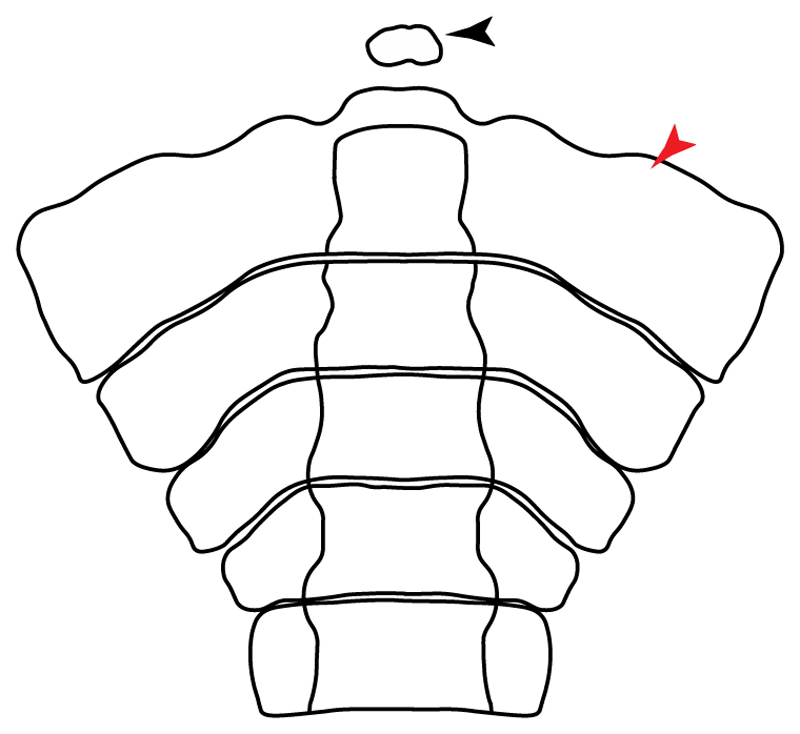
State (1). Interpretative drawing of the first cervical vertebrae of *Squatina punctata* in ventral view (ZMB 33878) redrawn and modified from Claeson and Hilger Text-Figure 2A in [103]. Black arrowhead: occipital hemicentrum; red arrowhead: basiventral process of cervical vertebra.

**Ptr (see discussion Parsimony tree):** The presence of expanded basiventral processes in their cervical vertebrae, in which the first process is larger than the subsequent ones, which become smaller continuously in size posteriorly (Figure 27), is a synapomorphy of clade 5.

50. **Occipital hemicentrum:** (0) Absent; (1) present. Modified from Shirai [35] (char. 21); and Klug [20] (char. 16). Based on observations by Claeson and Hilger [103] and Maisey et al. [83].

**Ptr and MLtr (see discussion Maximum Likelihood and Parsimony trees):** This character is a synapomorphy of the †*Pseudorhina* and *Squatina* clade (Figure 27).

51. **Lateral stays:** (0) Fused distally with medial crest; (1) free of medial crest (new). Taxa with no synarcual (i.e., outgroups) or with no lateral stays on the cervicothoracic synarcual (i.e., *Chimaera* and *Harriotta*) are coded as inapplicable (-), which makes the reconstruction of this character for basal chondrichthyans in the trees impossible.

**Figure 28:**
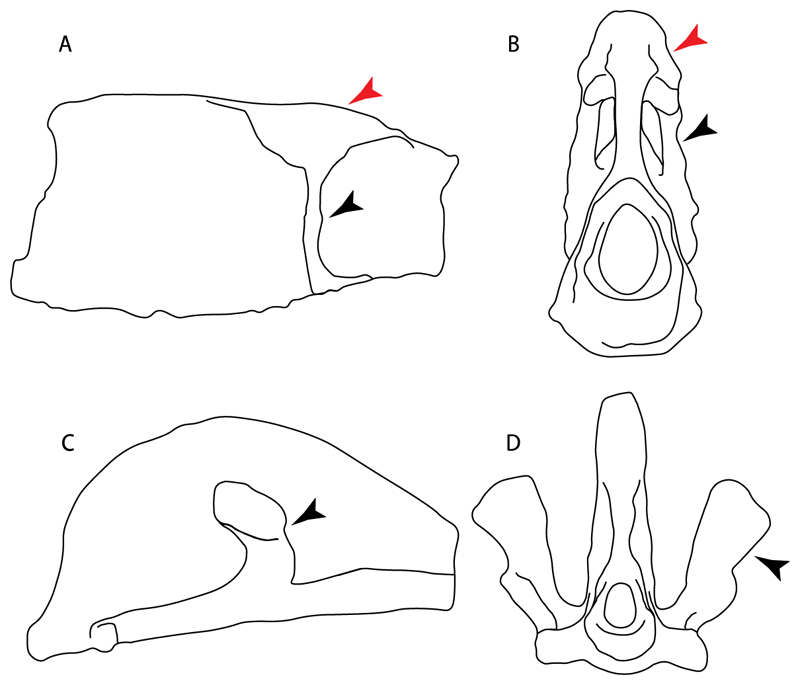
Interpretative drawings of lateral and frontal view of the synarcual. State (0): **(A,B)** *Mobula munkiana.* (SIO 85-34, https://sharksrays.org/ (accessed on 13 April 2020)). State (1): **(C,D)** *Rhina ancylostoma* (LACM 38117-38, https://sharksrays.org/ (accessed on 13 April 2020)). Arrowheads: lateral stays (black); medial crest (red).

**Ptr and MLtr (see discussion Maximum Likelihood and Parsimony trees):** The presence of lateral stays unfused with the medial crest of the synarcual is the plesiomorphic feature of batomorphs (Figure 28C,D), with the subsequent gain of the fused state (Figure 28A,B) as a synapomorphy of clade 17.

#### 3.1.7 Suprascapula and Pectoral Girdle

93. **Suprascapula:** (0) Absent; (1) fused medially; (2) unfused medially. Modified from Aschliman et al. [7] (char. 6). In some sharks, there seems to be an anterior portion of the scapular process that is detached from the scapula, referred to as suprascapular by Marramà et al. [59]. While this element is dorsal to the scapula, its interaction with other skeletal elements and its development seems to be different from that of the suprascapula of batomorphs.

**Figure 29:**
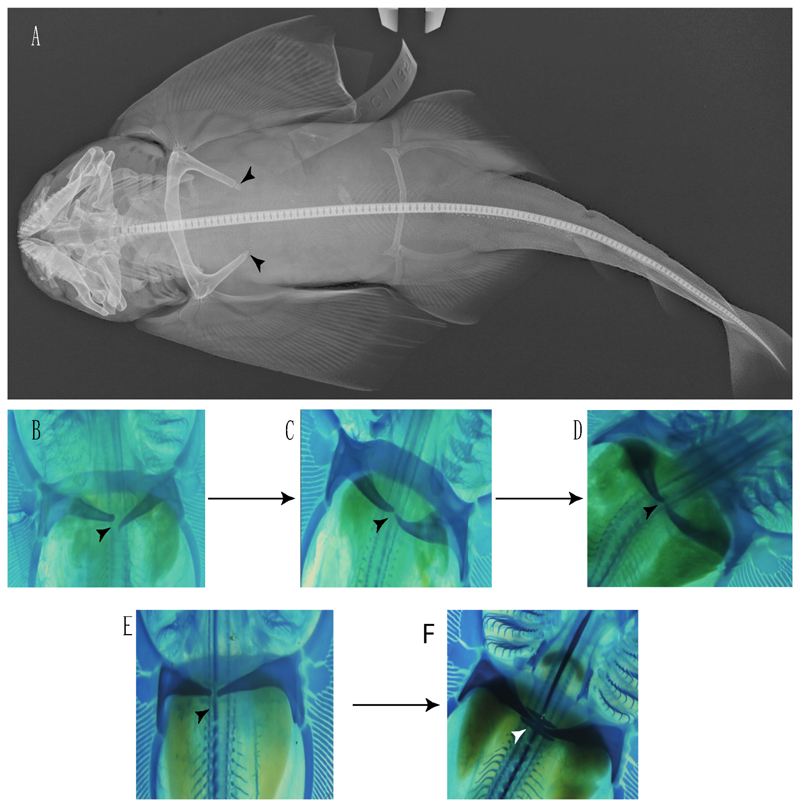
Dorsal view of the scapular region. State (0): **(A)** X-ray of a juvenile of *Squatina dumeril* [104] photo by Sandra J. Raredon. State (1): **(B-F)** Developmental stages of *Zapteryx brevirostris* (UREJ Unpublished data). Arrowheads: suprascapula cartilages.

**Ptr (see discussion Parsimony tree):** The lack of a suprascapula is the plesiomorphic state for chondrichthyans. The presence of a suprascapula (Figure 29A) (see also [8,9]) is a synapomorphy of clade 2, with an independent gain in *Squatina.* A medially developed suprascapula, as a single-element dorsal to the vertebral column connecting the scapulocoracoid antimeres (Figure 29B–F), is a synapomorphy for the batomorph crown group (clade 6).

**MLtr (see discussion Maximum Likelihood tree):** The basal placement of Rajiformes (rajoids and sclerorhynchoids) causes uncertainty for reconstructing the plesiomorphic state for the batomorph crown group, as the suprascapula is missing in most sclerorhynchoids. Consequently, this character is not recovered as a synapomorphy for the crown group, presenting independent gains in rajoids, †*Libanopristis* and clade 23.

94. **Suprascapula interaction with axial skeleton (if fused medially):** (0) Interacts with axial skeleton (articulated or fused); (1) free from axial skeleton (new). This character is proposed to include the variation observed in the suprascapula articulation in batomorphs.

**Figure 30:**
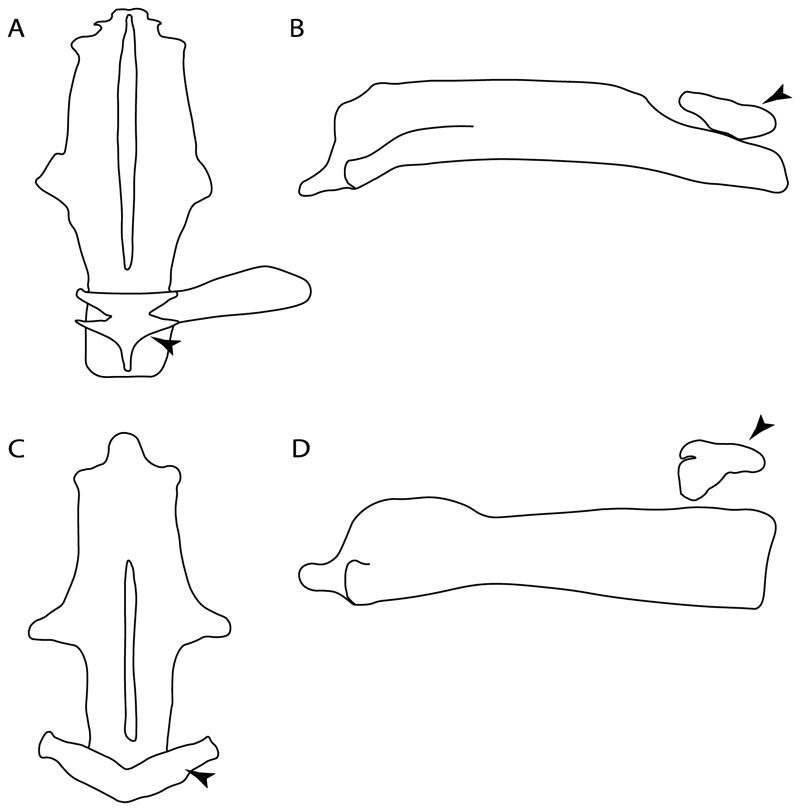
Dorsal and lateral view of synarcual and suprascapula. Interpretative drawing of: State (0): **(A, B)** *Trygonorrhina* sp. (uncatalogued) redrawn and modified from Claeson Text-Figure 5.16A in [71]. State (1): **(C,D)** *Narcine brasiliensis* (AMNH 77069, https://sharksrays.org/ (accessed on 13 April 2020)). Arrowheads: suprascapula.

**Ptr and MLtr (see discussion Maximum Likelihood and Parsimony trees) :** The presence of an interaction (i.e., fusion or articulation) between the suprascapula and the axial skeleton (Figure 30A,B) is the basal state for the batomorph crown group (Jurassic batomorphs such as †*Kimmerobatis*, †*Asterodermus*, †*Spathobatis* and †*Belemnobatis* lack the suprascapula—or at least, a calcified one). The absence of interaction between the suprascapula and the axial skeleton (Figure 30C,D) is a synapomorphy of clade 18.

95. **Suprascapula (if interacts with axial skeleton):** (0) Articulates with vertebral column; (1) fused medially to synarcual; (2) fused medially and laterally to synarcual (new). This character is proposed to account for the variation observed in the interaction between this suprascapula and axial skeleton in batomorphs.

**Figure 31:**
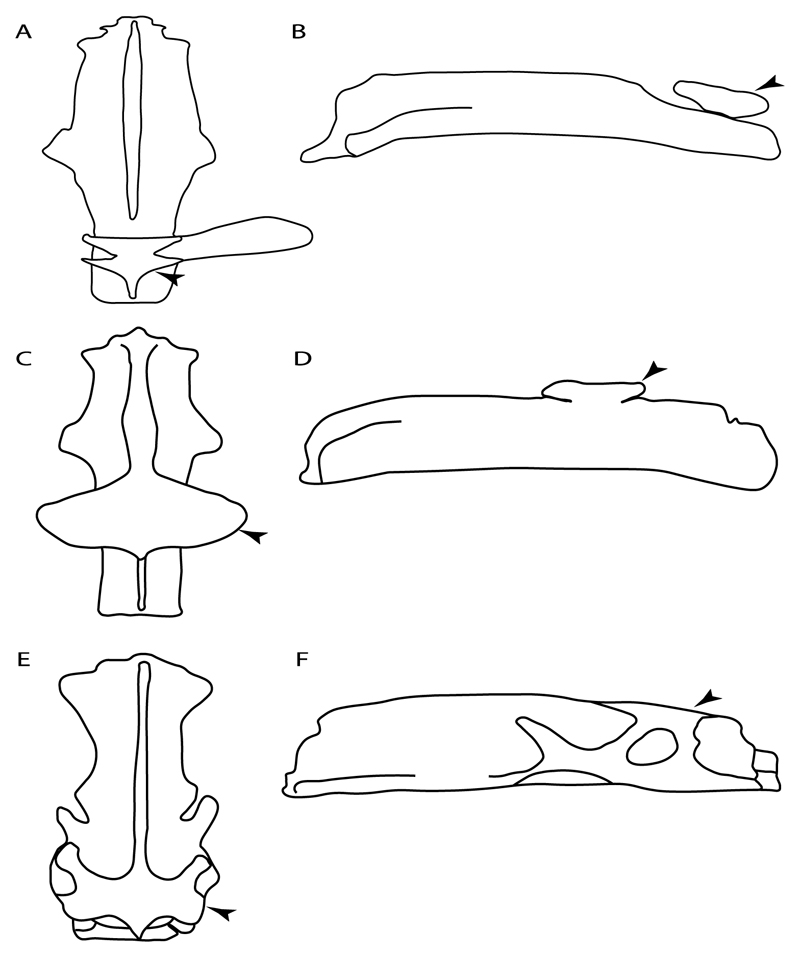
Dorsal and lateral view of synarcual and suprascapula. Interpretative drawing of: State (0): **(A,B)** *Trygonorrhina* sp. (Uncatalogued) redrawn and modified from Claeson Text-Figure 5.16A in [71]. State (1): **(C,D)** *Beringraja pulchra* redrawn and modified from Nishida Text-Figure 39C in [82]. State (2): **(E,F)** *Myliobatis tobijei* redrawn and modified from Nishida Text-Figure 38H in [82]. Arrowheads: suprascapula.

**Ptr (see discussion Parsimony tree):** The presence of a suprascapula that is fused medially to the synarcual (Figure 31C,D) is a synapomorphy of Rajiformes (inapplicable in *Asflapristis, Ptychotrygon* and *Sclerorhynchus*). The medial and lateral fusions of the suprascapula and synartual is a synapomorphy of Myliobatiformes (including *Zanobatus*).

**MLtr:** The presence of a suprascapula that is fused medially to the synarcual is not recovered as a synapomorphy of the Rajiformes as there is uncertainty regarding the basal state, being inapplicable for the Jurassic batoids and *Asflapristis, Ptychotrygon* and *Sclerorhynchus,* as this cartilage is missing. The medial and lateral fusion of the suprascapula and synarcual is a synapomorphy of Myliobatiformes.

96. **Suprascapula-scapula articulation:** (0) Curved; (1) crenate; (2) ball and socket; (3) straight. Modified from Aschliman et al. [7] (char. 53) to include the variation observed in the articulation between the suprascapula and scapula in batomorphs.

**Figure 32:**
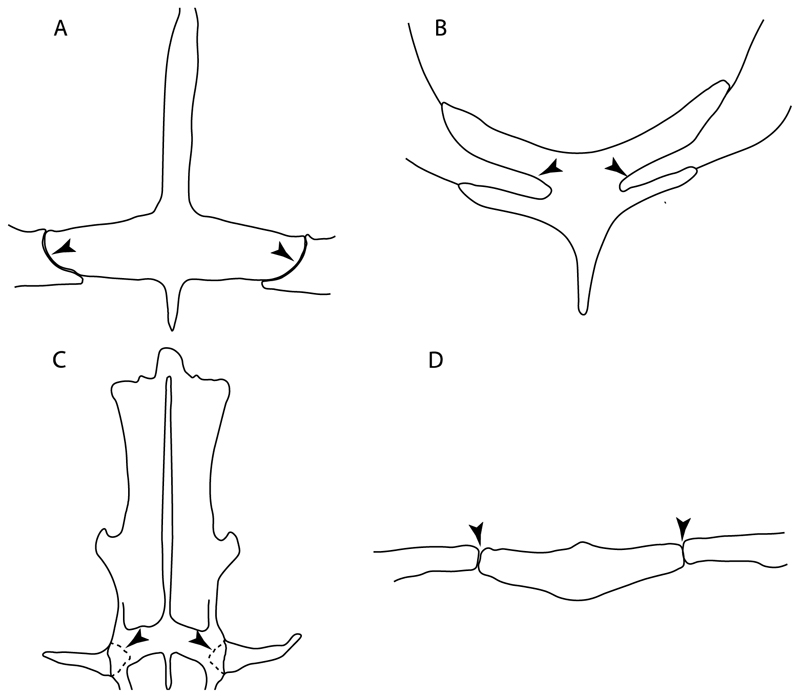
Interpretative drawing of synarcual and suprascapula in dorsal view. State (0) **(A)** *Raja clavata* (NHMUK 1963.5.14.34-36). State (1): **(B)** *Pseudobatos percellens.* (UERJ 1240). State (2): **(C)** *Urolophus aurantiacus* (AMNH 258305, https://sharksrays.org/ (accessed on 23 March 2020)). State (3): **(D)** *Torpedo* sp. (NHMUK 72261). Arrowheads: articulation between scapula and suprascapula.

**Ptr (see discussion Parsimony tree):** A crenated articulation between the scapula and suprascapula (Figure 32A) is the commonest feature of the batomorph crown group, being present in Torpediniformes (*Platyrhina*, †*Eoplatyrhina, Platyrhinoidis*, †*Tingitanius*) and Rhinopristiformes (†“Rhinobatos” *maronita,* † “*R*.” *latus*, †*Stahlraja*, †*Tlalocbatus, Pristis, Rhynchobatus, Glaucostegus,* Rhina, *Rhinobatos, Pseudobatos*, †*Eorhinobatos*, †*Pseudorhinobatos, Trygonrrhina, Zapteryx, Aptychotrema* and †*Iansan*). A straight articulation surface (Figure 32B) is a synapomorphy of clade 18. A curved articulation surface (Figure 32C) is a synapomorphy of Rajiformes (inapplicable in *Asflapristis, Ptychotrygon* and *Sclerorhynchus),* whereas a ball-and-socket articulation (Figure 32D) is a synapomorphy of Myliobatiformes.

**MLtr (see discussion Maximum Likelihood tree):** There is uncertainly regarding the basal state for the batomorph crown group, as the placement of Rajiformes at the base of this group creates conflict between curved and crenate states. For the reaming batomorphs, both topologies recovered similar character reconstructions, with the crenated articulation as the basal state for the batomorph crown group, whereas the straight and ball-and-socket articulations are synapomorphies for electric rays and Myliobatiformes, respectively.

97. **Crenated suprascapula (variations):** (0) With lateral projections; (1) thin upper and lower lobes; (2) upper lobe wider than lower; (3) of similar size and width (new). This character is proposed to include for the variation observed in the suprascapula of Platyrhinidae and Rhinopristiformes.

**Figure 33:**
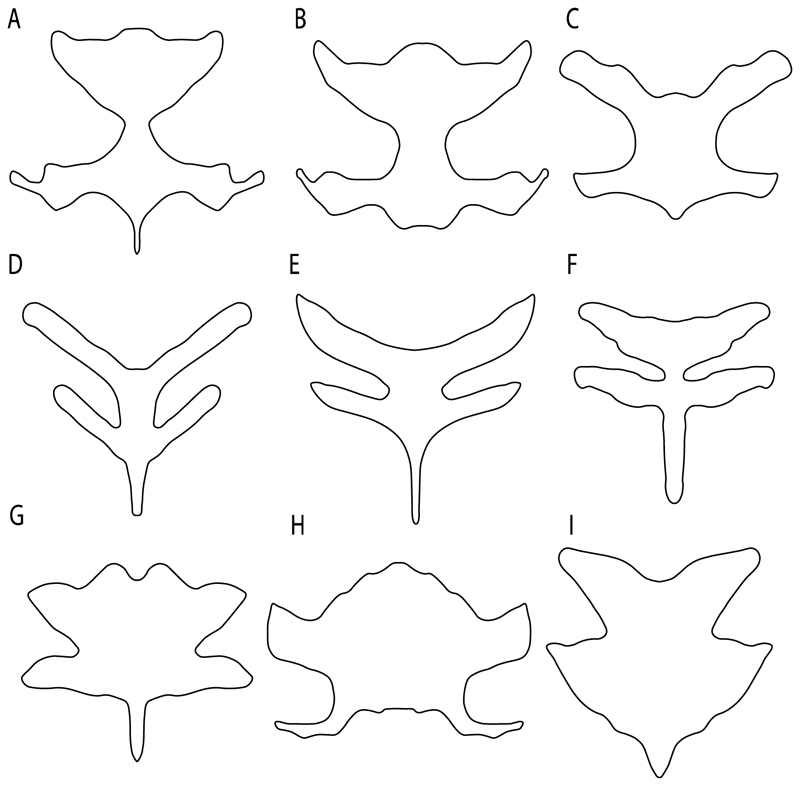
Interpretative drawings of suprascapula in dorsal view. State (0): **(A)** *Platyrhina sinensis* (MNHN 1307); **(B)** *Platyrhinoidis triseriata* (MNHN 3211). State (1): **((C)** *Aptychotrema vincentiana* (CSIRO 101, https://sharksrays.org/ (accessed on 23 March 2020)); **(D)** *Glaucostegus* sp. (**NHMUK**1967-21-13); **(E)** *Pseudobatos percellens* (UERJ 1240). State (2): **(F)** *Rhynchobatus springeri* (https://sharksrays.org/ (accessed on 23 March 2020)); **(G)** *Rhina ancylostoma* (LACM 38117-38, https://sharksrays.org/ (accessed on 23 March 2020)); **(H)** *Pristis zijsron* (ANSP 101398) redrawn and modified from da Silva and de Carvalho Text-Figure 19 in [22]. State (3): **(I)** *Zapteryx exasperata* (CNPE-IBUNAM 20528).

**Ptr and MLtr (sae dissussion Maximum Likelihood and Parsimony trees):** A suprascapula with a narrow and larger upper lobe (Figure 33C-E) is the basal state for Rhinopristiformes and is present in *Rhynchobatus, Glaucostegus, Rhinobatos, Pseudobatos, Aptychotrema* and †*Stahlraja*. Within Rhinopristiformes, a suprascapula with an upper lobe that is wider than the lower lobe (Figure 33F–H) is a synapomorphy of clade 13, while the presence of a suprascapula with both lobes being of similar size (Figure 33I) is a synapomorphy of [*Zapteryx* + *Trygonorrhina*].

**Figure 34:**
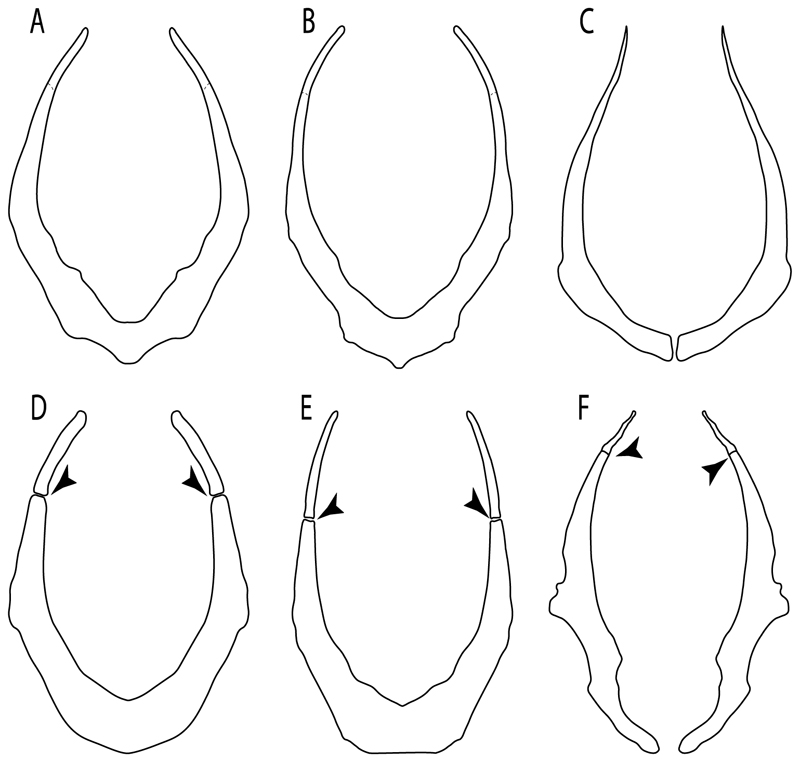
Interpretative drawings of the pectoral girdle in frontal view. State (0): **(A)** *Squalus acanthias* (HUMZ 30291) redrawn and modified from da Silva and de Carvalho Text-Figure 33C in [8]; (B) *Squalus megalops* (MZUSP 110973) redrawn and modified from da Silva and de Carvalho Text-Figure 25D in [8]; **(C)** *Hexanchus griseus* (CAS uncatalogued) redrawn and modified from da Silva and de Carvalho Text-Figure 32E in [8], State (1): **(D)** *Squalus brevirostris* (AMNH 258171, https://sharksrays.org/ (accessed on 23 March 2020)); **(E)** *Squalus mitsukurii* (https://sharksrays.org/ (accessed on 23 March 2020)); **(F)** *Chlamydoselachus anguineus* (MZUSP 110974) redrawn and modified from da Silva and de Carvalho Text-Figure 26E in [8]. Arrowheads: articular surface between the scapula and the scapular process.

**Ptr (see discussion Parsimony tree):** The presence of lateral projections on the lower lobe of the suprascapula (Figure 34A,B) is a shared feature of *Platyrhina* and *Platyrhinoidis.* We could not determine the state in fossil thornbacks (†*Tethybatis,* †*Tingitanius* and †*Eoplatyrhina*), resulting in an uncertainty in the state reconstructions for the corresponding clade.

**MLtr (see discussion Maximum Likelihood tree):** The presence of a suprascapula with thin upper and lower lobes is the basal state of clade 23. There is a subsequent independent gain in the lateral projections for clade 27 caused by the paraphyletic condition in extant thornbacks (*Platyrhina* and *Platyrhinoidis*).

98. **Scapular process-scapula:** (0) Fused; (1) articulated (new). The interaction between the scapula and scapular process is a rather variable within sharks.

**Ptr and MLtr (see discussion Maximum Likelihood and Parsimony trees):** The fusion between the scapular process and the scapula is the basal condition for chondrichthyans. Members of *Squalus* display variation with this species *Squalus acanthias* and *S. megalops* having a fused process (Figure 34A,B), while *S. mitsukurii* and *S. brevirostris* present an articulation between the process and the scapula (Figure 34D,E). There is also variation on the state in Hexanchiformes, wiih *Hexanchus griseus* presenting the fused state (Figure 34C) and *Chlamydoselachus anguineus* presenting the articulated state (Figure 34F). Within sharks, state (1) is a shared feature between *Heterodontus* and *Chlamydoselachus.*

99. **Scapular process:** (0) Short and dorsally directed; (1) long, U-curved and posteriorly directed; (2) short and posterodorsally directed. Modified from Aschliman et al. [7] (char. 56). The short, posterodorsally directed state was included to account for the variation observed in *Pseudobatos.*

**Figure 35:**
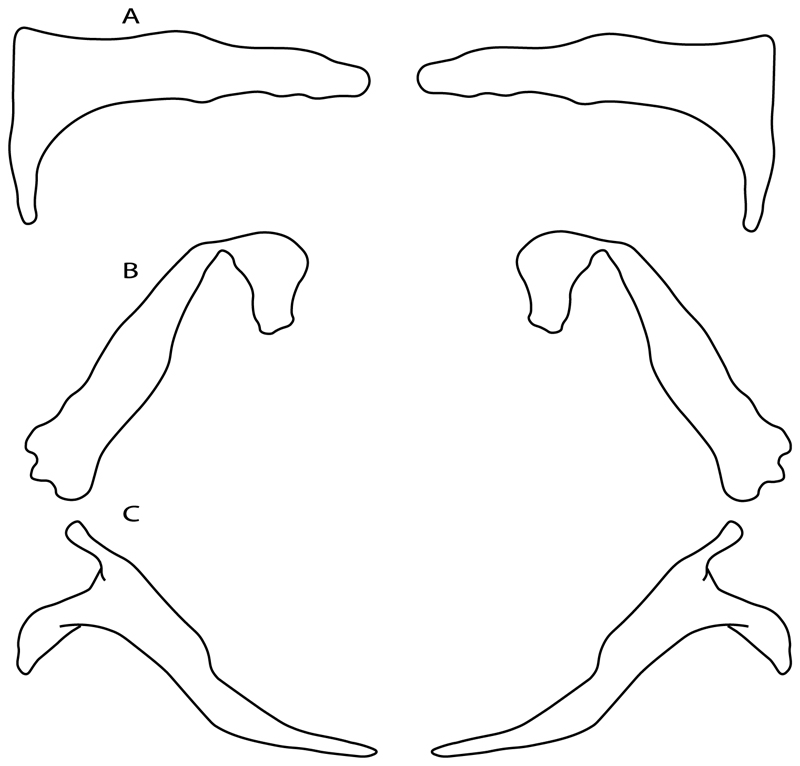
Interpretative drawings of the dorsal portion of the scapula. State (0): **(A)** *Zapteryx exasperata* (CNPE–IBUNAM 20528). State (1): **(B)** *Narcine bancroftii* (CAS 18246). State (2): **(C)** *Pseudobatos percellens* (UERJ 1240).

**Ptr and MLtr (see discussion Maximum Likelihood and Parsimony trees):** The presence of a short and dorsally directed scapular process is the basal state for chondrichthyans (Figure 35A). The presence of a long, U-shaped and posterodorsally directed scapular process (Figure 35B) is a synapomorphy of clade 18. The presence of a short, posterodorsally directed scapular process (Figure 35C) is an autapomorphy of *Pseudobatos*.

101. **Scapulocoracoid/pterygia articulation:** (0) Facets; (1) facets/condyles; (2) condyles. Modified from de Carvalho [38] (char. 38), based on observations by da Silva and de Carvalho [8].

**Figure 36:**
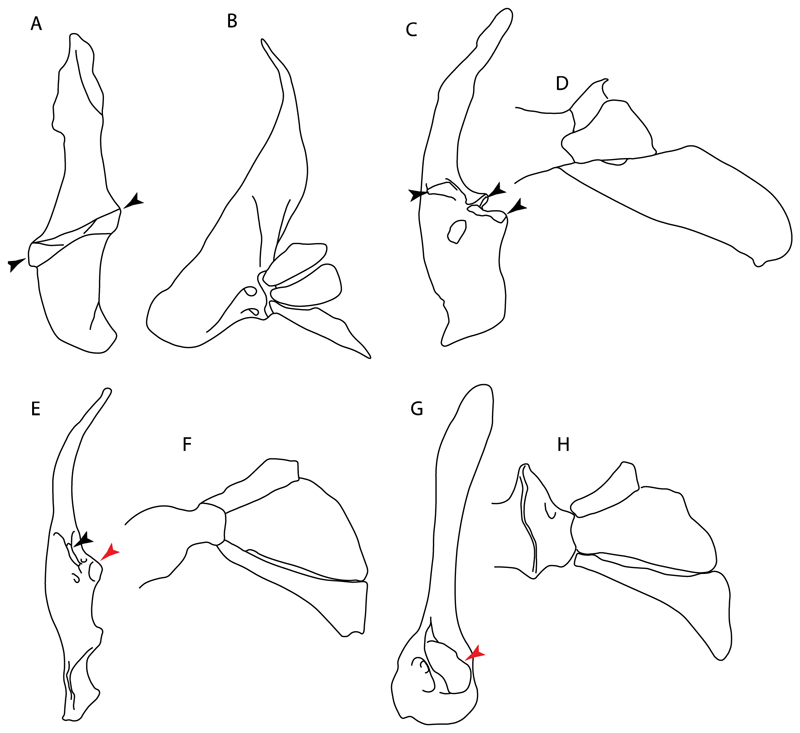
Interpretative drawings of the lateral face of the scapulacoracoid and the pectoral-fin supports in dorsal view. State (0): **(A,B)** †*Tribodus limae* (AMNH FF 13958) redrawn and modified from Lane and Maisey Text-Figures 5B and 9B in [105]; **(C,D)** *Mustelus canis* (ANSP 178683) redrawn and modified from da Silva and de Carvalho Text-Figure 30C,F in [8]. State (1): **(E,F)** *Squalus megalops* (MZUSP 110973) redrawn and modified from da Silva and de Carvalho Text-Figure 25C,G in [8]. State (2): **(G,H)** *Heterodontus francisci* (MZUSP 112022) redrawn and modified from da Silva and de Carvalho Text-Figure 1D,H in [8]).

**Ptr (see discussion Parsimony tree):** There is uncertainty regarding the basal-state reconstruction for chondrichthyans as the character is unknown in †*Doliodus*, †*Ozarcus*, †*Cobelodus, Chimaera* and *Harriotta.* The presence of an articulation between the scapulocoracoid and pectoral elements composed by facets (Figure 36A–D) is a synapomorphy of *Scyliorhinus* and *Mustelus,* with independent gains in †*Hybodus* and †*Tribodus.* The combination of facets and condyles in the articulation between the pterygia and the scapulocoracoid (Figure 36F,G) is a synapomorphy of clade 4 (with an independent gain of the facet in *Hexanchus.* Within Elasmobranchii, the basal state is the presence of condyles as the means of articulation of the pectoral elements (Figure 36I,J), present in selachimorphs (*Heterodontus, Squatina, Pristiophorus, Ginglymostoma, Hemiscyllium*) and batomorphs.

**MLtr (see discussion Maximum Likelihood tree):** The presence of facets for the articulation of the proximal pectoral elements is the basal condition for euselachians. The combination of facets and condyles for the articulation of the pterygia is a synapomorphy of clade 4, with the subsequent independent gains of the full condyle articulation as a synapomorphy in clades 2 and 5, and in batomorphs.

102. **Condyles:** (0) Single condyle; (1) pro + mesocondyle; (2) meso + metacondyle; (3) three separated condyles. Modified from de Carvalho [38] (char. 38), based on observations by da Silva and de Carvalho [8].

**Figure 37:**
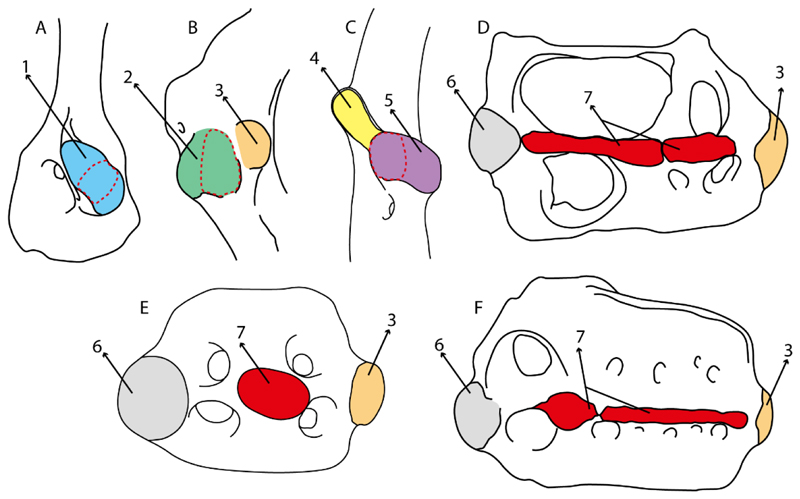
Interpretative drawings of scapulacoracoid in lateral view. State (0): (**A**) *Heterodontus francisci* (MZUSP 112022) redrawn and modified from da Silva and de Carvalho 8; Text-Figure 1A in [8], State (1): **(B)** *Squatina guggenheim* (MZUSP 1108171) redrawn and modified from da Silva and de Carvalho Text-Figure 12C in [8]. State (2): **(C)** *Chlamydoselachus anguineus* (MZUSP 110974) redrawn and modified from da Silva and de Carvalho Text-Figure 26A in [8]. State (3): **(D)** *Gymnura japonica* (HUMZ 48301) redrawn and modified from Nishida Text-Figure 31A in [82]; **(E)** *Rhinobatos horkelii* (MZUSP uncatalogued) redrawn and modified from da Silva and de Carvalho Text-Figure 18A in [8]; **(F)** *Zapteryx brevirostris* (UERJ-PMB 35). Annotations: (1) Single condyle (blue); (2) pro + mesocondyle (green); (3) metacondyle (orange); (4) facet for propterygium (yellow); (5) meso + metacondyle (purple); (6) procondyle (gray); (7) mesocondyle (red).

**Ptr (see discussion Parcimony tree):** The presence of a single condyle (Figure 37A) is a shared feature between *Heterodontus* and *Hemiscyllium.* The presence of two condyles, for the articulation of the pro + mesopterygium and the metapterygium, respectively, is a shared feature of *Squatina* and *Ginglymostoma* (Figure 37B). The presence of a single condyle for the articulation of the meso + metaptorygium and a facet for the propterygium (Figure 37C) is a synapomorphy for clade 4 (this is not recovered in the ML tree). The presence of three separated condyles (Figure 37D–F) is the basal feature for [Selachimorpha + Batomorpha] being present in *Pristiophorus* and all batomorphs and is additionally a synapomorphy as an independent gain of clade 4.

**MLtr (see discussion Maximum Likelihood tree):** The presence of three separated condyles is recovered as the basal state for batomorphs with an independent gain in *Pristiophorus.*

103. **Mesocondyle:** (0) Single and small; (1) segmented and small; (2) forming an elongated ridge. Modified from Aschliman et al. [7] (char. 59). This character is proposed to include the variation observed in the mesocondyle of the taxa with three condyles (i.e., separated condyles).

**Figure 38:**
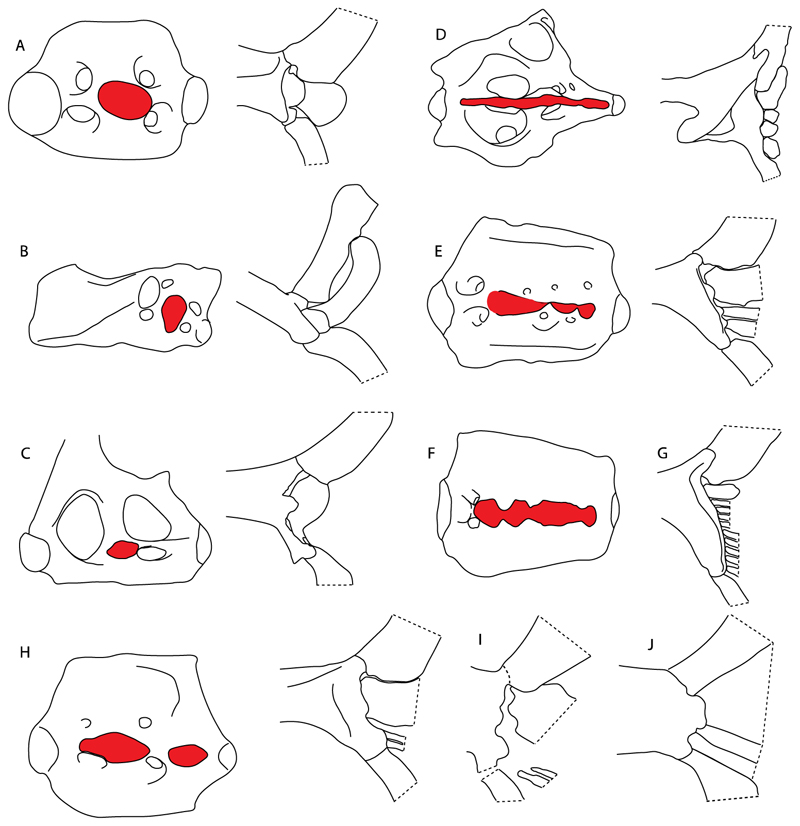
Interpretative drawings of scapulacoracoid in lateral view and dorsal view of the scapulacoracoid and pectoral elements. State (0): **(A)** *Rhinobatos productus* (CNPE-IBUNAM 17829); **(B)** *Narke japonica.* (HUMZ 94970) redrawn and modified from Nishida Text-Figure 32E in [82]; **(C)** *Raja clavata* (BRC–Raja). State (2): **(D)** *Myliobatis goodei* (HUMZ 91851) redrawn and modified from Nishida Text-Figure 31C [82]; **(E)** *Zapteryx exasperata* (CNPE-IBUNAM 20528); **(F)** *Zanobatus schoenleinii* (UF 176858, https://sharksrays.org/ (accessed on 25 March 2020)); **(G)** *Zanobatus* sp. (MNHN 1989. 12.91) redrawn and modified from Brito and Seret Text-Figure 5b in [106]. State (1): **(H)** *Aptychotrema vincentiana* (CSIRO 101, https://sharksrays.org/ (accessed on 25 March 2020)); **(I)** †*Stahlraja sertanensis* (UERJ-PMB P 400); **(J)** †*Tlalocbatos applegatei* (IGM 5853). Mesocondyle marked in red color.

**Ptr (see discussion Parsimony tree):** A single, small and rounded mesocondyle (Figure 38A–C) is the basal state for batomorphs being present in texa of all major groups: Rajiformes (*Raja, Bathyraja*, †*Ostarriraja*, †*Cyclobatis,* †*Ptychotrygon*, †*Sclerorhynchus*, †*Libanopristis* and †*Asflapristis*); Torpediniformes (†*Titanonarke, Torpedo, Hypnos, Narcine, Narke, Temera*, *Platyrhina, †Eoplatyrhina, Platyrhinoidis*, †*Tingitanius*); Rhinopristiformes (†“*Rhinobatos” maronita,* †“*R*.” *latus, Pristis, Rhynchobatus, Glaucostegus, Rhina, Rhinobatos, Pseudobatos*, †*Britobatos*, †*Iansan* and †*Rhombopterygia*); Myliobatiformes (*Urolophus, Urotatis, Urotrygon, Hexatrygon, Plesiobatis*, *Hypanus, Potamotrygon*) and Jurassic ba-omorphs †*Kimmerobatis*, †*Aslerodermus,* †*Spathobatis*, †*Belemnobatis*). An elongated mesocondyle forming a ridge for articulation of the pectoral radials (Figure 38D–G) is recovered as a shared feature between *Trygonorrhina* and *Zapteryx Zanobatus, Plesiozanobatus, Hypanus, Neotrygon, Gymnura, Myliobatis, Aetobatus, Rhinoptera* and *Mobula*. A segmented mesocondyle that is split in two elements (Figure 38H–J1 with the posterior one serving as articulation for a group of pectoral radials anteriorly to the metacondyle represents a shared feature (possibly an independent gain) of †*Tethybatis*, †*Stahlraja*, †*Tlalocbatos* and *Aptychotrema.*

**MLtr (see discussion Maximum Likelihood tree):** This analysis provides a similar basal-node reconstruction for this character as found in the parsimony analysis. The separation of Trygonorrhinidae from the main Rhinopristiformes clade places the elongated mesocondyle, forming a sort of ridge for the articulation of the pectoral radials as an independent gain and synapomorphy for [*Trygonorrhina* + *Zapteryx*] and the [*Zanobatus* + *Plesiozanobatus*], with additional independent gains in clade 32.

#### 3.1.8 Pelvic Girdle and Claspers

117. **Lateral prepelvic processes:** (0) Absent; (1) present. The modification of this character from the multistate coding used in McEachran and Dunn [107] (char. 36) is because the three proposed states (i.e., short to moderately long; extremely long with acute tips; and extremely long with biramous tips) are difficult to interpret in fossil specimens. Consequently, binary coding (presence/absence) is used here.

**Ptr and MLtr (see discussion Maximum Likelihood and Parsimony trees):** The absence of lateral prepelvic processes is the plesiomorphic condition for chondrichthyans. The presence of these lateral processes, conversely, can be considered as an independent gain and a synapomorphy of clades 7 and 18.

118. **Poetpelvic processes:** (0) Absent; (1) present. Modified from Claeson et al. [23] (char. 37). Initially observed in *Platyrhina* and *Platyrhinoidis* only by de Carvalho [67], the coding of this character was changed for *Pseudobatos* and *Torpedo* based on observations by da Silva et al. [10], as these two taxa also show postpelvic processes.

**Ptr and MLtr (see discussion Maximum Likelihood and Parsimony trees):** The lack of postpelvic processes is the plesiomorphic state for chondrichthyans.

**Figure 39:**
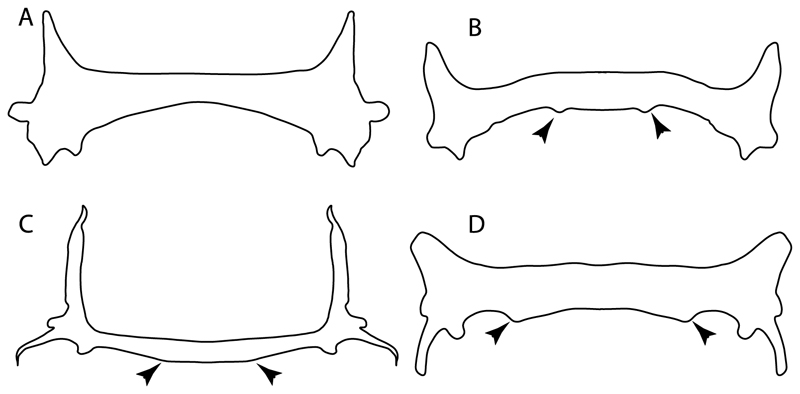
Pelvic girdle of selected taxa in dorsal view. State (0): **(A)** *Bathyraja leucomelanos* (MNHN 2005-2740) from Iglesias and Hartmann Text-Figure 13 in [84]. State (1): **(B)** *Pseudobatos percellens* (AMNH 8913); **(C)** *Narcine bancroftii* (CAS 18246); **(D)** *Platyrhina sinensis* (MNHN 1307). Arrowheads: postpelvic process.

**Ptr (see discussion Parsimony tree):** The presence of these processes (Figure 39B–D) is a synapomorphy of Torpediniformes and Jurassic batomorphs, with additional independent gains in *Hemiscyllium* and Rhinopristiformes, being present in †*Tlalocbatus, Pseudobatos, Rhinobatos, Glaucostegus, Zapteryx* and *Aptychotrema.*

**MLtr (see discussion Maximum Likelihood tree):** The presence of these processes is a synapomorphy of batomorphs, with a subsequent loss in the non-Jurassic forms. Additional independent gains of the postpelvic processes are recovered as synapomorphirs of clades 22 and 24, with the subsequent loss of these processes as a synapomorphy in [*Temera* + *Narke*] and in Myliobatiformes.

119. **Posterior margin of puboischiadic bar:** (0) Straight or bending anteriorly; (1) convex (new).

**Figure 40:**
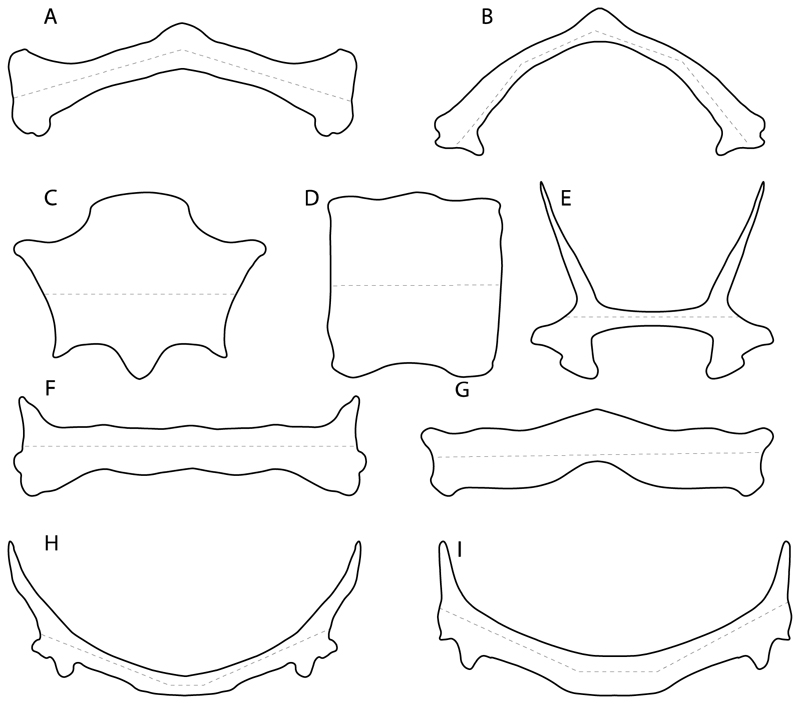
Dorsal view of pelvic girdle. **(A)** *Pristis zijsron* (ANSP 101398), redrawn and modified from da Silva et al. Text-Figure 2A [10]; **(B)** *Rhina ancylostoma* (CAS 56636) redrawn and modified from da Silva et al. Text-Figure 2B in [10]; **(C)** *Hexanchus nakamurai* (UF 165855 https://sharksrays.org/ (accessed on 25 March 2020)); **(D)** *Chlamydoselachus anguineus* (UF 44302 https://sharksrays.org/ (accessed on 25 March 2020)); **(E)** †*Cyclobatis oligodactylus* (NHMUK PV P 601); **(F)** *Zapteryx brevirostris* (UREJ unpublished data); **(G)** †*Asterodermus platypterus* (JM-SOS-3647). State (1): **(H)** *Torpedo ocellata* (AMNH 4128) redrawn and modified from da Silva et al. Text-Figure 3A in [10]; **(I)** *Squatina nebulosa* (AMNH 258172, https://sharksrays.org/ (accessed on 25 March 2020)). Dashed line highlights the direction of the posterior margin of the pelvic girdle.

**Ptr and MLtr (see discussion Maximum Likelihood and Parsimony trees):** The presence of an anterior margin, roughly straight or bending anteriorly (Figure 40A–G), is the basal state for the chondrichthyan tree. The presence of a puboischiadic bar bending towards the tail (Figure 40H,I) is a synapomorphy of [*Squatina* + †*Pseudorhina*] and clade 18.

120. **Anterior margin of puboischiadic bar (if posterior margin straight or concave):** (0) Straight; (1) anteriorly arched (new). This character is proposed to group the different patterns of the anterior margin of puboischiadic bar in taxa with a straight or anteriorly bending posterior margin.

**Figure 41:**
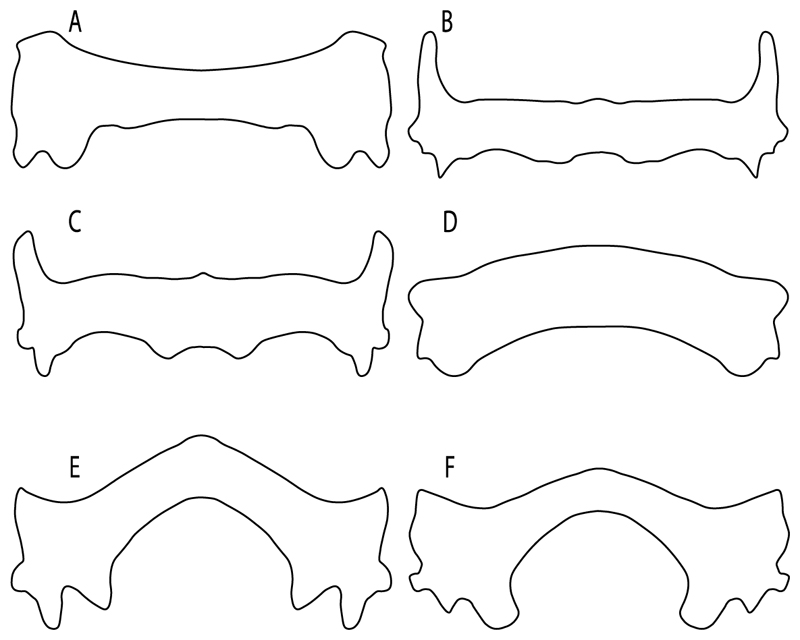
Dorsal view of pelvic girdle. State (0): **(A)** *Hemiscyllium ocellatum* (AMNH 44128, https://sharksrays.org/ (accessed on 25 March 2020)); **(B)** *Rhinobatos productus* (CNPE-IBUNAM 17829); **(C)** *Glaucostegus granulatus* (NHMUK 1926.5.26.5). State (1): **(D)** *Heterodontus francisci* (AMNH 96795, https://sharksrays.org/ (accessed on 25 March 2020)); **(E)** *Zanobatus schoenleinii* (MNHN N/C) redrawn and modified from da Silva et al. Text-Figure 4A in [24]; **(F)** *Urobatis halleri* (CAS 17327) da Silva et al. Text-Figure 8B in [10].

**Ptr and MLtr (see discussion Maximum Likelihood and Parsimony trees):** The presence of a straight anterior margin of puboischiadic bar is the basal state for the chondrichthyans (Figure 41A–C). The arching of the anterior margin of the puboischiadic bar (Figure 41D–F) is a synapomorphy of clade 12, with independent gains in Myliobatiformes, *Heterodontus* and *Mustelus*.

**Figure 42:**
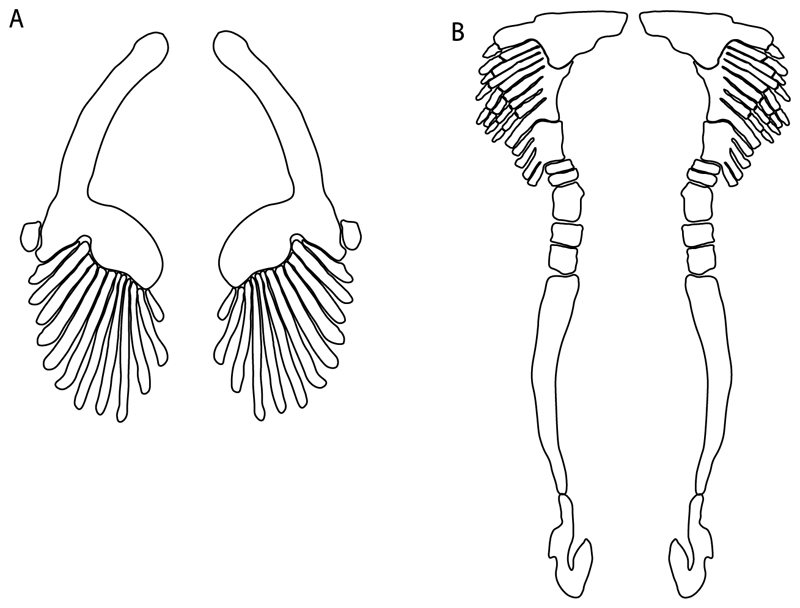
Dorsal view of pelvic girdle. State (0): **(A)** *Chimaera cubana* (USNM 400700, https://sharksrays.org/ (accessed on 25 March 2020)); **(B)** Reconstruction of *Hybodus hauffianus* (SMNS 10050).

124. **Pelvic girdle:** (0) Separated; (1) fused. Modified from Maisey [13] (char, 37), based on observations by Klug et al. [20] (Figure 2), Stumpf et al. [108] and Coates et al. [109]. Current fossil evidence suggests that the separation of two halves, or at least a not very well-mineralized mid-bar of the pelvic girdle, is the basal state across hybodontiform-like sharks (Figure 42A,B) (SMNS 10062) (NHMUK PV P 339).

**Ptr and MLtr (see discussion Maximum Likelihood and Parsimony trees):** The presence of two pelvic halves is a shared feature of holocephalians and hybodonts. A fused pelvic girdle, conversely, is a shared feature of elasmobranchs, and due to the uncertainty at the base of the tree recovered, it does not represent a synapomorphy for Elasmobranchii in the current trees.

#### 3.1.9 Paired Fins

91. **Radial calcification:** (0) Crustal; (1) catenated. Taken from Marramä et al. [33] (char. 104).

**Ptr (see discussion Parsimony tree):** The crustal calcification is the basal feature for chondrichthyans. The presence of catenated calcification is a synapomorphy of rajoids, with an independent gain in some stingrays, being present in †*Asterotrygon,* †*Heliobatis, Urolophus, Urobatis, Hypanus, Neotrygon*, †*Lessiniabatis*, †*Arechia* and †*Tethytrygon.* The genera *Potamotrygon* and *Urotrygon* present variations of the type of radial calcification depending on the portions of the pectoral fins with basal radials: those closer to the propterygium, mesopterygium and metapterygium present crustal calcification, while the subsequent series display catenated calcification.

**MLtr (see discussion Maximum Likelihood tree):** Presents a similar reconstruction to the parsimony analysis. The catenated calcification is recovered as an independent gain and a synapomorphy of clades 7 and 30.

92. **Radial calcification (if catenated):** (0) Two chains; (1) four chains (new). This character includes the remaining variation observed by Schaefer and Summers [63] regarding the number of chains.

**Ptr and MLtr (see discussion Maximum Likelihood and Parsimony trees):** The presence of two chains in the pectoral radials is a shared feature of clade 7, while the presence of four-chained pectoral radials is a shared feature of clades 14 and 29.

#### 3.1.10 Pectoral Fins

107. **Propterygium extending anteriorly:** (0) Absent; (1) present. Modified from Aschliman et al. [7] (char. 62), based on de Carvalho and Maisey [15] (char. 65). The original character was proposed as a synapomorphy for platyrhinids or as a shared feature between platyrhinids and *Zanobatus,* which according to de Carvalho [66] also present the following condition: extension of the propterygium and its associated radials to the anterior margin of the disc on both sides of the snout and rostrum. Aschliman et al. [7] suggested that the extension of the propterygium observed in platyrhinids and *Zanobatus* is similar to the condition present in Myliobatiformes and *Bathyraja.* However, in pelagic stingrays (e.g., *Myliobatis Aetobatus, Rhinoptera* and *Mobula),* the head stands out of the pectoral disc, causing modifications to the neurocranium and pectoral disc, suggesting differences in this structure. In contrast, the condition of Rajiformes resembles that of the remaining batomorphs.

Furthermore, homology issues arise in groups in which the propterygium does not reach the anterior margin of the disc (e.g., Torpediniformes and Rhinopristiformes). These groups present different conditions affecting the extension of their propterygium. There are considerable modifications in the cephalic and anterior portions of the body in electric rays due to the branchial electric organs. In Rhinopristiformes, there is a significant development of the rostral cartilages. Whatever is the case, there seem to be different conditions in batomorphs affecting the extension propterygium. Consequently, we only coded the presence or absence of the anterior extension of the propterygium.

**Figure 43:**
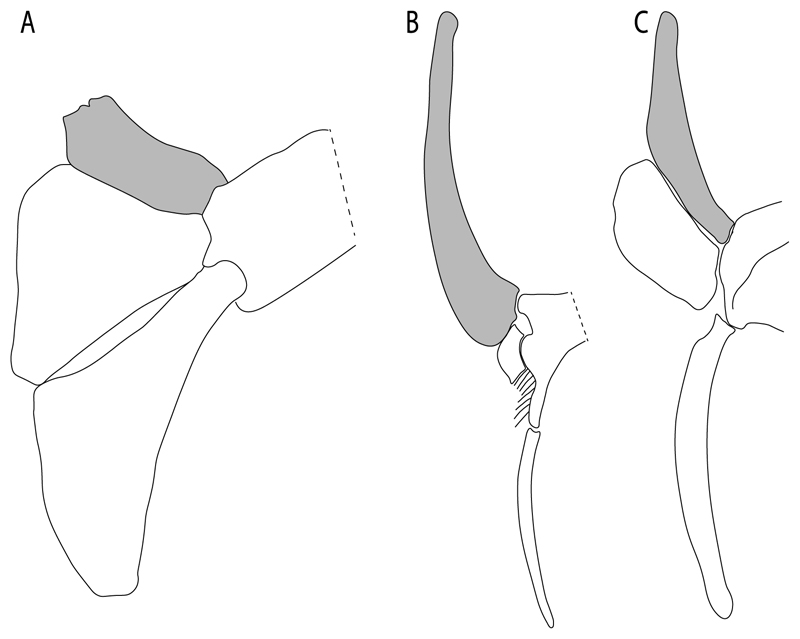
Interpretative drawings of the basal pectoral elements in dorsal view. State (0) **(A)** *Squatina guggenheim* (MZUSP 110871) redrawn and modified from da Silva and de Carvalho Text-Figure 12G in [8]. State (1) **(B)** *Glaucostegus granulatus* (NHMUK 2012.2.8.54); (C) †*Asterodermus platypterus* (NHMUK PV P 10934). Propterygium colored in gray.

**Ptr and MLtr (see discussion Maximum Likelihood and Parsimony trees):** The lack of an anteriorly elongated propterygium is the basal state for chondrichthyans, but the presence of an anterior elongated propterygium is a synapomorphy for the batomorphs (Figure 43B,C).

108. **First segment of propterygium (if propterygium extends anteriorly):** (0) Not reaching the nasal capsules; (1) reaches the level of nasal capsules; (2) extending well beyond the nasal capsules. This character recovers the variation of the placement of the first propterygial segment with respect to the nasal capsules of Aschliman et al. [7] (char. 62 and 63).

**Figure 44:**
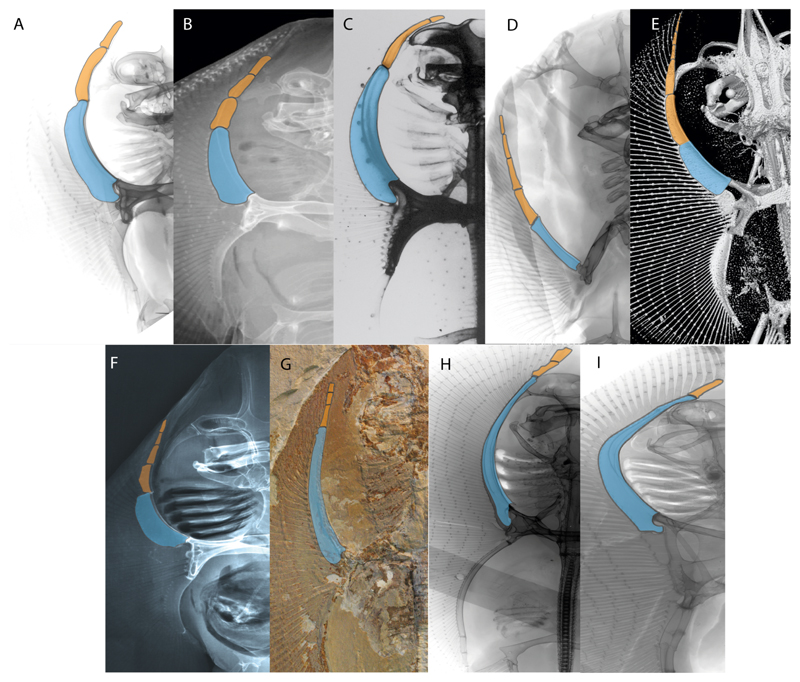
Dorsal and ventral views of the pectoral girdle of selected batomorphs. State (0): **(A)** *Platyrhina sinensis* (MNHN 1307); **(B)** Platyrhinoidis triseriata (MNHN 3211); **(C)** *Zanobatus* sp. (MNHN 1989. 12. 91); **(D)** *Narcine bancroftii* (CAS 18246); **(E)** *Raja clavata* (BRC–Raja); **(F)** *Zapteryx exasperata* (CNPE-IBUNAM 17824); **(G)** †“*Rhinobatos*” *maronita* (NHMUK-PV-P4012). State (1): **(H)** *Urolophus kaianus* (NHMUK 1879.5.14.424). State (2): **(I)** *Gymnura marmorata* (CAS-SU 11587). Propterygium colored in blue, segments of propterygium in orange.

**Ptr (see discussion Parsimony tree):** A first segment of the propterygium not reaching the nasal capsules (Figure 44A–G) is the basal state for batomorphs, being present in several taxa across all batomorphs groups: Rajiformes (*Raja, Bathyraja*, †*Ostarriraja*, †*Ptychotrygon,* †*Sclerorhynchus* and †*Libanopristis*); Torpediniformes (†*Titanonarke, Torpedo, Hypnos, Narcine, Narke, Temera, Platyrhina*, †*Eoplatyrhina* and *Platyrhinoidis);* Rhinopristiformes (†*“Rhinobatos” maronita,* †“*R*.” *latus*, †*Stahlraja*, †*Tlalocbatus, Pristis, Rhynchobatus, Glaucostegus, Rhina, Rhinobatos, Pseudobatos*, †*Eorhinobatos*, †*Pseudorhinobatos, Trygonorrhina, Zapteryx, Aptychotrema, Britobatos*, †*Iansan* and †*Rhombopterygia*); Myliobatiformes (*Zanobatus*, †*Plesiozanobatus*) and Jurassic batomorphs (†*Kimmerobatis*, †*Asterodermus*, †*Spathobatis* and †*Belemnobatis*). A first segment of the propterygium reaching the nasal capsules (Figure 44H) is a synapomorphy for clade 14, with the subsequent gain of the state “first segment of the propterygium extending beyond the level of the nasal capsule” (Figure 44I,J) as basal state for clade 15, with an independent gain in †*Cyclobatis.* This feature is also present in †*Asterotrygon*, †*Heliobatis, Hypanus* and *Gymnura,* but their polytomic arrangement renders the reconstruction difficult.

**MLtr (see discussion Maximum Likelihood tree):** Presents a similar reconstruction to that found in the parsimony analysis. However, this topology recovers the first segment of the propterygium reaching the level of nasal capsules as a synapomorphy of clade 14, with a subsequent gain of the state “first segment of the propterygium extending beyond the level of nasal capsules” as synapomorphy of clade 33.

**Figure 45:**
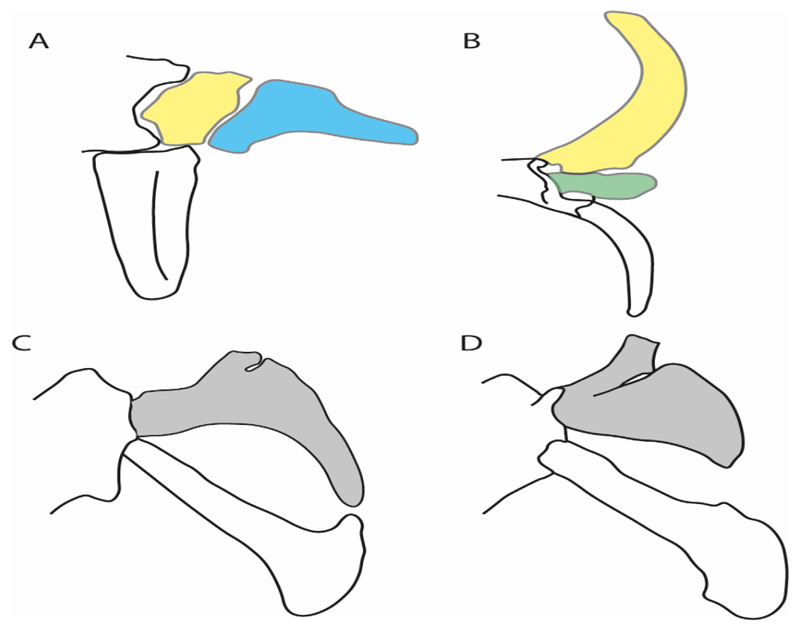
Scapulacoracoid and basal pectoral elements in dorsal and ventral views. State (0): **(A)** *Chimaera cubana* (USNM 400700, https://sharksrays.org/ (accessed on 25 March 2020)). State (1): **(B)** *Rhinobatos glaucostigma* (CNPE IBUNAM 17810). State (2): **(C)** *Hemiscyllium ocellatum* (USNM 40024) redrawn and modified from da Silva and de Carvalho Text-Figure 3F in [8]; **(D)** *Ginglymostoma cirratum* (CAS 232210) redrawn and modified from da Silva and de Carvalho Text-Figure 10F in [8]. Colors: propterygium in yellow; mesopterygium in green; anterior radial element in blue; and fusion between propterygium and mesopterygium in gray.

109. **Interaction between mesopterygium and propterygium:** (0) Fused; (1) separated. Modified from de Carvalho [38] (char. 39). *Zanobatus*, †*Plesiozanobatus, Aetobatus* and *Rhinoptera* have no mesopterygium (-). In *Harriotta* and *Chimaera* (Figure 45A) this interaction is very different (-).

**Ptr (see discussion Parsimony tree):** Separated mesopterygium and propterygium (Figure 45B) is the basal state for euselachians (hybodonts + sharks and rays). The fusion between mesopterygium and propterygium (Figure 45C), conversely, is a synapomorphy of [*Hemiscyllium* + *Ginglymostoma*].

111. **Cross-bracing of pectoral radials:** (0) Absent; (1) present. Modified from Aschliman et al. [7] (char. 67) and Shira [37] (char. 67). Based on observations by Schaefer and Summers [63].

**Figure 46:**
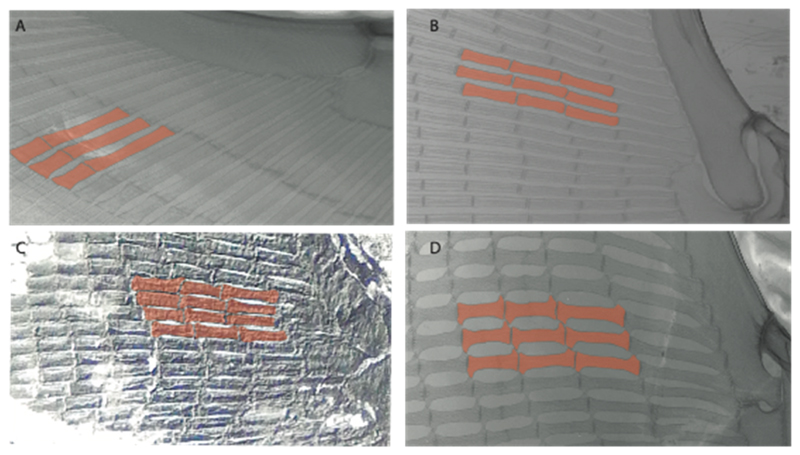
Dorsal view of a portion of the pectoral fins with radials highlighted in orange showing the cross-bracing articulation between adjacent radials. State (0): **(A)** *Pseudobatos percellens* (CAS SU11828-29); **(B)** *Urobatis halleri* (CAS SU2948). State (1): (C) †*Britobatos primarmatus* (MNHN 1946.18.94); **(D)** *Gymnura marmorata* (CAS SU1158).

**Ptr (see discussion Parsimony tree):** The presence of inter-radial connections (cross-braces) between radials of the pectoral fin (Figure 46A,B) is a shared feature of clade 15 with independent gains in †*Britobatos, Urotrygon* and *Gymnura.* The absence of cross-braces (Figure 46C,D) is basal for chondrichthyans.

**MLtr (see discussion Maximum Likelihood tree):** The presence of inter-radial connections (cross-braces) between radials of the pectoral fin is a synapomorphy of clade 15, with independent gains in †*Britobatos, Urotrygon, Gymnura.*

#### 3.1.11 Pelvic Fins

122. **Overdevelopment of first pelvic radial:** (0) Absent; (1) present (new).

**Figure 47:**
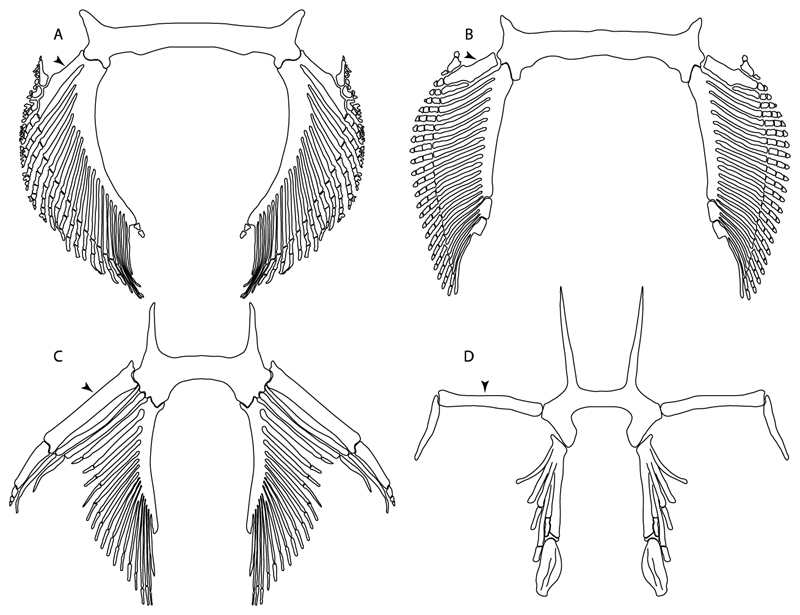
Dorsal view of pelvic girdle. State (0): **(A)** *Platyrhina sinensis* (AMNH 44055) redrawn and modified from de Silva et al. Text-Figure 2D in [10]. **(B)** *Zapteryx brevirostris* (uncatalogued material). State (1): **(C)** *Raja clavata* (BRC–Raja); **(D)** †*Cyclobatis oligodactylus*. (NHMUK PV P-601). Arrowheads: first pelvic radial.

**Ptr and MLtr (see discussion Maximum Likelihood and Parsimony trees):** The lack of an overdeveloped first pelvic radial (Figure 47A,B) is the basal feature for chondrichthyans. The presence of overdevelopment in the first pelvic radial, which in the extant taxa results in a pelvic fin with two lobes (Figure 47C,D), is a synapomorphy of clade 7.

60. **Pelvic basipterygium:** (0) Fused to first radial; (1) separated from first radial. Based on Riley et al.[110].

**Figure 48:**
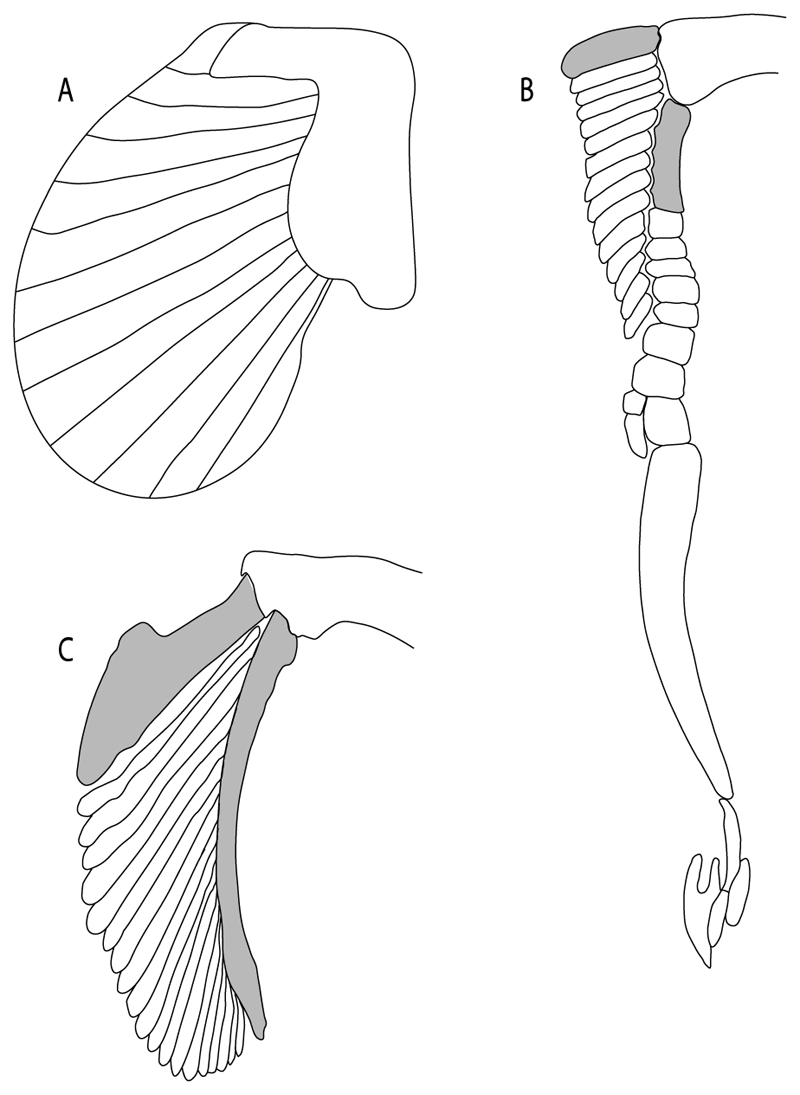
Interpretative drawings of the pelvic fins and girdles in dorsal view. State (0) **(A)** *Chimaera cubana* (USNM 400700, https://sharksrays.org/ (accessed on 25 March 2020)). **(B)** †*Hybodus hauffianus* based on Maisey Text-Figure 13A in [57]. State (1) **(C)** *Zapteryx exasperta* (CNPE-IBUNAM 20528). Basipterygium and first pelvic radial in gray.

**Ptr and MLtr (see discussion Maximum Likelihood and Parsimony trees):** There is uncertainty regarding the basal state for chondrichthyans, with *Chimaera* and *Harriotta* presenting a single element supporting their pelvic fin rays (Figure 48A), and the state for †*Doliodus*, †*Ozarcus* and †*Cobelodus* remaining unknown. The separation between the first radial and the basipterygium is the basal feature for euselachians (Figure 48B,C).

### 3.2 Phylogenetic Analyses

Parsimony analysis recovered a total of 250 most parsimonious trees (MPTs) with a tree length of 386 steps, a CI of 0.443 and a RI of 0.8411, from which a strict consensus was estimated. Maximum likelihood (ML) recovered two trees with a likelihood score of 1733.270, for which a strict consensus also was produced. Bayesian analysis ran over 190,000,000 generations, reaching an average standard deviation of split frequencies of 0.017108, a potential scale reduction factor between 0.9 and 1.2, and an average estimated sample size <100, suggesting that convergence between the chains occurred [111] (see logs in electronic Supplemental Material). These three methodological approaches recover some variation in their topological arrangements that illustrate some of the phylogenetic uncertainties surrounding the groups included in the present work.

## 4 Discussion

### 4.1 Phylogenetic Analyses

#### 4.1.1 Parsimony

**Figure 49:**
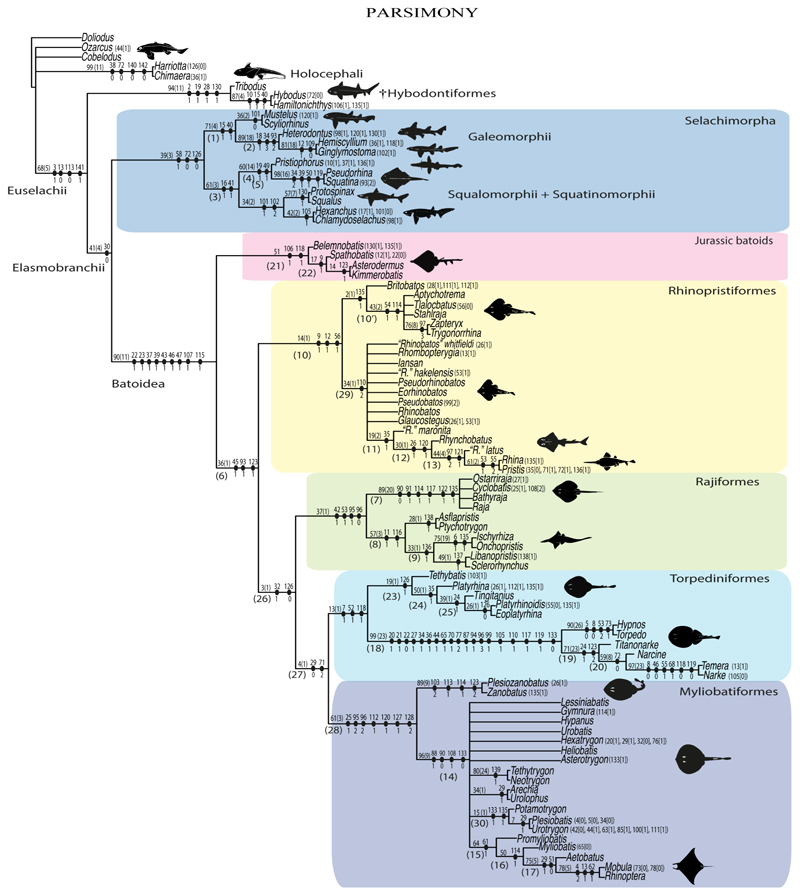
Strict consensus tree estimated from the TNT analysis. Largest groups recovered in the analysis are marked with different symbols. For the full character list see the Supplemental Material. Clade numbers in parenthesis.

Parsimony analysis did not recover a monophyletic group consisting of [Holocephali + symmoriids (represented by †*Cobelodus* and †*Ozarcus*)]. The symmoriids (†*Cobelodus* and †*Ozarcus*) and holocephalans (*Chimaera* and *Harriotta*) were placed in a polytomy along with the Euselachian clade (*sensu* [1,12,112]) (Figure 49). This arrangement might suggest alternative affinities for †*Cobelodus* and †*Ozarcus* outside the Holocephali, in contrast to previous phylogenetic analyses (e.g., [25,26,113]). However, a more extensive taxon and character sampling is mandatory to recover a more reliable systematic position for these groups, which could not be resolved using the present data matrix.

A monophyletic clade representing the Euselachii (*sensu* [112,113]) was recovered, and within this group, a sister group relation between Hybodontiformes and Elasmobranchii (*sensu* [1,112]) was found. This relationship has been previously suggested by Maisey et al. [12] and Frey et al. [114]. However, before referring to any possible elasmobranch apomorphies, a more detailed sampling of taxa with the inclusion of synechodontids or xenacanthids and other extinct groups would be desirable.

Similar to molecular analysis (e.g., [40,41]), parsimony analysis recovered a sister-group relationship between members of the Elasmobranchii (i.e., Selachimorpha and Batomorpha). Within the selachimorphs, two monophyletic groups were recovered: Galemorphii and [Squalomorphii + Squatinomorphii]. Within Squatinomorphii, there is a close relationship between *Pristiophorus* and the angel sharks *Squatina* and †*Pseudorhina,* as previously suggested by Maisey et al. [83] and some molecular analyses (e.g., [3]).

The order-level relationships recovered for Batomorpha by this analysis are summarized as follows: [Jurassic batomorphs + [Rhinopristiformes + [Rajiformes + [Torpediniformes + Myliobatiformes]]]]. The Jurassic genera †*Spathobatis*, †*Belemnobatis*, †*Asterodermus* and †*Kimmerobatis* were the first to diverge, forming a monophyletic group in a sister relationship to the remaining extant and fossil clades (crown group).

The Rhinopristiformes form a clade that includes the fossil taxa †*Britobatos*, †*Tlalocbatos*, †*Stahlraja,* †*“Rhinobatos” whitfieldi,* †“*R*.” *hakelensis,* †“*R*.” *maronita,* †“*R*.” *latus*, †*lansan*, †*Pseudorhinobatos*, †*Eorhinobatos* and †*Rhombopterygia.* This clade is the sister group to all remaining taxa. The fossil species † *“Rhinobatos” maronita* and †“*R*.” *latus* are closely related to *Rhynchobatus, Rhina* and *Pristis,* as previously suggested by Claeson et al. [23] and the family Trygonorrhinidae forms a monophyletic group that includes †*Tlalocbatos* and †*Stahlraja* as suggested by Brito et al. [115,116].

The Rajiformes form a monophyletic group that includes the extinct Sclerorhynchoidei as sister to the Rajoidei clade formed by *Raja, Bathyraja, †Cyclobatis* and †*Ostarriraja.* Within the Sclerorhynchoidei, three groups at the family level are recognized [117]: †Ptychotrygonidae, which includes †*Ptychotrygon* and †*Asflapristis;* †*Sclerorhynchidae*, which includes †*Sclerorhynchus* and †*Libanopristis;* and †Onchopristidae, including †*Onchopristis* and †*Ischyrhiza.*

Unlike in previous parsimony analyses (e.g., [23,32,33,116]), the present study recovered a close relationship between electric rays (Torpedinoidei) and thornbacks (Platyrhinoidei) that form a monophyletic group—the order Torpediniformes, also recognized by molecular analyses (e.g., [41,42]) (Figure 49). In particular, parsimony analysis agrees with the placement of †*Tethybatis*, †*Tingitanius*, †*Eoplatyrhina* and *Platyrhinoidis* as members of a monophyletic Platyrhinoidei [23,33,67].

The Torpediniformes are sisters to the Myliobatiformes. However, this grouping seems to be a by-product of the use of reductive coding [118], as the characters that support this assemblage present odd reconstructions [119]. †*Titanonarke, Torpedo, Narcine* and *Temera* lack a postorbital process [7,28], meaning that the possibility to recognize a “postorbital process separated from the triangular process” for this group is impossible. Similarly, the configuration of the coracohyoideus muscle plate (char. 71: which is absent in *Torpedo, Hypnos, Narcine,* Narke and *Temera* [6,7]) cannot be detected in torpediniforms. Both character reconstructions suggest that reductive coding might not be the best approach for these features.

A monophyletic clade including stingrays (Myliobatoidei) and panrays (*Zanobatus* and †*Plesiozanobatus*) was recovered and corresponds to the order Myliobatiformes. Within the Myliobatoidei, benthic marine stingrays, freshwater stingrays and butterfly rays are grouped in a polytomy, while pelagic stingrays form a monophyletic group that includes †*Promyliobatis,* as suggested by Marramà et al. [31].

#### 4.1.2 Maximum-Likelihood Analysis (ML)

**Figure 50:**
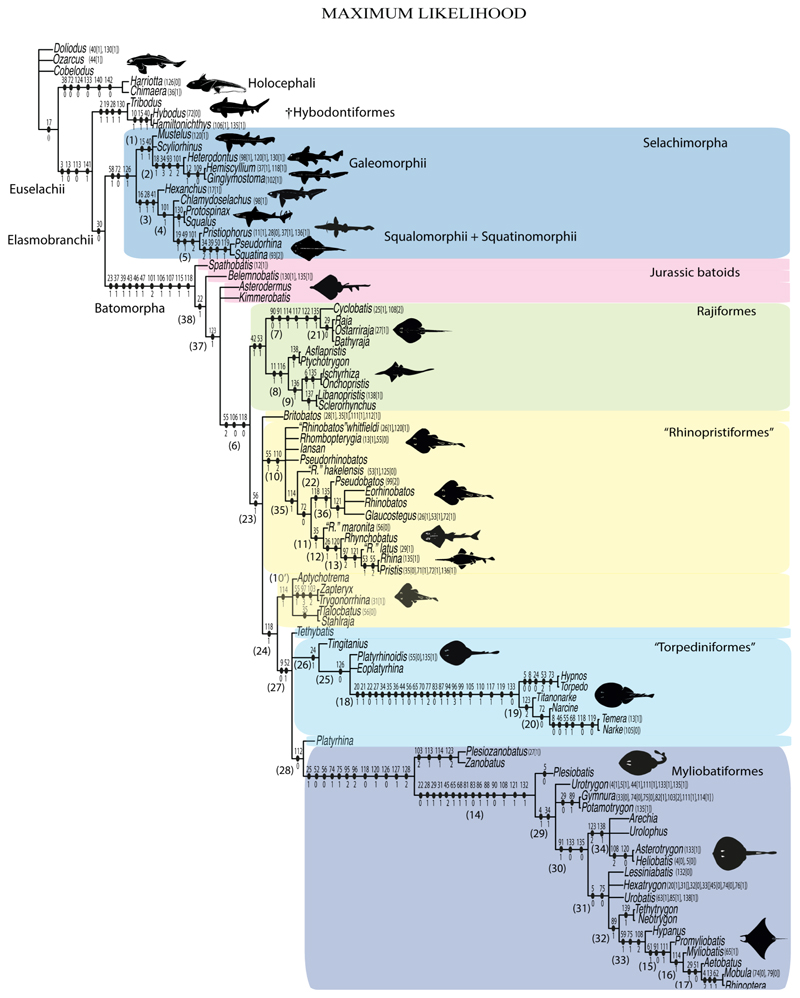
Strict consensus tree estimated from maximum-likelihood analysis in PAUP, after the selection of the trees with the best scores. For the full character list see the Supplemental Material. Clade numbers in parenthesis.

In the ML topology, †*Doliodus,* symmoriids and living chimaeroids form a paraphyletic assemblage, among which the monophyletic group consisting of *Chimaera* and *Harriotta* is sister to the remaining taxa (Figure 50).

The ML analysis recovered a monophyletic Euselachii, with a sister relationship between the monophyletic †Hybodontiformes and the Elasmobranchii (*sensu* [13,14]), which agrees with the parsimony hypothesis.

The intrarelationships of Selachimorpha are like those recovered in the parsimony analysis, with a monophyletic Galemorphii as sister to [Squalomorphii + Squatinomorphii] group, with a close relationship between *Pristiophorus* and the angel sharks, *Squatina* and †*Pseudorhina.* The identification of a clade formed by [Galeomorphii + [Squalimorphii + Squatinomorphii]] and the stable placement of the extinct sharks, †*Protospinax* and †*Pseudorhina* in both parsimony and ML analyses, is promising and suggests that it is possible to include more extinct sharks in the present matrix to evaluate their systematic position.

A monophyletic Batomorpha clade with some differences compared to the parsimony tree is recovered in the ML topology (compare Figures 49 and 50). Unlike the parsimony tree, the ML did not recover a monophyletic group formed by the Jurassic batomorphs, but placed them as successive sister taxa to the remaining batoids.

Unlike in the parsimony topology, the monophyletic Rajiformes [†*Cyclobatis* + [*Raja* + †*Ostarriraja* + *Bathyraja*]] + †*Sclerorhynchoidei*] is placed as sister to the remaining batomorphs like in molecular analyses (e.g., [41,42]). The relationships within Rajoids are better resolved in the ML than in the parsimony analysis (compare Figures 49 and 50), with †*Cyclobatis* being the sister taxon to the polyphyletic clade [Raja + †*Ostarriraja + Bathyraja*]. Within the †Sclerorhynchoidei, the same three groups previously recovered by families are retained: †Ptychotrygonidae, †Onchopristidae and †Sclerorhynchidae.

The remaining taxa are recovered in a clade in which both “Rhinopristiformes” and “Torpediniformes” being paraphyletic. Within this group there is uncertainty in the placement of †*Britobatos,* which is not unexpected, considering that it shares several features with various groups, including a broad postorbital process (char. 28; shared with Myliobatiformes), vertebral centra in the “synarcual” reaching only the caudal portion of the suprascapula (char. 55; like Myliobatiformes), the presence of a rostral appendix (char. 9; like Rhinopristiformes), differentiated lingual lateral uvulae in teeth (char. 56; like Rhinopristiformes) and the cross-bracing of pectoral radials (char. 111; like *Gymnura, Urotrygon* and pelagic stingrays).

The paraphyletic status of Rhinopristiformes is due to the placement of the Trygonorrhinidae (including †*Tlalocbatos* and †*Stahlraja*) in the myliobatiforms and torpediniforms clade, sharing with the electric rays the presence of a postpelvic process (char. 118 [1]) (Figure 50).

The paraphyly of the “Torpediniformes” is due to the polyphyly of the thornback rays “Platyrhinidae” since *Platyrhina* is recovered as sister to the Myliobatiformes. From a morphological point of view, *Platyrhina* shows several similarities with Myliobatiformes (such as the proximal portion of the propterygium extending beyond the mesocondyle), which causes its position closer to myliobatiforms than to other “platyrhinids” in the ML tree. Within “Torpediniformes”, the electric rays (or Torpedinoidei) form a monophyletic group [[*Torpedo* + *Hypnos*] + [†*Titanonarke* + [*Narcine* + [*Temera* + Narke]]]].

The ML tree recovered Myliobatiformes as monophyletic, with the panrays Zanobatidae (i.e., *Zanobatus* and †*Plesiozanobatus*) as sister to Myliobatoidei. Within Myliobatoidei, the phylogenetic relations recovered in the ML tree differ from molecular analyses (see [41,42]), with *Gymnura* being recovered in a close relationship to *Potamotrygon,* separated from *Urolophus, Hexatrygon* and *Plesiobatis.*

#### 4.1.3 Bayesian Inference Analysis (BI)

**Figure 51:**
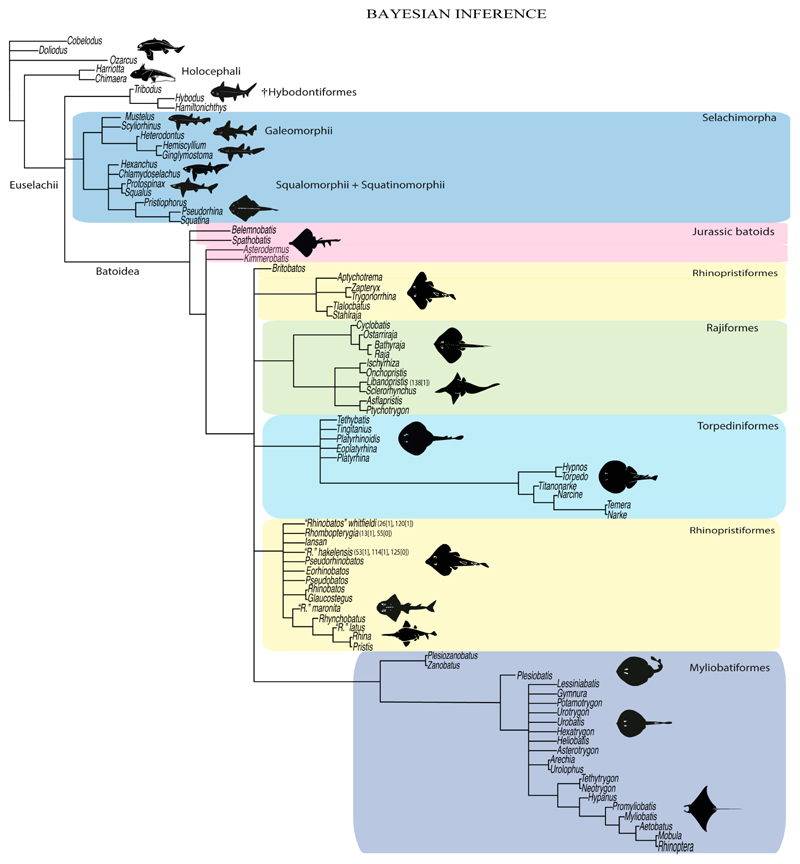
Posterior probability tree estimated from the Bayesian inference analysis in MrBayes. Main groups recovered in the analysis are marked with different symbols.

In the BI, the Holocephali is not recovered monophyletically if the symmoriids are included (Figure 51). Nevertheless, the living chimaeroids *Chimaera* and *Harriotta* form a monophyletic group in a sister relationship to a monophyletic Euselachii. Within this latter clade, the hybodontiforms, selachimorphs and batomorphs are placed in a polytomous relationship.

The BI analysis also recovered a monophyletic Selachimorpha, with similar relations within its components as previous topologies (see Figures 49 and 50) and those established by molecular analyses (e.g., [41]).

Batomorpha form a monophyletic group like in previous analyses (Figures 49–51). As in the ML tree, the Jurassic taxa are not monophyletic but arranged as successive sister taxa with †*Spathobatis* and †*Belemnobatis,* and †*Asterodermus* and †*Kimmerobatis,* respectively, seemingly being more closely related to each other. The remaining Cretaceous and Cenozoic batomorphs form a monophyletic group. However, all the orders within this group, i.e., Myliobatiformes, Torpediniformes, Rajiformes and a paraphyletic “Rhinopristiformes” fall in polytomy.

This analysis also supports the placement of sclerorhynchoids as sister to the remaining fossil and living rajoids. The relations within this order are more resolved than in the ML analysis with †*Cyclobatis* being placed as the sister taxon to a clade that includes †*Ostarriraja, Raja* and *Bathyraja,* in which †*Ostarriraja* is the sister group of the *Raja* and *Bathyraja* group. Within the Sclerorhynchoidei, the †Ptychotrygonidae, †Onchopristidae and †Sclerorhynchidae are also recovered, forming a monophyletic clade (Figure 51).

Similar to the ML tree in the BI analysis, the Trygonorrhinidae are again separated from the main “Rhinopristiformes” clade, which includes the fossil taxa †*“Rhinobatos” whitfieldi,* †“*R*.” *hakelensis,* †“*R*.” *maronita,* †“*R*.” *latus*, †*Iansan*, †*Pseudorhinobatos*, †*Eorhinobatos* and †*Rhombopterygia,* consequently recovering a paraphyletic arrangement for this order.

Interestingly, the Platyrhinoidei and the Torpedinoidei form a monophyletic group similar to the parsimony tree and molecular analyses (e.g., [41,42]). The intrarelations of the Platyrhinoidei taxa are completely unresolved. In this perspective, their polytomic arrangement suggests that the thornbacks need a more in-depth revision of their characters.

The BI tree places stingrays (Myliobatoidei) and panrays (*Zanobatus* and †*Plesiozanobatus*) in a monophyletic clade, the Myliobatiformes. Within this clade there is a large polytomy that includes most of the stingrays. Within the pelagic stingrays, a more resolved topology is recovered, with †*Tethytrygon* and *Neotrygon* being the sister clade to the [*Hypanus*, [†*Promyliobatis*, [*Myliobatis*, [*Aetobatus*, [*Mobula* and *Rhinoptera*]]]]] clade.

### 4.2 Phylogenetic Considerations

**Figure 52:**
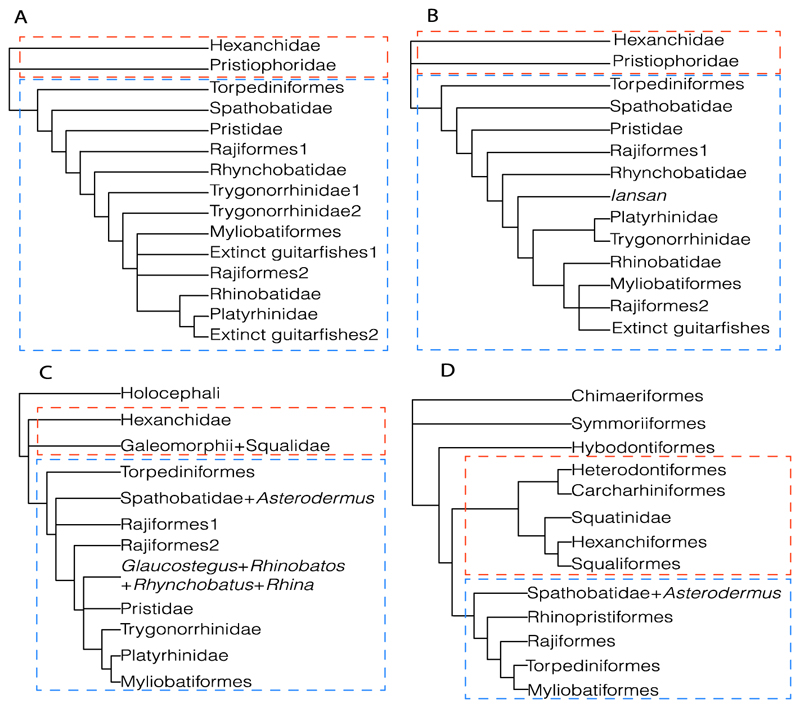
Simplified trees form the strict consensus tree from the parsimony analyses of previous works showing the differences in the relationship between the elasmobranch groups. Red rectangle: Selachimorpha; blue rectangle: Batomorpha. **(A)** Underwood and Claeson [24]; **(B)** Brito et al. [116]; **(C)** Marramà et al. [33]; **(D)** Present work.

With the inclusion of fossil taxa and resampling of characters in the present analyses, we recovered monophyletic Hybodontiformes, Selachimorpha and Batomorpha. Unlike previous morphological analyses (e.g., [23,24,32,33,115,120]), both parsimony and maximum likelihood recovered at sister-group) relationship between selchimorphs and batomorphs (Figures 50 and 52).

All three analyses place the Jurassic batomorphs in a sister relation to all the remaining batomorphs [2] (Figures 49–51). Interestingly, the thornbacks (Platyrhinoidei) were recovered sister to the electric rays (Torpedinoidei) forming the order Torpediniformes; in both BI and parsimony a relationship never recovered under morphology-based analyses. However, the phylogenetic relationships of Rhinopristiformes and the recognition of its monophyly, as most recent taxonomic studies suggest [2,3], continues to be problematic, with their monophyly not being consistently found even in molecular analyses (e.g., [42]) and their composition also differing (e.g., [41,42]) between these studies. In the present study, only the parsimony analysis recovered the Rhinopristiformes as a monophyletic group (Figure 49) while maximum-likelihood (ML) and Bayesian inference (BI) analyses recovered two groups of Rhinopristiformes, with the family Trygonorrhinidae (*Trygonorrhina, Zapteryx* and *Aptychotrema*) consistently found as a separate clade (Figures 50 and 51). All the analyses suggest close relation of the fossil taxa †*Stahlraja* and †*Tlalocbatos* within the Trygonorrhinidae, therefore placing the origin of this family well into the early Cretaceous (Albian–Aptian), suggesting a long separated evolutionary history between Trygonorrhinidae and the remaining members of the Rhinopristiformes.

The present analyses also included the species *Pseudobatos productus (Rhinobatos)* and *Pseudobatos lentiginosus (Pseudobatos),* both exhibiting significant variations in their skeletal morphologies, especially of their pectoral girdles. These differences contradict their placement in a single genus, supporting the separation of the American Pacific and Atlantic *"Pseudobatos"* into different genera. Overall, the present results indicate that the Rhinopristiformes are a group still in urgent need of in-depth phylogenetic studies, before any taxonomic rearrangement is proposed.

The living (*Raja* and *Bathyraja*) and fossil (†*Ostarriraja* and †*Cyclobatis*) skates are grouped with sclerorhynchoids, which is consistent with previous analyses that recovered this relationship based on features of the branchial skeleton [32,117]. Rajiformes show a rather intriguing suite of morphological characters, which are also present in several batomorph groups, including rostral appendices (also observed in Rhinopristiformes), catenated calcification on the pectoral-fin radials and lack of a postpelvic process (also observed in Myliobatiformes). The sharing of these features with guitarfishes and myliobatiforms ultimately produces the distinct systematic placement displayed by this group in the parsimony and ML topologies, with the BI analysis neither supporting nor contradicting these arrangements.

As far as the catenated calcification pattern of radials is concerned, which is of a different type in rajoids (two-chained) and some benthic stingrays (four-chained) [63], we hypothesize that “catenated” two-chained and “catenated” four-chained represent two different calcification patterns that evolved independently. Overall, the placement of the Rajiformes in the ML topology resembles that recovered by molecular analyses [41,42], favoring the placement of Rajiformes as sister group to all the other Cretaceous and Cenozoic batoids, which might be a point in favor of the ML topology. However, the ML also recovered some problematic arrangements such as that of *Platyrhina* as sister taxon to the Myliobatiformes.

## 5 Conclusions

The advantages and drawbacks of the various phylogenetic methods are extensively discussed in a series of papers (e.g., [121–126]. With these criteria responding differently to the patterns in the datasets, it is not surprising that different topologies were found in the present study. O’Reilly et al. [121,122] proposed that model-basis methods are more accurate than parsimony when analyzing morphological data. However, Goloboff et al. [123,124] showed that this happens under specific parameters and that parsimony analyses also have the potential to be very informative.

The present analysis focusing on batomorph relationships reveals the need for an even more profound selachian sampling (extinct and extant species), when comparing the phylogenetic topologies recovered under the different criteria, or when evaluating character reconstructions and re-evaluating the codification of morphological characters.

Maximum-likelihood and parsimony analyses recovered more resolved topologies than Bayesian inference. However, ML and parsimony disagree in some arrangements (e.g., in the placement of some batomorph groups, the monophyletic status of Rhinopristiformes, the phylogenetic placement of †*Britobatos* and the phylogenetic relationships of Torpediniformes), these different suggest areas of interest for future works. Our results also indicate that a revision of Jurassic batomorphs and selachimorphs is of utmost importance to provide a more consistent topology, especially related to deeper nodes. Such a morphological-trait revision also has the potential to better understand the composition of some groups recognized by molecular analyses but not in morphological analyses (e.g., Rhinopristiformes and Torpediniformes).

While the relationships between selachimorphs and batomorphs are not completely resolved in the present analysis, our character and taxon sampling presents a persistent placement of most of the fossil taxa within certain clades (orders and families), and their consistent similar relationships in all tree topologies—often similar to those previously hypothesized (e.g., [23,24,41,127])—is promising and indicates that the inclusion of more fossil taxa in the present matrix likely will not cause loss of resolution, therefore suggesting that a strong phylogenetic signal can be recovered from fossil taxa.

## Supplementary Material

Supplementary Information

## Data Availability

All data used by the authors for the analysis is available in the supplementary materials, which can be downloaded at: www.mdpi.com/xxx/s1.
