# Supplementary material for: The Phylogeny of Rays and Skates (Chondrichthyes: Elasmobranchii) Based on Morphological Characters Revisited
Source: Diversity (Basel). Author manuscript; Available in PMC 2022 Jun 22. (PMC7612883; doi:10.3390/d14060456)
Supplement: Supplementary Information [file EMS145860-supplement-Supplementary_Information.zip › diversity-1700431-Characters.pdf]

Supplementary information

Abbreviations: AMNH: American Museum of Natural History, USA; **ANSP**: Academy of Natural Sciences of Drexel University, USA; **BHN:** Musèe d'Histoire Naturelle de Boulogne-sur-Mer; **BPBM:** Bernice P. Bishop Museum, Honolulu, Hawaii; **BRC**: Birkbeck Reference Collection, UK, **BSP:** Bayerische Staatssammlung für Paläontologie und Geologie, Munich, Germany; **CSIRO**: Commonwealth Scientific and Industrial Research Organization, Australia; **CAS**: California Academy of Sciencies, San Francisco, California, USA; **CNPE-IBUNAM**: Colección Nacional de Peces del Instituto de Biología, Universidad Nacional Autónoma de México, México; **DAE**: D.A. Ebert field number; **FMNH**: Field Museum of Natural History, Chicago, Illinois, USA; **FSFRL**: Far Seas Fisheries Research Laboratory, Shimizu, Japan; **GMBL**: College of Charleston, Grice Marine Biological Laboratory, Charleston, South Carolina, USA; **HO: HUMZ:** Hokkaido University Laboratory of Marine Zoology, Japan; **IGM:** Colección Nacional de Paleontología del Instituto de Geologia, Universidad Nacional Autónoma de México, Mexico; **IGR:** Geological Institute of the University of Rennes 1; **IPUW**: Palaeontological Collections of the University of Vienna; **JM-SOS**: Jura Museum Eichtätt, Germany; **K:** Etches Collection, Kimmeridge, UK; **LACM:** Los Angeles County Museum of Natural History, USA; **MB.F:** Museum für Naturkunde, Berlin, Germany; **MCSNV**, Museo Civico di Storia Naturale, Verona, Italy; **MCZ**: Museum for Comparative Zoology, Cambridge, USA; **MFSN**: Museo Friulano di Storia Naturale di Udine; **MGGC**: Museo Geologico Giovanni Capellini, Italy; **MGP-PD:** Museo di Geologia e Paleontologia dell'Universita degli Studi di Padova**,** Italy; **MHNL:** Musée des Confluences, Lyon, France; **MNHN** Muséum National d'Histoire Naturelle, Paris; **MNRJ:** Museu Nacional do Rio de Janeiro; **MSM:** Marine Science Museum, Tokai University, Japan; **MSNFI:** Museo di Storia Naturale dell'Università di Firenze; **MSNPV:** Museo di Storia Naturale dell'Università di Pavia, Italy; **MZUSP:** Universidade de Sao Paulo, Museu de Zoologia, Brazil; **NHMUK PV P:** Natural History Museum United Kingdom, UK, Palaeontology Vertebrates; **NHMW:** Naturhistorisches Museum Wien, Wien, Austria; **SAM**: South African Museum, Cape Town, South Africa; **SIO:** Scripps Institution of Oceanography, USA; **SMNS**: Staatliches Museum für Naturkunde Stuttgart, Germany; **UF**: University of Florida, Florida State Museum, USA; **UREJ:** Universidade do Estado do Rio de Janeiro, Brazil; **USNM**: National Museum of Natural History, USA; **UT**: University of Tennessee, Department of Zoology, USA; **ZMB**: Museum für Naturkunde zu Berlin, Germany.

Material examined (Morphology): ***Amblyraja radiata*** BRC—Amblyraja. ***Aptychotrema vincentiana*** CSIRO 101 https://sharksrays.org/. †***Arechia crassicaudata*** MCSNV IG.VR.27607, VII.B.82/83, 84/85, T.317/318, MGP-PD 8875C/76C, NHMW 1853-0027-0005. †***Asflapristis cristadentis*** NHMUK PV P73925, 75428a-e, 75429a-d, 75431, 75432, 75433. †***Asterodermus platypterus*** NHMUK PV P12067, 10934; JM-SOS-3647. †***Asterotrygon maloneyi*** AMNH P 11557 FMNH PF 12914, 12989, 12990, 14069, 14097, 14098, 14567, 15166, 15180. ***Bathyraja leucomelanos*** MNHN 2005-2740. †***Belemnobatis sismondae*** MHNL 15.263, 15.264, 15.753. †***Britobatos primarmatus*** MNHN 1946.18.94, NHMUK PV P4015, 4016, 49517. ***Callorhinchus capensis*** AMNH 36943. ***Chimaera cubana*** USNM 400700 https://sharksrays.org/. ***Chimaera monstrosa*** Base on Dean *et al.*, [51]. ***Chlamydoselachus anguineus*** (MSM-88-40, MZUSP 110974, UF 44302 https://sharksrays.org/. ***Chlamydoselachus africana*** SAM 36076. †***Cobelodus aculeatus*** FMNHPF 2628, 3090, 7324, 7343, 7345, 7472, 7475, 8006, 8011(*based on* Zangerl and Case [50] and Maisey *et al.*, [49]). †***Cyclobatis major*** MNHN 1939-13-334A, MNHN HAK555. NHMUK P 4010, 4011, 49514 63175. †***Cyclobatis radians*** NHMUK P 61243. †***Cyclobatis tuberculatos*** NHMUK PV P 10436. †***Cyclobatis oligodactylus*** NHMUKPV P 601, MNHN F.HDJ504, F.HDJ505. †***Cyclobatis sp.*** AMNH 10946, MNHN HAK 550. †***Doliodus latispinosus*** NBMG 10127 (*based on* Miller *et al.*, [51]; Maisey *et al.*, [49]). †***Eoplatyrhina bolcensis*** MGP-

PD 8873C, 8874C, 26279C, MGGC 7449. †***Eorhinobatos primaevus*** MGP-PD 26278 Based on [52]. ***Ginglymostoma cirratum*** CAS 232210, USNM 127110. ***Glaucostegus typus*** (NHMUK 1967.2.11.3, 2012.2.8.54, 1926.5.26.5). ***Gymnura altavela*** (https://sharksrays.org). ***Gymnura japonica*** HUMZ 48301. ***Gymnura marmorata*** CAS SU1158. ***Gymnura micrura*** FMNH 89990. ***Harriotta raleighana*** USNM 320579. ***Hemiscyllium ocellatum*** AMNH 38151, 44128, USNM 40024. †***Heliobatis radians*** AMNH P 19665, FMNH PF 2020. ***Heterodontus francisci*** AMNH 96795, 217862, MZUSP 112022. ***Heterodontus zebra*** HUMZ 37666. ***Hexanchus griseus*** CAS uncatalogued, HUMZ 95104. ***Hexatrygon bickelli*** J.L.B. Smith Institute of Ichthyology catalogue no. 997 [17]. ***Hexanchus nakamurai*** DAE 881504, UF 165855 https://sharksrays.org. ***Egertonodus basanus*** NHMUK PV P 40718, 2082, 60110, 6356, AMNH 4692. †***Hybodus reticulatus*** NHMUK PV P 3168. †***Hybodus fraasi*** BSP 1899-1-2. †***Hybodus hauffianus*** NHMUK PV P 6356, SMNS 10062, 15150. ***Hydrolagus affinis*** BRC—Hydrolagus, skeleton. ***Hypanus americanus*** (https://sharksrays.org/, AMNH 30607, ANSP 103931)**.** ***Hypanus sabinus*** TCWC 2591.1. ***Hypnos monopterygius*** (USNM 84374 https://sharksrays.org). ***Hypnos subnigrum*** MCZ S985. †***Iansan beurleni***, DGM-917, 918, NHMUK P62947 (1). †***Ischyrhiza mira*** AMNH FF 20388. †***Kimmerobatis etchesi*** K874, K1894. †***Lessiniabatis aenigmatica*** MNHN F.Bol.566, MSNFI IGF 103555, MFSN GP.864, †***Libanopristis hiram*** NHMUK PV P108705, 108706, 13858, 63610, 75075. ***Mobula munkiana*** (SIO 85-34 https://sharksrays.org). ***Mustelus canis*** ANSP 178683, ***Mustelus manazo*** https://sharksrays.org/. ***Narcine bancroftii*** CAS 18246. ***Narcine brasiliensis*** CNPE-IBUNAM 9280, AMNH 77069, https://sharksrays.org/. ***Narcine entemedor*** CNPEIBUNAM 5807. ***Narcine tasmaniensis*** NHMUK 1961. ***Narke japonica*** HUMZ 94970, USNM 127110. ***Neotrygon kuhlii*** (https://sharksrays.org/). †***Ozarcus mapesae*** AMNH FF 20544 (based on Pradel *et al*., [14]). †***Ostarriraja parva*** NHMW 2005z0283/0097a, 2005z0283/0097b. †***Onchopristis numidus*** NHMUK PV P 75502, 75503, 74040, 74045, 74047, 74050, 74051, 74052, 74053, 74054; IPUW 353500; IGR 2818, 2819, 2820, 2821. ***Potamotrygon limai*** MZUSP 104033, 104068. ***Potamotrygon motoro*** AMNH 97428 https://sharksrays.org/ †***Plesiozanobatus egertoni*** MGP-PD 154Z, MB.F 1608.1 MCSNV VII.B.81. ***Platyrhina sinensis*** MNHN 1307, AMNH 44055. ***Platyrhina* sp** BRC—Platyrhina, CT scan. ***Platyrhinoidis triseriata*** MNHN 4329, 3211, USNM 26275 https://sharksrays.org/. ***Plesiobatis daviesi*** (BPBM 24578). ***Pristis pectinata*** MCZ 36960. ***Pristis* sp**. BRC—Pristis. NHMUK 21478347. ***Pristis zijsron*** ANSP 101398. ***Pristiophorus japonicus*** MCZ 1045-s, UT 24174, HUMZ 49349. ***Pristiophorus nudipinnis*** CSIRO 3731, FSFL-EA735. ***Pristiophorus schroederi*** FLMNH 24621. †***Promyliobatis gazolae*** MCSNV VII.B.90, MSNPV 14620. †***Protospinax annectans*** MCZ 278, 6394, NHML P8775. †***Ptychotrygon rostrispatula*** NHMUK PV P73630, 75496, 75497, 75498, 75500. ***Pseudobatos percellens*** UERJ 1240, CAS SU11828-29. ***Pseudobatos lentiginosus*** CNPEIBUNAM 17827. †***Pseudorhina alifera*** NHM P8535, SOS 438. †***Pseudorhina acanthoderma*** SMNS 86214/41, 5735. ***Pseudorhinobatos dezignii*** NHMW 1853.XVII.4 [2] ***Raja clavata*** BRC—Raja, CT scan. ***Raja eglanteria*** CT scan in https:// sharksrays.org. ***Rajella fyllale*** BRC—Rajella, skeleton. ***Rhina ancylostoma*** LACM 38117 https://sharksrays.org/, NHMUK 1884, 1925. ***Rhinobatos "Pseudobatos" glaucostigma*** CNPE-IBUNAM 17810. ***Rhinobatos "Pseudobatos" horkelli*** UERJ 1397. ***Pseudobatos lentiginosus*** CNPEIBUNAM 17827. ***Rhinobatos "Pseudobatos" leucorhynchus*** CNPEIBUNAM1039. ***Rhinobatos "Pseudobatos"* productus** CNPE-IBUNAM 17829, 17821. ***Rhinoptera bonansus*** (BRC—Rhinoptera, GMBL 73 https://sharksrays.org. ***Rhinoptera javanica*** HUMZ 97698. ***Rhinoptera jayakari*** (Pradeep *et al*., [53], Text-Figure 3A). ***Rhynchobatus djiddensis*** MNHN 7850, MCZ 806. ***Rhynchobatus lubberti*** MNHN 50-22-04.80. ***Rhynchobatus sp.*** BCR—Rhynchobatus, skeleton. ***Rhynchobatus springeri*** https://sharksrays.org/ †***Rhinobatos" maronita*** MNHN 1946.17.274, NHMUK P4012, 48215, 10696, 39233, 49511. †***Rhinobatos" latus*** NHMUK PV P4014. †***Rhombopterygia rajoides***, MNHN.F.HDJ 483. ***Scyliorhinus cabofriensis*** UERJ 2231.4. ***Scyliorhinus meadi*** GMBL 8312 73 https://sharksrays.org. †***Shizorhiza stromeri*** NHMUK PV P 73625. †***Sclerorhynchus atavus*** NHMUK PVP4017, 4776, 49546, 49518, 49533, 49547. †***Spathobatis bugesicus*** (NHMUK PV

P 6010, 2099 (2), 12067, BSP 1952-I-82, AS-I-505). †***Spathobatis moorbergensis*** BHN 2P1. ***Squalus acanthias*** HUMZ 87733, GMBL 7313. ***Squalus brevirostris*** AMNH 258171. ***Squalus mitsukurii*** https://sharksrays.org/. ***Squatina californica*** AMNH 55686. ***Squatina guggenheim*** MZUSP 110871. ***Squatina nebulosa*** AMNH 258172. †***Stahlraja sertanensis***, UERJ-PMB 400, MPSC-P 099. ***Temera hardwickii*** NHMUK 1984.1.18.6, 1887.4.16.14. †***Tethytrygon muricatus*** MGP-PD 159Z/160Z, 150Z/151Z, MCSNV IG.23194, IG.186653, T.1021, MCSNV II.B.92, MNHN F.Bol568. †***Tingitanius tenuimandibulus*** NHMUK PV P66857. †***Titanonarke molini*** MCSNV IG. VR.67290. †***Titanonarke megapterygia*** MCSNV IG.135576. †***Tlalocbatus applegatei*** IGM 5853. ***Torpedo fuscomaculata*** USNM, https://sharksrays.org/. ***Torpedo sp.*** NHMUK 72261. ***Torpedo ocellata*** AMNH 4128. †***Tribodus limae*** AMNH FF13958, FF 13959, MNRJ 105. ***Trygonorrhina fasciata*** MNHN 1372; BRC—Trygonorrhina, MCZ982S. ***Urobatis halleri*** CAS 17327, SU2948, FMNH 42601. ***Urobatis jamaicensis*** AMNH 30385. ***Urolophus aurantiacus*** AMNH 258305 https://sharksrays.org). ***Urolophus kaianus*** NHMUK 1879.5.14.424. ***Urotrygon chilensis*** FMNH 93737, AMNH 258305 https://sharksrays.org. ***Urotrygon venezuelae*** AMNH55623. ***Zanobatus sp.*** MNHN 1989.12.91. ***Zanobatus schoenleinii*** MNHN N/C, UF 176858, https://sharksrays.org/. ***Zapteryx brevirostris*** UERJ PMB35 UERJ 1234, 1237. ***Zapteryx exasperata*** CNPE-IBUNAM 17822, 17823, 17824, 17826, 17825, 20528. ***Zapteryx xyster*** CNPE-IBUNAM 16661, 19790.

Apomorphies supporting the phylogenies

PARSIMONY

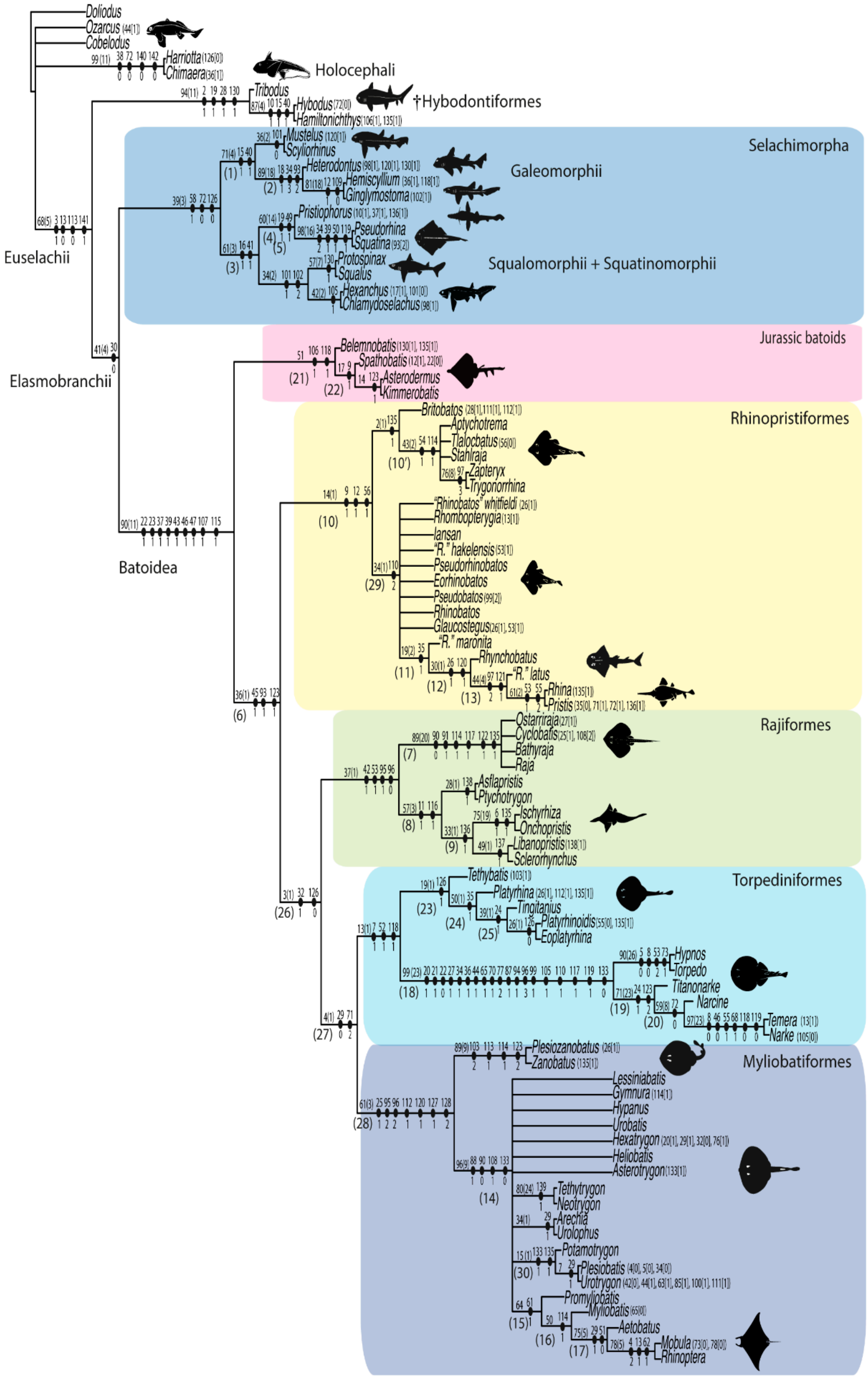

Figure S1. Strict consensus tree estimated from the TNT analysis. Largest groups recovered in the analysis are marked with different symbols. Clade numbers in parentheses.

MAXIMUM LIKELIHOOD

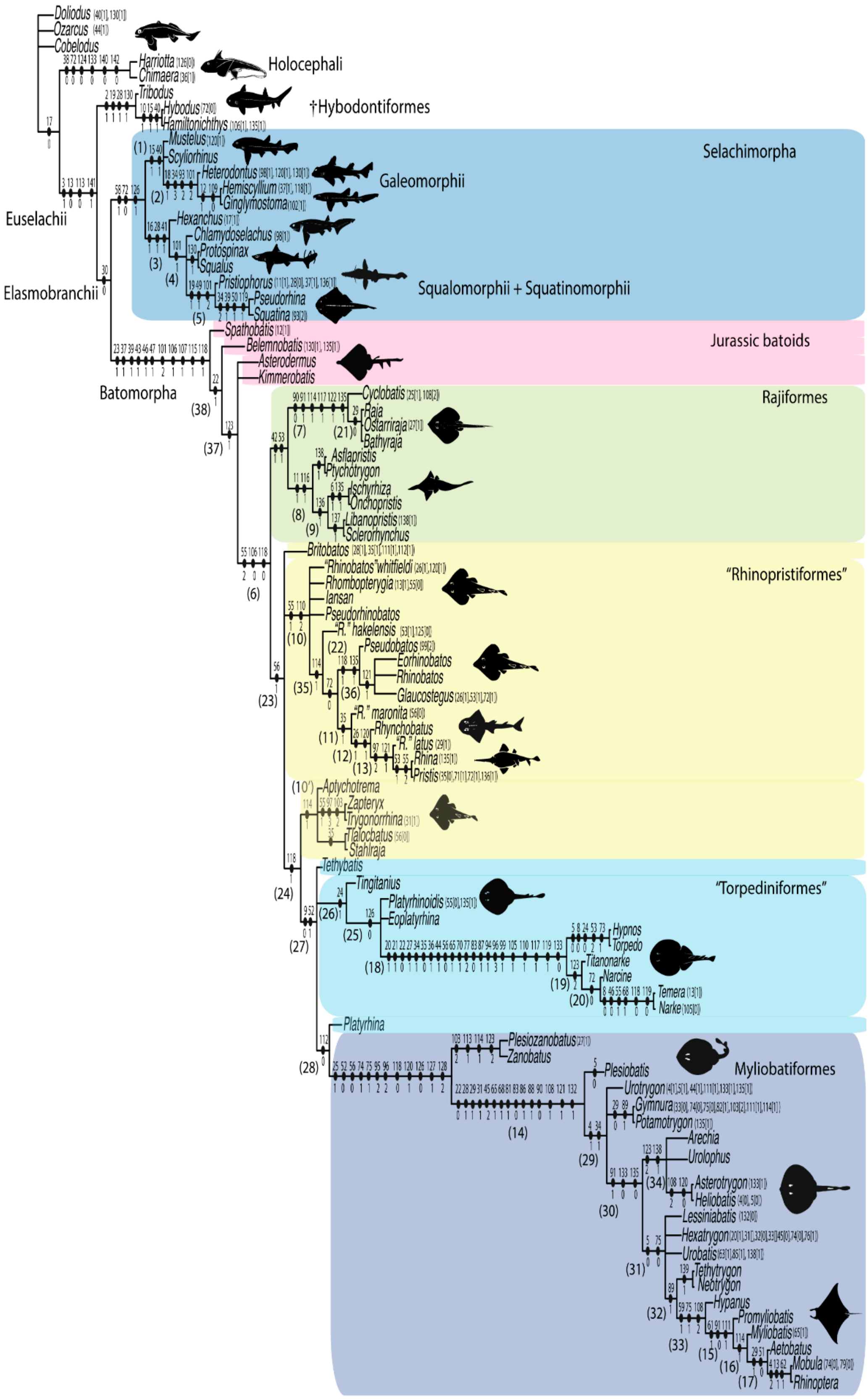

Figure S2. Strict consensus tree estimated from Maximum Likelihood analysis in PAUP, after the selection of the trees with the best scores. Clade numbers in parentheses.

Character discussion

1. **Upper eyelid:** (0) Present; (1) absent. Based on Aschliman et al. [1] (char. 1).

Parsimony tree reconstruction (Ptr) and maximum-likelihood tree reconstruction (MLtr): The presence of an upper eyelid is recovered as a basal feature for selachians and holocephalians. The loss of the upper eyelids is recovered a shared feature for the crown batomorphs (clade 6, Figure 1,2).

2. **Cephalic spines:** (0) Absent; (1) present. Based on Maisey [2,3].

Ptr and MLtr: The presence of cephalic spines is recovered as a synapomorphy for †Hybodontiformes. The absence of these spines is considered a basal feature, as is the state shared by symmoriids, holocephalians, selachians and batomorphs.

3 **Rostral cartilages:** (0) Arise from the medial area of the trabecula only; (1) medial area of the trabecula+lamina orbitonasalis.

Ptr and MLtr: Rostral cartilages that topologically seem to have arisen for the medial area of the trabecula in interaction with the lamina orbitonasalis is a synapomorphy for the Euselachian clade. The absence of these interactions in symmoriids and holocephalians is a common feature between these groups and the basal feature for the chondrichthyan tree.

4 **Rostral cartilage:** (0) Well-developed rostral plate with various degrees of contribution from the lamina orbitonasalis; (1) reaches the tip of the snout (carried by the growth of the pectoral fin); (2) reaches the tip of the snout (growth of lamina orbitonasalis to support the cephalic fins).

Ptr and MLtr: Well-developed rostral cartilages are a basal feature for Euselachian. Within batomorphs, there is the appearance of two additional states: 1) rostral cartilages located between the tip of the pectoral fins that reach the tip of the snout, which are a shared feature among stingrays being present in *Gymnura*, *Potamotrygon*, *Urotrygon*, *Urolophus*, †*Asterotrygon*; 2) growth of lamina orbitonasalis to support the cephalic fins, which is a synapomorphy for the (*Mobula*, *Rhinoptera*) clade (Figure 1).

MLtr: The rostral cartilages located between the tip of the pectoral fins, reaching the tip of the snout, are recovered as a synapomorphy of clade 29 (Figure 2) with an additional gain in *Urotrygon*.

5. **Medial growth of rostral cartilage:** (0) Inconspicuous; (1) conspicuous (noticeable). Modified from Aschliman et al. [1] (char. 26), Villalobos–Segura et al. [4] (char. 27) and Claeson et al. [5] (char. 1).

Ptr and MLtr: †*Doliodus* presents medial growth of the rostral cartilages placing this state as the basal feature, as symmoriids and holocephalians are coded as inapplicable, causing uncertainty in their basal states. There is a subsequent loss of growth in the (*Hypnos, Torpedo*) clade, with additional losses in the Myliobatiformes clade (absent in *Hexatrygon, Plesiobatis,* †*Heliobatis,* †*Lessiniabatis, Urobatis,* †*Tethytrygon, Neotrygon, Hypanus, Promyliobatis, Myliobatis, Aetobatus, Mobula, Rhinoptera*).

MLtr: The lack of rostral cartilages growth is an independent loss and a synapomorphy for the (*Hypnos, Torpedo*) clade and clade 31(Figure 2).

6. **Different morphologies of cartilage on rostrum (highly porous peripherically and fibrous at the central portion):** (0) Absent; (1) present.

Ptr and MLtr: The presence of a highly porous peripheral cartilage at the sides of the rostrum, and fibrous wood-like cartilage at the central portion pattern across the rostrum is currently restricted to †*Onchopristis* and †*Ischyrhiza* (Figures 1,2).

7 **Rostral processes:** (0) Absent; (1) present. Modified from Aschliman et al. [1] (char. 29); Villalobos–Segura et al. [4] (char. 32); Claeson et al. [5] (char. 49).

Ptr: The rostral processes are a synapomorphy of Torpediniformes.

MLtr: The rostral processes are a shared feature between *Platyrhina, Platyrhinoidis*, †*Eoplatyrhina*, *Hypnos*, *Torpedo*, *Narcine*, †*Titanonarke*, *Temera*, *Narke*.

8 **Rostral processes (proximal articulation):** (0) Articulated with nasal capsules; (1) continuous with neurocranium; (2) articulated with ventral aspect of rostral cartilage.

Ptr and MLtr: There is uncertainty in the basal state's reconstruction caused by the inapplicable symbol in the taxa with no rostral processes. The presence of an articulation between the ventral aspect of rostral cartilage and the rostral process is a basal feature for the Torpediniformes with subsequent gains of the rostral process's articulation with the nasal capsule in *Hypnos*, *Torpedo* and *Temera*. *Narcine* and †*Titanonarke* keep the basal state (articulation with the ventral aspect of the rostral cartilages) with a tripodal state of *Narke* (0 and 1) (Figures 1,2).

9 **Rostral appendix**: (0) Absent; (1) present. Modified from Aschliman et al. [1], char. 25.

Ptr: The presence of rostral appendices is a synapomorphy for the Rhinopristiformes with independent gains of clade 22 (Figure 2) and *Bathyraja* and *Raja*.

MLtr: The presence of rostral appendices is a shared feature of †*Spathobatis*, †*Asterodermus*, †*Kimmerobatis, Bathyraja, Raja,* †*Britobatos,* †*Rhinobatos whitfieldi,* †*Rhombopterygia,* †*Iansan,* †*Pseudorhinobatos,* †*Rhinobatos hakelensis, Pseudobatos, Rhinobatos, Glaucostegus,* †*Rhinobatos maronita, Rhynchobatus,* †*Rhinobatos latus, Rhina, Pristis, Aptychotrema,* †*Tlalocbatus,* †*Stahlraja, Zapteryx* and *Trygonorrhina.* The absence of rostral appendices is a synapomorphy of clade 25 (Figure 2).

10. **Caudal internasal keel**: (0) Absent; (1) present. Based on observations by Maisey [6] (Figure 3).

Ptr and MLtr: The presence of a caudal internasal keel (Figure 3) is a synapomorphy of the (†*Hybodus*, †*Hamiltonichthys*) clade.

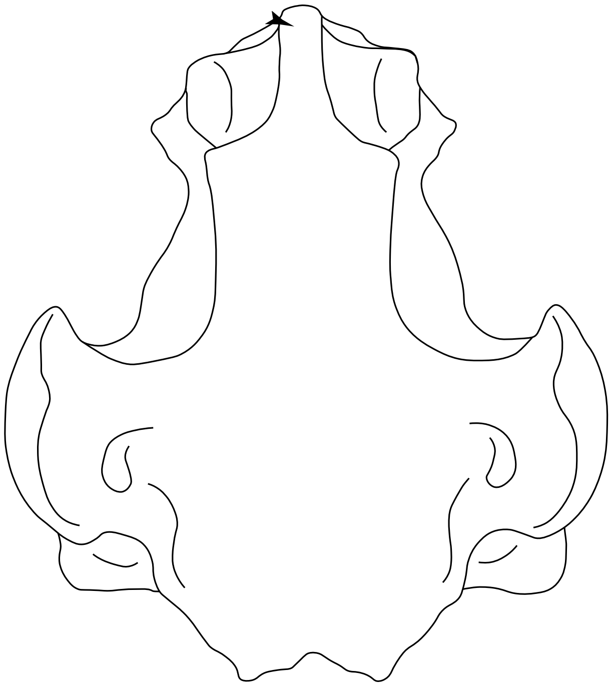

Figure S3. Interpretative drawings of the ventral view of †*Egertonodus* (†*Hybodus*) *basanus* after Maisey, [15] (Text-Figure 2B) (State 1). Arrowhead indicates the caudal internasal keel.

11. **Rostral passage of superficial ophthalmic nerve:** (0) Covered; (1) open.

Ptr and MLtr: An open rostral passage of the superficial ophthalmic nerve is a synapomorphy of the †Sclerorhynchoidei clade, with independent gains in *Pristiophorus* and *Torpedo*.

12. **Anterior preorbital foramen:** (0) Dorsally located; (1) anteriorly located. Modified from Aschliman et al. [1] (char. 35); Villalobos–Segura et al. [4] (char. 37).

Ptr and MLtr: An anterior preorbital foramen located anteriorly is a synapomorphy of Rhinopristiformes with an independent gain in the (*Hemiscyllium, Ginglymostoma*) clade, in clade 16 (Figure 1) and †*Spathobatis*.

MLtr: An anterior preorbital foramen located anteriorly is recovered as a synapomorphy of the (*Hemiscyllium, Ginglymostoma*) clade with independent gains in †*Spathobatis* and being the basal state in clades 10, 10′ and 14 (Figure 2).

13. **Preorbital process:** (0) Present; (1) absent. Taken from Aschliman et al. [1] (char. 33).

Ptr and MLtr: The presence of a postorbital process is a synapomorphy for the Euselachii clade, with subsequent independent losses in the (*Mobula, Rhinoptera*) clade and *Temera*, †*Rhombopterygia*, *Holocephali*, †*Doliodus*, †*Ozarcus* and †*Cobelodus*.

14. **Jaw support:** (0) Holostyly; (1) hyostyly; (2) archaeostylic. Based on Maisey [7] and Wilga and Ferry [8].

Ptr and MLtr: The archaeostylic state (sensu [9]) is the basal state for chondrichthyans, being present in †*Doliodus*, †*Ozarcus*, †*Cobelodus*. There

is uncertainty regarding the nodal state for holocephalians and euselachians, as holocephalians present a holostylic support as their basal state, while in euselachians it is hyostic.

15. **Ethmoidal articulation:** (0) Absent; (1) present. Based on Shirai [10] char11) and observations by Maisey [7,9], Lane and Maisey [11] and Wilga and Ferry [8].

Ptr and MLtr: An ethmoidal articulation is a synapomorphy, as independent gains of clade 15 (Figures 1,2) and the (†*Hybodus* and †*Hamiltonichthys*) clade.

16. **Orbital process articulation:** (0) Absent; (1) present. Based on observations by Maisey [7] and Klug [12] (char. 8). This character refers to the interaction between the palatoquadrate process located near the orbital and the neurocranium. †*Doliodus* and †*Cobelodus* kept the codification of Pradel et al. [13] (char. 26) (1). †*Ozarcus* coding follows Pradel et al.'s [14] observations.

Ptr and MLtr: An orbital process is a synapomorphy of clade 2 (Figures 1,2), with independent gains in †*Doliodus*, †*Cobelodus* and †*Ozarcus*.

17. **Postorbital articulation:** (0) Absent; (1–2) present. Modified from Klug [12] (char. 11), based on observations by Maisey [7].

Ptr and MLtre: The presence of a postorbital articulation located farther posteroventrolaterally of the chondrified lateral commissure is recovered as the basal state for chondrichthyans, being present in †*Doliodus*, †*Cobelodus* and †*Ozarcus*, with the subsequent gain of a postorbital articulation where the articulation surface is in the proximal part of the process in *Hexanchus*.

MLtre: The absence of a postorbital articulation is a synapomorphy of holocephalians and Euselachii, with the subsequent gain of a postorbital articulation state observed in *Hexanchus*.

18. **Downturned ethmoidal articulation:** (0) Absent; (1) present. Based on de Carvalho [15] (char. 4), Klug [12] (char. 4).

Ptr and MLtr: The presence of a downturn of the ethmoidal articulation postorbital articulation is a synapomorphy of clade 2 (Figures 1,2).

19. **Quadrate flange:** (0) Absent; (1) present. Modified from Maisey et al. [16] (char. 6).

Ptr and MLtr: The presence of this ledge process in the palatoquadrate is an independent gain and shared feature for the Hybodontiformes and clade 5 (Figure 1,2).

20. **Supraorbital crest:** (0) Present; (1) absent. Taken from Aschliman et al. [1] (char. 34). *Hexatrygon* coding follows Heemstra and Smith [17] observations.

Ptr and MLtr: The lack of a supraorbital crest is a synapomorphy of clade 16 (Figure 1,2), with an independent gain in *Hexatrygon*.

21. **Branchial electric organs:** (0) Absent; (1) present. Taken from Aschliman et al. [1] (char. 86).

Ptr and MLtr: The presence of branchial electric organs is a synapomorphy of clade 16 (Figures 1,2).

22. **Jugal arch:** (0) Absent; (1) present. Taken from Aschliman et al. [1] (char. 39).

Ptr and MLtr: The lack of a jugal arch is the basal state for chondrichthyans, as it is lost independently in †*Doliodus*, †*Ozarcus*, †*Cobelodus*, holocephalians, hybodonts and selachians. Within batomorphs, the gain of the jugal arch is the basal state, with independent losses in clades 14 and 18 (Figures 1,2).

23. **Antorbital cartilages:** (0) Absent; (1) present. Modified from Aschliman et al. [1] (char. 8).

Ptr and MLtr: The presence of antorbital cartilages is recovered as a synapomorphy of batomorphs.

24. **Antorbital cartilage (shape):** (0) Triangular-shaped with regular outline; (1) variously shaped and with an irregular outline.

Ptr: Irregular-shaped antorbital cartilages are a synapomorphy with independent gains in clades 25 and 19 (Figure 1).

MLtr: The presence of irregular-shaped antorbital cartilages is a synapomorphy of clade 26 (Figure 2).

25. **Antorbital cartilages (with regular outline):** (0) Well-developed; (1) reduced. Modified from Villalobos-Segura et al. [4] (char. 9), separated into two characters, 25 and 26.

Ptr and MLtr: The presence of small antorbital cartilages is a synapomorphy for the Myliobatiformes clade, with an independent gain in †*Cyclobatis*.

26. **Anterior process of antorbital cartilage (if regular outline):** (0) Absent; (1) present. Modified from Villalobos-Segura et al. [4] (char. 9). This character includes the variation observed in the anterior portion of the antorbital cartilage of batomorphs.

Ptr and MLtr: The presence an anterior process in the antorbital cartilages is a synapomorphy in clade 16 (Figures 1,2) with independent gains in *Platyrhina*, "†*Rhinobatos*" *whitfieldi* and *Zanobatus*.

27. **Postorbital process:** (0) Well-developed; (1) reduced. Based on Claeson et al. [5] (char. 12). This process could not be observed in †*Rhombopterygia*, †*Ischyrhiza* and †*Lessiniabatis* (?).

Ptr and MLtr: A reduced postorbital process is recovered (Figure 17I), a synapomorphy of clade 16 (Figures 1,2) with independent gains in †*Plesiozanobatus*, †*Ostarriraja*, *Chimaera*, *Harriotta* and †*Ozarcus*.

28. **Postorbital process:** (0) Narrow; (1) broad and-shelf like. Taken from Aschliman et al. [1], (char. 36).

Ptr and MLtree: A broad shelf-like postorbital process is independently a gain and a synapomorphy for the Hybodontiformes and clade 14 (Figures 1,2), and with an additional independent gain as the basal feature in clade 3 (Figures 1,2) and in †*Britobatos* as an autapomorphy.

MLtree: A broad postorbital process is a synapomorphy in clade 3 (Figure 2); consequently, the narrow state in *Pristiophorus* is interpreted as an independent gain and an autapomorphy.

29. **<u>Postorbital process</u>:** (0) Separated from triangular process; (1) fused with triangular process. Taken from Aschliman et al. [1] (char. 37).

Ptr and MLtr: The fusion between the triangular process and the postorbital process is recovered as the basal state for the chondrichthyan tree, being present in the hybodont and selachian clades. Within batomorphs, the nodal sate is also the fusion between these processes. There is a subsequent gain of the unfused state as a synapomorphy for clade 27 (Figures 1,2).

Ptr: Recovers independent gains of the fused state within the Myliobatiformes in the †*Arechia*, *Urolophus* and in clade 17 (Figure 1).

MLtr: The unfused sate is the basal feature for clade 27 (Figure 2), with an additional gain of this state as a synapomorphy for the (*Gymnura*, *Potamotrygon*) clade; there are independent gains of the fused state within the Myliobatiformes in clades 14 and 17 (Figure 2).

30. **<u>Postorbital process:</u>** (0) Projects laterally; (1) projects ventrolaterally. Taken from Aschliman et al. [1] (char. 38).

Ptr and MLtr: The ventrolateral projection of the postorbital process is recovered as the basal feature of chondrichthyans, being present in †*Cobelodus*, †*Doliodus* and the hybodont clade. The lateral projection of the postorbital process is a synapomorphy for the Elasmobranchii clade, and within it, there is a subsequent gain of the ventrolateral projection state as a shared feature in clade 16 (Figures 1,2).

31. **<u>Anterior nasal lobe-mouth</u>:** (0) Fails to reach the mouth; (1) reaches the mouth. Modified from Aschliman et al. [1] (char. 11), separated into two characters, 31 and 32. Coding of *Hexatrygon* follows Heemstra and Smith, [17] (Text-Figures 3, 4, 6) observations.

Ptr and MLtr: There is uncertainty regarding the basal state for the tree, as there are several fossil taxa with a not-determined state. The revision of the extant material places an anterior nasal lobe not reaching the mouth as the basal state for chondrichthyans. The presence of an anterior nasal lobe reaching the mouth is a shared feature between *Raja*, *Bathyraja*, *Torpedo*, *Narke*, *Temera*, *Trygonorrhina*, *Urolophus*, *Urobatis*, *Urotrygon*, *Plesiobatis*, *Hypanus*, *Potamotrygon*, *Neotrygon*, *Gymnura*, *Myliobatis*, *Aetobatus*, *Rhinoptera* and *Mobula*.

MLtr: A short anterior nasal lobe is the basal state for the Myliobatiformes, and within them there is a subsequent gain of the extension of the nasal lobe as a synapomorphy of clade 14.

32. **<u>Anterior nasal lobe</u>:** (0) Fails to cover most of the medial half of the naris; (1) covers more than the medial half of the naris.

Ptr: An anterior nasal lobe covering more than the medial half of the naris is a synapomorphy of clade 26 (Figure 2), with independent gains in *Zapteryx*, *Trygonorrhina*, *Rhina* and *Pristis*.

MLtr: An anterior nasal lobe covering more than the medial half of the naris, is the basal feature of clade 25, with independent gains in *Zapteryx*, *Trygonorrhina*, *Rhina*, *Pristis*, *Raja* and *Bathyraja*.

33. **<u>Nasal curtain fringes</u>:** (0) Absent; (1) present (New).

Ptr and MLtre: The use of reductive coding produces uncertainties in the basal state's reconstruction for this character. Fleshy fringes in the nasal curtain are present in *Raja, Bathyraja, Hypanus, Urobatis, Neotrygon, Urolophus Potamotrygon, Plesiobatis, Urotrygon, Myliobatis, Aetobatus, Mobula* and *Rhinoptera*. A nasal curtain without fringes is present in *Gymnura, Hexatrygon, Torpedo, Hypnos, Narcine, Narke, Temera* and *Trygonorrhina*. The wide distribution of these states among batomorphs possibly represents a convergence (Figures 1,2).

34. **Nasal capsules:** (0) Laterally expanded; (1) ventrolaterally expanded; (2) anterolaterally expanded; (3) prolonged interorbitonasal region, which forms a pedicel "trumpet-shaped nasal capsule".

Ptr and MLtr: Laterally expanded nasal capsules are the basal state for chondrichthyans, with the subsequent gain of trumpet-shaped nasal capsules as a synapomorphy of clade 2 (Figures 1,2); the anterolateral expansion of the nasal capsules is a synapomorphy of (†*Pseudorhina, Squatina*); and the ventrolateral expansion of the nasal capsules is a synapomorphy for clade 18 (Figures 1,2).

Ptr: There is an independent gain of the ventrolateral expansion of the nasal capsules, as the basal feature of clade 14 (Figure 1), except for *Hexatrygon* and *Plesiobatis*, which present the basal state.

MLtr: Presents a similar-state reconstruction for this feature. However, the additional gain of the ventrolateral expansion of the nasal capsules is a synapomorphy of clade 29 (Figure 2).

35. **Nasal capsule margin:** (0) Straight; (1) horn-like process. Taken from Villalobos-Segura et al. [4] (char. 83).

Ptr: The presence of a horn-like process is a synapomorphy of clade 11 (Figures 1,2) and clade 24 (Figure 1). There are independent gains of the horn process in †*Tlalocbatus*, †*Stahlraja* and †*Britobatos*.

MLtr: Presents a similar reconstruction for this character as the parsimony tree, but in this case, the paraphyletic state of the thornback clades makes the recovery of this feature as a synapomorphy for the group impossible.

36. **Nostrils:** (0) Separated; (1) close together (New).

Ptr and MLtre: A reduced internarinal space is recovered as a synapomorphy of clade 18 (Figures 1,2) with an independent gain in *Chimaera*.

37. **Basibranchial:** (0) Segmented; (1) unsegmented (New). According to Shirai [10], *Pristiophorus* presents an unsegmented basibranchial.

Ptr and MLtre: An unsegmented basibranchial is recovered as a synapomorphy for batomorphs with independent gains in *Pristiophorus* and *Hemiscyllium*.

38. **Hypobranchials direction:** (0) Not directed towards midline; (1) midline-directed. Taken from Maisey [3] (char. 13).

Ptr and MLtr: The lateral direction of the hypobranchials (Figure 4A) is a synapomorphy of holocephalians. The midline body direction of hypobranchials (Figure 4B) is the basal state for the chondrichthyans.

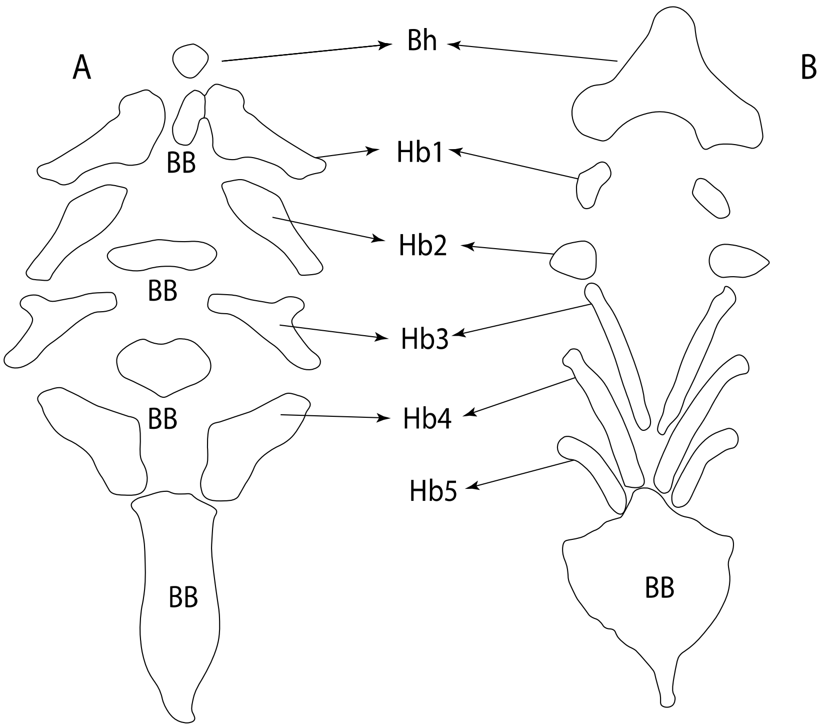

Figure S4. Interpretative drawings of ventral view of basibranchial and hypobranchials. State (0): A, *Callorhinchus capensis* (ANSP 174852) after de Carvalho et al. [18] (Text-Figure 9A). State (1): B, *Hemiscyllium ocellatum* (AMNH 38151) after de Carvalho et al. [18] (Text-Figure 9E) Abbreviations: BB, basibranchial; Bh, basihyal; Hb1–5, hypobranchials.

39. **Fourth hypobranchial:** (0) Well-developed; (1) reduced (New).

Ptr and MLtr: A well-developed fourth hypobranchial is the basal state for chondrichthyans. The reduction of the fourth hypobranchial is a synapomorphy of batomorphs (Figures 1,2).

40. **Suborbital shelf:** (0) Absent; (1) present. Based on Shirai [10,19] and Klug's [12] observations.

Ptr and MLtr: The presence of a suborbital process is a synapomorphy for the Galeomorphii clade, with independent gains in the (†*Hybodus*, †*Hamiltonichthys*) clade and †*Doliodus*.

41. **Basitrabecular process:** (0) Absent; (1) present. Taken from Shirai [10] (char. 13) and de Carvalho's and Maisey's [20] (char. 21) and Klug [18] (char. 10).

Ptr and MLtre: The presence of a basitrabecular process is a synapomorphy for clade 3 (Figures 1,2).

42. **Hypobranchials-basibranchial:** (0) Articulated with the basibranchial; (1) fused. Based on observations by Miyake [21].

Ptr and MLtr: The lack of an articulation surface between the basibranchial and hypobranchial (Figure 5A–B) is a synapomorphy for the Rajiformes, with an independent gain in the Myliobatiformes (except for *Zanobatus*, *Urobatis*, *Hexatrygon* and *Urotrygon*) (Figures 1,2).

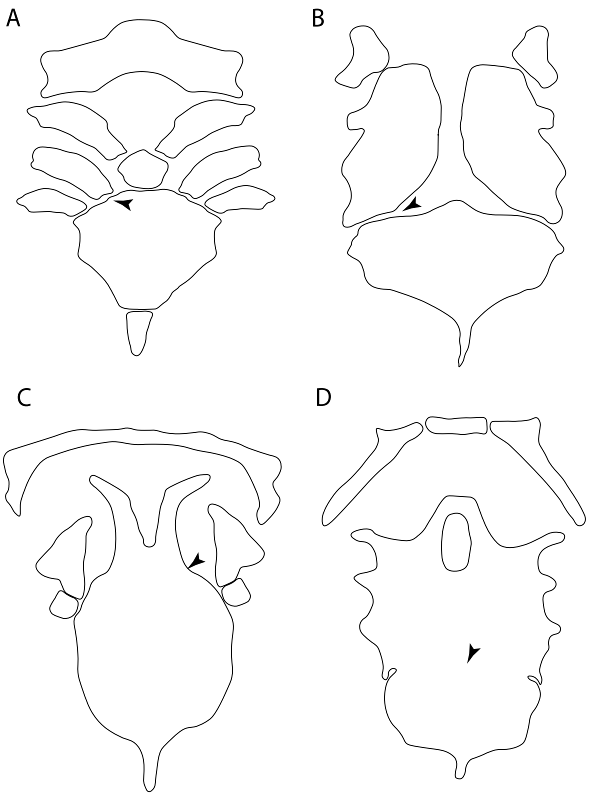

Figure S5 Interpretative drawings of the branchial skeletal elements in ventral view (basihyal, hypobranchials and basibranchial). State (0): A, *Heterodontus zebra* (HUMZ 37666) after Shirai [10] (plate 32 D); B, *Narcine tasmaniensis.* (NHMUK 1961). State (1): C, *Raja pulchra* (HUMZ 92011) after, Nishida [22] (Text-Figure 28 E); D, *Gymnura micrura* (FMNH 89990) after Lovejoy [23] (Text-Figure 7E). Arrowhead: union between the basibranchial and hypobranchial.

43. **Last ceratobranchial:** (0) Free; (1) articulated distally with the scapulacoracoid. Taken from Aschliman et al. [1] (char. 4).

Ptr and MLtr: The articulations between the last ceratobranchial and the scapulacoracoid are a synapomorphy for the batomorphs.

44. **Basihyal:** (0) Present; (1) absent. Modified from Aschliman et al. [1] (char. 48); Villalobos-Segura et al. [4] (char. 48) and Claeson et al. [5] (char. 27). Previous analyses placed both basihyal and first hypobranchial together in a single character, resulting in a mix of neomorphic and transformational characters. We therefore propose independence among these structures, providing separate characters for their presence/absence and interaction (char. 45–46).

Ptr and MLtr: The lack of a basihyal is a synapomorphy of clade 18 (Figure 1,2) with independent losses in †*Ozarcus*, *Myliobatis*, *Aetobatus*, *Rhinoptera* and *Mobula*.

45. **First hypobranchial-basihyal:** (0) Separated; (1) fused; (2) segmented.

Ptr and MLtr: The separation between the first hypobranchial and the basihyal is the basal stage for the chondrichthyan tree, with the subsequent gain of the fused state in the crown batomorphs group (clade 6, Figures 1,2) and the successive gain of the segmented basihyal separated from the first hypobranchial sate in the Myliobatiformes, being

present in †*Asterotrygon*, *Urolophus*, *Urobatis*, *Plesiobatis*, *Hypanus*, *Potamotrygon*, *Neotrygon* and *Gymnura*.

46. **<u>Ceratohyal</u>:** (0) Well-developed; (1) reduced. Taken from Aschliman et al. [1] (char. 49).

Ptr and MLtr: The ceratohyal cartilage is a synapomorphy for all batomorphs, except for *Narke* and *Temera*, which present an independent gain of the basal state for the chondrichthyan tree (a well-developed ceratohyal).

47. **<u>Pseudohyal</u>:** (0) Absent; (1) present. Taken from Aschliman et al. [1] (char. 3).

Ptr and MLtr: The presence of a pseudohyal is a synapomorphy of the batomorphs.

48. **<u>Cervical vertebral</u>:** (0) Unmodified; (1) synarcual, product of expansion of vertebra centra; (2) fusion of neural/basidorsal and hemal/basiventral elements. Modified from Aschliman et al. [1] (char. 5) to include Johanson et al. [24] observations.

Ptr and MLtr: There is uncertainty in reconstructing the basal state, as †*Doliodus*, symmoriids, and hybodonts lack calcified vertebra centra. The presence of a "synarcual" composed of the neural/basidorsal and hemal/basiventral elements is a shared feature of the holocephalian clade. The presence of not-fused vertebra centra is a shared feature among selachians. A synarcual characterized by the fusion of the cervicothoracic vertebral centra is a shared feature of batomorphs.

49. **<u>Expanded basiventral process of cervical vertebrae</u>:** (0) Absent; (1) present. Taken from Maisey et al. [16] (chars. 16–18).

Ptr: The presence expanded basiventral processes in their cervical vertebrae, in which the first process is larger than the other ones, and their size reduction posteriorly is a synapomorphy of clade 5 (Figures 1,2).

50. **<u>Occipital hemicentrum</u>:** (0) Absent; (1) present. Modified from Shirai [10] (char. 21); and Klug [12] (char. 16).

Ptr and MLtr: This character is a synapomorphy of the (†*Pseudorhina* and *Squatina*) clade.

51. **<u>Lateral stays</u>:** (0) Fused distally with medial crest; (1) free of medial crest (New). Taxa with no synarcual (i.e., outgroups) or with no lateral stays on cervicothoracic synarcual (i.e., *Chimaera* and *Harriotta*) are coded as inapplicable (-), which makes the reconstruction of this character for the basal chondrichthyan in the trees (Figures 1,2) impossible.

Ptr and MLtr: The presence of lateral stays not fused with the medial crest of the synarcual is the basal feature of batomorphs, with the subsequent gain of the fuse state as a synapomorphy of clade 17 (Figures 1,2).

52. **<u>Orientation of lateral stays:</u>** (0) Dorsally directed; (1) laterally directed. Modified from Villalobos-Segura et al. [4] (char. 53).

Ptr: The lateral direction of the lateral stays is a synapomorphy for the Torpediniformes. In contrast, the dorsal direction is the common state for the reaming batomorphs.

MLtr: The lateral direction of the lateral stays is a synapomorphy for clade 27 (Figure 2). There is a subsequent independent gain of the common state for the batomorphs (dorsally directed) as a synapomorphy for the Myliobatiformes.

53. **Ventral occipital-synarcual articulation:** (0) Synarcual lip firmly fitted into notch in basicranium; (1) synarcual lip rests in foramen magnum; (2) synarcual lip reduced, with a paired connection into notch in basicranium. Taken from Aschliman et al. [1] (char. 52).

Ptr and MLtr: The synarcual lip firmly fitted into the notch in the basicranium (Figure 6A–B) is recovered as the basal state for batomorphs, with this state being present across several groups and taxa (*Narcine*, *Narke*, *Temera*, *Platyrhinoidis*, †*Tingitanius*, *Platyrhina*, †*Asterodermus*, †*Spathobatis*, †*Belemnobatis*, "†*Rhinobatos*" *maronita*, "†*R*". *latus*, †*Stahlraja*, *Rhynchobatus*, *Rhinobatos*, *Pseudobatos*, †*Eorhinobatos*, †*Pseudorhinobatos*, *Trygonorrhina*, *Zapteryx*, *Aptychotrema*, †*Britobatos*, †*Iansan* and myliobatiforms). The synarcual lip resting firmly inside the foramen magnum is a synapomorphy of the Rajiformes clade. This state has independent gains in the (*Pristis*, *Rhina*) clade and *Glaucostegus* (Figure 6C–D). A reduced synarcual lip with a paired connection to the notch of the basicranium (Figure 6E–F) is recovered as a synapomorphy of the (*Torpedo*, *Hypnos*) clade.

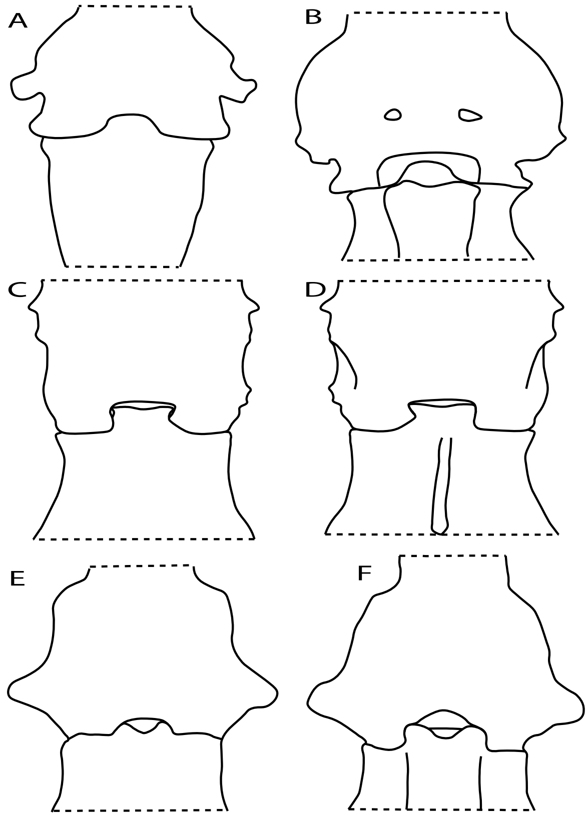

Figure S6 Interpretative drawings of ventral and dorsal view of the synarcual and neurocranium. State (0): A–B, *Narcine brasiliensis* (AMNH 77069 https://sharksrays.org/). State (1): C–D, *Pristis sp.* (NHMUK 2147483647). State (2): E–F, *Hypnos monopterygius* (USNM 84374 https://sharksrays.org/).

54. **Arcualia dorsalis:** (0) Absent; (1) present. Taken from Brito et al. [25] (char. 30).

Ptr: The presence of arcualia dorsalis is a synapomorphy of clade 10′ (Figure 1) with an independent gain in *Platyrhina* and *Platyrhinoidis*.

MLtr: The presence of arcualia dorsalis is a shared feature among *Aptychotrema, †Tlalocbatus, †Stahlraja, Zapteryx, Trygonorrhina, Platyrhina* and *Platyrhinoidis*.

55. **Position of vertebral centra in the synarcual relative to position of suprascapula:** (0) Present through entire length near the articulation with the neurocranium; (1) reaching rostral to the suprascapula; (2) reaching caudal to the suprascapula. Taken from Marramà et al. [26] (char. 78).

Ptr: The vertebral centra reaching caudally to the suprascapula is the basal state for batomorphs being present across several groups and taxa (*Raja, Bathyraja, †Cyclobatis, †Titanonarke, Torpedo, Hypnos, Narcine, Platyrhina, †Eoplatyrhina, †Tingitanius, Pristis, Rhina, Aptychotrema, †Britobatos, Zanobatus, Urolophus, Urobatis, Urotrygon, Plesiobatis, Hypanus, Potamotrygon, Neotrygon, Gymnura, Myliobatis, Aetobatus, Rhinoptera,* Mobula, *†Ptychotrygon, †Sclerorhynchus, †Libanopristis, †Asflapristis, †Promyliobatis* and *†Onchopristis*). The presence of vertebral centra across the entire synarcual length reaching the articulation with the cranium is a shared feature between *Platyrhinoidis, †Asterodermus, †Spathobatis* and *†Belemnobatis*. The vertebral centra reaching rostrally to the scapula is recovered as a shared feature among *Narke, Temera, "†Rhinobatos" maronita, "†R." latus, Rhynchobatus, Glaucostegus, Rhinobatos, Pseudobatos, †Pseudorhinobatos, Trygonorrhina, Zapteryx* and *†Iansan*.

MLtr: The paraphyletic Rhinopristiformes clades recover state (1) with two independent origins as a synapomorphy of clades 10 and (*Trygonorrhina* and *Zapteryx)* (Figure 1,2).

56. **Differentiated lateral uvulae on teeth:** (0) Absent; (1) present. Taken from Claeson et al. [5] (char. 22).

Ptr: The presence of lateral uvulae on the lingual face of their teeth is a synapomorphy of Rhinopristiformes with independent gains in *Platyrhina, Platyrhinoidis* and *†Tingitanius*.

MLtr: The lack of lateral uvulae on teeth is recovered the basal feature among the chondrichthyan tree and consequently for batomorphs. The presence of lateral uvulae is the basal feature for clade 23 (Figure 2), with either retention or independent gains of the absent state as a synapomorphy of clade 18 (Figure 2) and for the Myliobatiformes.

57. **Osteodentine:** (0) Absent; (1) present. Modified from Aschliman et al. [1] (char. 19). This character is coded as binary instead of multistate.

Ptr and MLtr: The wide distribution of the lack of osteodentine in the tooth roots among chondrichthyans causes uncertainty in the basal-state reconstruction for the whole tree. The lack of osteodentine is the basal state in batomorphs. The presence of osteodentine is gained several times, being present in *Chlamydoselachus, Hexanchus, Squatina, Squalus, Ginglymostoma, Hemiscyllium, Raja, Bathyraja, Tethytrygon, Neotrygon, Hypanus, †Promyliobatis, Myliobatis, Aetobatus, Mobula, Rhinoptera*.

58. **Three-layered enameloid structure:** (0) Absent; (1) present. Based on observations by Maisey [2,3] and Reif, [27].

Ptr and MLtr: The presence of a tooth enameloid is a synapomorphy of the Selachimorpha, with an independent loss in the large crushing lateral teeth of *Heterodontus*. Its absence in batomorphs, according to the present results, is a primitive feature.

59. **Pulp cavities in tooth roots:** (0) Present; (1) absent.

Ptr: The lack of a pulp cavity is a shared feature for *Hypanus*, *Myliobatis*, *Aetobatus* and *Rhinoptera*.

MLtr: The lack of a pulp cavity is a synapomorphy of clade 33 (Figure 2).

60. **Pelvic basipterygium:** (0) Fused to first radial; (1) separated from first radial. Based on observations by Riley et al. [28].

Ptr and MLtr: There is uncertainty regarding the basal state for the chondrichthyan tree, with *Chimaera* and *Harriotta* presenting a single element supporting their pelvic fin rays, and the state for †*Doliodus*, †*Ozarcus* and †*Cobelodus* being unknown. The separation between the first radial and the basipterygium is the basal feature for the Euselachi.

61. **Cephalic lobes:** (0) Absent; (1) present. Modified from Aschliman et al. [1] (char. 10), separated into two characters, 61 and 62.

Ptr and MLtr: The presence of cephalic lobes is a synapomorphy of clade 15 (Figures 1,2).

62. **Cephalic lobes (number):** (0) Single lobe; (1) two lobes.

Ptr and MLtr: The mesial contact of most anterior of the propterygia radials of both pectoral fins forming a single lobe anterior to nasal capsules is the basal state for pelagic stingrays, being present in †*Promyliobatis*, *Myliobatis* and *Aetobatus*, with the subsequent gain of the separation of the cephalic lobe state (i.e., two cephalic fins) as a synapomorphy of the (*Rhinoptera*, Mobula) clade.

63 **Spiracular tentacle:** (0) Absent; (1) present. Taken from Aschliman et al. [1] (char. 12).

Ptr and MLtr: The presence of a tentacle in the inner margins of the spiracle is an independent gain between *Urobatis* and *Urotrygon*.

64. **Ligamentous sling on Meckel's cartilage:** (0) Absent; (1) present. Taken from Aschliman et al. [1] (char. 83).

Ptr and MLtr: The absence of a ligamentous sling at the symphysis of the Meckel's cartilage that supports the intermadibularis, coracomandibularis and depressor mandibularis muscles is the basal feature for the chondrichthyan clade, with the subsequent gain of the ligamentous sling as a shared feature between *Narcine*, *Narke* and *Temera*

65. **Spiracularis:** (0) Undivided; (1) divided. Taken from Aschliman et al. [1] (char. 85).

Ptr and MLtr: The presence of a divided spiracularis is a synapomorphy and independent gains of clades 14 and 18 (Figures 1,2).

66. **<u>Spiracularis (if divided)</u>:** (0) One bundle enters the dorsal oral membrane underlying the neurocranium; (1) spiracularis splits into lateral and medial bundles, with the medial bundles inserting onto the posterior surface of the Meckel's cartilage and the lateral bundle onto the dorsal edge of the hyomandibula; (2) spiracularis subdivided proximally and inserts separately into the palatoquadrate and the hyomandibula.

Ptr and MLtr: A divided spiracularis in which one muscle bundle enters the dorsal oral membrane underlying the neurocranium is a shared feature in *Torpedo, Hypnos, Narcine, Narke* and *Temera*. The spiracularis split into lateral and medial bundles, with the medial bundles inserted onto the posterior surface of the Meckel's cartilage and the lateral bundle onto the dorsal edge of the hyomandibula is a shared feature in *Urolophus, Urobatis, Urotrygon, Plesiobatis, Hypanus, Potamotrygon* and *Neotrygon*. There is a subsequent gain of the spiracularis subdivided proximally, inserting separately into the palatoquadrate and the hyomandibula state in *Rhinoptera*.

67. **<u>Coracobranchialis</u>:** (0) Consists of three to five components; (1) single component. Taken from Aschliman et al. [1] (char. 87).

Ptr and MLtr: A coracobrachialis consisting of multiple components is recovered as the basal state for the chondrichthyan tree, with the subsequent gain of the single-element state as a shared feature of clade 20 (Figures 1,2).

68. **<u>Coracohyomandibularis</u>:** (0) Single origin; (1) separate origins. Modified from Aschliman et al. [1] (char. 88). The character is split into two (68 and 69), aiming to increase the grouping information on the separate origins of the coracohyomandibularis.

Ptr: A single origin of the coracomandibularis is the basal state of the chondrichthyan tree. There is a subsequent gain of the separate origin state as a synapomorphy for the (*Narke, Temera*) clade and an independent gain in Myliobatiformes being present in *Urolophus, Urobatis, Urotrygon, Plesiobatis, Hypanus, Potamotrygon, Neotrygon, Gymnura, Myliobatis, Aetobatus, Rhinoptera* and *Mobula*.

MLtr: Recovers a similar character reconstruction as the parsimony tree. However, the more resolved topology of the Myliobatiformes places also the separate origins as a synapomorphy of clade 14 (Figure 2).

69. **<u>Coracohyomandibularis (if separate origins)</u>:** (0) Originates in the facia supporting the insertion of the coracoarcualis and on the pericardial membrane; (1) originates on the anterior portion of the ventral gill arch region and on the pericardial membrane.

Ptr and MLtr: A coracohyomandibularis that originates in the facia supporting the insertion of the coracoarcualis and on the pericardial membrane is a shared feature of *Narke, Temera*. The coracohyomandibularis originating on the anterior portion of the ventral gill arch region and the pericardial membrane is a shared feature of *Urolophus, Urobatis, Urotrygon, Plesiobatis, Hypanus, Potamotrygon, Neotrygon, Gymnura, Myliobatis, Aetobatus, Rhinoptera* and *Mobula*, and possibly a synapomorphy of Myliobatiformes if the fossil taxa are deactivated.

70. **<u>Coracohyoideus</u>:** (0) Present; (1) absent. Modified from Aschliman et al. [1] (char. 89). The character is split into two, 70 and 71, aiming to increase the grouping information.

Ptr and MLtr: The basal state for chondrichthyans is the presence of a coracohyoideus. The lack of this muscle is a synapomorphy of clade 18 (Figure 1,2).

71. **<u>Coracohyoideus (if present)</u>:** (0) Parallel to body axis; (1) runs parallel to the body axis and is very short; (2) runs diagonally from the wall of the first two gill slits to the posteromedial aspect of the basihyal or first basibranchial; (3) each muscle fuses with its antimere at a raphe near its insertion on the first hypobranchial.

Ptr: A coracohyoideus parallel to the body axis is recovered as the basal state for the chondrichthyan tree, being present across several groups and taxa (*Chimaera, Harriotta, Chlamydoselachus, Hexanchus, Heterodontus, Squatina, Pristiphoridae, Squalus, Ginglymostoma, Raja, Bathyraja, Rhynchobatus, Glaucostegus, Rhina, Rhinobatos, Pseudobatos, Trygonorrhina* and *Zapteryx*). A very short coracohyoideus that runs parallel to the body axis is an autapomorphy of *Pristis*. A coracohyoideus running diagonally from the wall of the first two gill slits to the posteromedial aspect of the basihyal or first basibranchial is a synapomorphy of clade 27 (Figure 1), with the subsequent gain of the coracohyoideus fusing with its antimere at a raphe near its insertion on the first hypobranchial gain as a shared state in clade 16 (Figure 1).

MLtr: Presents a similar reconstruction for this character as the parsimony tree. The diagonal arrangement of the coracohyoideus from the wall of the first two gill slits to the posteromedial aspect of the basihyal is not a synapomorphy due to the polytomic state of the thornbacks within clade 27 (Figure 2).

72. **<u>Labial cartilages</u>:** (0) Present; (1) absent. Taken from Aschliman et al. [1] (char. 43).

Ptr and MLtr: The absence of labial cartilages is the basal state for the chondrichthyan tree, being a shared feature across several taxa of different Chondrichthyan groups (e.g., †*Doliodus*, †*Cobelodus*, †*Ozarcus*, †*Hamiltonichthys*, †*Tribodus*, *Raja*, *Bathyraja*, †*Cyclobatis*, †*Titanonarke*, *Torpedo*, *Glaucostegus*, *Hypnos*, *Platyrhina*, *Platyrhinoidis*, †*Tingitanius* †*Kimmerobatis*, †*Asterodermus*, †*Spathobatis*, †*Stahlraja*, *Pristis*, *Trygonorrhina*, *Zapteryx Aptychotrema*, living Myliobatiformes, †*Iansan*, †*Ostarriraja*, †*Arechia*, †*Lessiniabatis* and †*Tethytrygon*). Independent gains of labial cartilages are recovered as synapomorphies of the Holocephali, Selachimorpha and clade 20 (Figures 1,2) and an autapomorphy of †*Hybodus*. The absence of labial cartilages is the basal state for batomorphs. However, their presence is widely distributed across several batomorphs groups and taxa ("†*Rhinobatos*" *maronita*, "†*R.*" *latus*, *Rhynchobatus*, *Rhina*, *Rhinobatos*, *Pseudobatos* and *Zanobatus*), which could suggest independent gains within these groups.

73. **<u>Medial section of hyomandibula</u>:** (0) Narrow; (1) expanded. Taken from Aschliman et al. [1] (char. 44).

Ptr and MLtr: A narrow hyomandibula is the basal feature of the chondrichthyan tree, with a subsequent gain of the expansion of the medial section as a synapomorphy of the (*Torpedo, Hypnos*) clade.

74. **<u>Hyomandibula-Meckelian ligament</u>:** (0) Absent; (1) present. Taken from Aschliman et al. [1] (char. 45).

Ptr and MLtr: The direct articulation between the hyomandibula and the Meckel's cartilage is the basal stage for the chondrichthyan tree. The distal tip of the hyomandibula joined to Meckel's cartilage by a long ligament is a synapomorphy of Myliobatiformes, with independent losses of the ligament in *Gymnura*, *Hexatrygon* and *Mobula*.

75. **<u>Small cartilages associated with hyomandibular-Meckelian ligament</u>:** (0) Absent; (1) present. Taken from Aschliman et al. [1] (char. 44).

Ptr and MLtr: The lack of cartilages between the hyomandibula and Meckel's cartilage (associated with hyomandibular-Meckelian ligament) is the basal state for the chondrichthyan tree. These small cartilages are recovered as a synapomorphy of Myliobatiformes.

MLtr: The more resolved topology for Myliobatiformes recovers a subsequent loss of these cartilages for clade 31 (Figure 2), followed by another gain of the cartilages in clade 33.

76. **<u>Depressor mandibularis</u>:** (0) Present; (1) absent. Taken from Aschliman et al. [1] (char. 84). The coding of *Hexatrygon* is based on McEachran et al. [29].

Ptr and MLtr: The presence of a depressor mandibularis is the basal feature for the chondrichthyan tree, with the subsequent loss of the muscle in clade 16 (Figure 1,2).

77. **<u>Intermandibularis</u>:** (0) Present; (1) absent; (2) modified as a narrow band of muscle that originates on the hyomandibula and inserts on the posterior margin of Meckel's cartilage. Taken from Aschliman et al. [1] (char. 82).

Ptr and MLtr: The presence of an intermandibularis is the basal feature of the tree being present in *Chimaera*, *Harriotta*, *Chlamydoselachus*, *Hexanchus*, *Heterodontus*, *Ginglymostoma*, *Squatina*, *Pristiophorus* and *Squalus*. In batomorphs, there is uncertainty on the basal-state reconstruction. However, the common state in the crown group is the absence of the intermandibularis muscle. A narrow band-shaped intermandibularis that originates on the hyomandibula and inserts on the posterior margin of Meckel's cartilage is a synapomorphy of clade 18 (Figures 1,2).

78. **<u>Antimeres of upper and lower jaws</u>:** (0) Separated; (1) fused. Taken from Aschliman et al. [1] (char. 40). *Myliobatis* shows a polymorphic (0 and 1) state as *M. freminvillei* exhibits the condition of having the antimeres unfused [30].

Ptr and MLtr: The separation of the antimeres of the palatoquadrate and Meckel's cartilage is the basal feature for the chondrichthyan tree, with the subsequent fusion of the antimeres in the (*Aetobatus*, (*Rhinoptera*, *Mobula*)) clade.

79. **<u>Meckel's cartilage</u>:** (0) Not expanded laterally; (1) expanded medially. Taken from Aschliman et al. [1] (char. 41).

Ptr and MLtr: A narrow Meckel's cartilage near the symphysis is the basal feature for the chondrichthyan tree, with the subsequent gain of the

expanded state as a shared feature between *Myliobatis*, *Aetobatus* and *Rhinoptera*.

80. **<u>Wing-like process in Meckel's cartilage</u>:** (0) Absent; (1) present. Taken from Aschliman et al. [1] (char. 42).

Ptr and MLtr: The absence of the wing-like process in the Meckel's cartilage is the basal feature for the chondrichthyan tree, with the subsequent gain of the process in clade 16 (Figures 1,2).

81. **<u>Infraorbital loop of suborbital and infraorbital canals</u>:** (0) Absent; (1) present. Taken from Aschliman et al. [1] (char. 21).

Ptr and MLtr: The lack of an infraorbital loop of suborbital and infraorbital canals is the basal state for the chondrichthyan tree. The presence of an infraorbital loop is a unique feature and could be a synapomorphy for the stingray's clade 14 (Figures 1,2) if the fossil taxa are deactivated.

82. **<u>Subpleural loop of the hyomandibular canal</u>:** (0) Broad rounded; (1) loop forms a lateral hook; (2) lateral aspects of loop are nearly parallel. Taken from Aschliman et al. [1] (char. 22).

Ptr and MLtre: The presence of a rounded loop of the hyomandibular canal is the basal state for the chondrichthyan tree. The subpleural loop forming a lateral hook is independently gained in *Hypanus* and *Gymnura,* and the subsequent gain of the lateral aspects of the subpleural loop's nearly parallel state as a shared feature of clade 16 (Figures 1,2).

83. **<u>Abdominal canal on coracoid bar</u>:** (0) Absent; (1) present. Modified from Aschliman et al. [1] (char. 24). The original character was split into two different ones (83–84) to increase the grouping information regarding the variation on the canals on the coracoid bar. The coding for *Pristis* was changed based on Wueringer et al.'s [30] (Text-Figure 1) observations.

Ptr and MLtr: The lack of an abdominal canal on the coracoid bar is the basal state for the chondrichthyan tree. Within batomorphs, the electric skates (clade 18; Figures 1,2) and stingrays (clade 16) retain the plesiomorphic state. The presence of a canal is widely distributed among batomorphs (Rajiformes = *Raja*; Torpediniformes = *Platyrhina, Platyrhinoidis*; Rhinopristiformes = *Rhynchobatus, Glaucostegus, Rhinobatos, Pseudobatos, Trygonorrhina, Zapteryx, Aptychotrema* and Myliobatiformes = *Zanobatus*), and according to the present topology, this is considered a single gain between these groups.

MLtr: The loss of the canals is recovered as an independent event and a synapomorphy of clades 18 and 16 (Figure 2).

84. **<u>Abdominal canal on coracoid bar (if present)</u>:** (0) Groove, cephalic lateral line forms abdominal canal on coracoid bar; (1) pores.

Ptr: The presence of pores in the canal is the basal state for clade 27 (Figure 1) being present in *Platyrhina, Platyrhinoidis* and *Zanobatus*. The presence of a groove is a shared feature for clade 19 (Figure 1). However, there is uncertainty regarding the basal state for Rhinopristiformes as *Trygonorrhina, Zapteryx* and *Aptychotrema* present pores.

MLtr: The presence of a groove is the basal state for the batomorphs. The presence of pores is a shared feature of clade 24 (Figure 2).

85. **Lateral tubes of subpleural loop:** (0) Unbranched; (1) branched. Taken from Aschliman et al. [1] (char. 23).

Ptr and MLtr: The basal state for the chondrichthyan tree is the presence of an unbranched lateral tube of the subpleural loop, with the independent gain of the branched state in *Urobatis* and *Urotrygon*.

86. **Scapular loops of scapular canals:** (0) Absent; (1) present. Taken from Aschliman et al. [1] (char. 25).

Ptr: The presence of scapular loops over the pectoral girdle is a shared feature of clade 14 (Figure 1) and could be a synapomorphy for the clade if the fossil taxa were deactivated.

MLtr: The more resolved Myliobatiformes clade recovering the presence of scapular loops over the pectoral girdle is a synapomorphy of clade 14 (Figure 2).

87. **Cephalic lateral line canal on ventral surface:** (0) Present; (1) absent. Taken from Aschliman et al. [1] (char. 20).

Ptr and MLtr: The presence of a cephalic lateral line is the basal feature for the chondrichthyan tree. The lack of cephalic lateral canals on the ventral side is a synapomorphy for clade 18 (Figures 1,2).

88. **Second synarcual:** (0) Absent; (1) present. Taken from Aschliman et al. [1] (char. 54).

Ptr and MLtr: The lack of a second synarcual is the basal state for the chondrichthyan tree. The presence of a thoracolumbar synarcual, generally separated from the cervical synarcual, is recovered as a synapomorphy of clade 14 (Figures 1,2).

89. **Caudal vertebrae:** (0) Diplospondylus; (1) fused. Taken from Aschliman et al. [1] (char. 80).

Ptr: Diplospondyly is the basal state among elasmobranchs and possibly the chondrichthyan tree (being present in holocephalians). The fusion of the caudal vertebrae forming a tube is a shared feature for clade 15 (Figure 1) with possible independent gains in *Hypanus*, *Potamotrygon*, *Neotrygon*, *Gymnura*, and †*Tethytrygon* (considering the polymorphic arrangement of this taxa).

MLtr: The fusion of the caudal vertebrae forming a tube is a synapomorphy of clade 32 (Figure 2).

90. **Ribs:** (0) Absent; (1) present. Taken from Marramà [32] (char. 92).

Ptr and MLtr: The presence of ribs is the basal state for the euselachians. The loss of ribs is recovered as an independent loss and a synapomorphy of the stingray clade 14 (Figures 1,2) and rajids clade.

91. **Radial calcification:** (0) Crustal; (1) catenated. Taken from Aschliman et al. [1] (char. 104).

Ptr: The crustal calcification is the basal feature for the chondrichthyan tree. The presence of catenated calcification is a synapomorphy of the rajids clade, with an independent gain in the stingrays, being present in †*Asterotrygon*, †*Heliobatis*, *Urolophus*, *Urobatis*,

Hypanus, *Neotrygon*, †*Lessiniabatis*, †*Arechia* and †*Tethytrygon*. The genera *Potamotrygon* and *Urotrygon* present variations in the type of radial calcification depending on the portions of the pectoral fins with basal radials: those closer to the propterygium, mesopterygium and metapterygium present crustal calcification and subsequent series present catenated calcification.

MLtr: Presents a similar reconstruction to the parsimony tree. The catenated calcification is recovered as an independent gain and a synapomorphy of clades 30 and 7 (Figure 2).

92. **Radial calcification (if catenated):** (0) Two chains; (1) four chains (New). This character includes the remaining variation observed by Schaefer and Summers [33] regarding the number of chains

Ptr and MLtr: The presence of two chains in the pectoral radials is a shared feature of clade 7 (Figures 1,2), while the presence of are four-chained pectoral radials is a shared feature of clade 14 (Figure 1) and 29 (Figure 2).

93. **Suprascapula:** (0) Absent; (1) fused medially; (2) unfused medially. Modified from Aschliman et al. [1] (char. 6).

Ptr: The lack of a suprascapula is the basal state for the chondrichthyan tree. The presence of the "suprascapula" cartilages [also see 34,35] is a synapomorphy of clade 2 (Figure 1) with an independent gain in *Squatina*. A medially developed suprascapula, as a single piece over the vertebral column, connecting the scapulocoracoid antimeres, is a synapomorphy for the batomorph crown group (clade 6; Figure 1).

MLtr: The basal placement of the Rajiformes (rajids+sclerorynchids) clade causes uncertainty on reconstructing the basal state for the batomorph crown group, as a suprascapula is missing in most sclerorynchoids. Consequently, this character is not recovered as a synapomorphy for the crown group, presenting independent gains in rajids clade and †*Libanopristis* and clade 23 (Figure 2).

94. **Suprascapula interaction with axial skeleton (if fused medially):** (0) Interacts with axial skeleton (articulated or fused); (1) free from axial skeleton (New).

Ptr and MLtr: The presence of an interaction (i.e., fused or articulated) between the suprascapula and the axial skeleton is the basal state for the crown group (fossil remains of the Jurassic batomorphs †*Kimmerobatis*, †*Asterodermus*, †*Spathobatis* and †*Belemnobatis* lack a suprascapula or, at least, a calcified one). The absence of interaction between the suprascapula and the axial skeleton is a synapomorphy of clade 18 (Figures 1,2).

95. **Suprascapula (if interacts with axial skeleton):** (0) Articulates with vertebral column; (1) fused medially to synarcual; (2) fused medially and laterally to synarcual (New).

Ptr: The presence of a suprascapula is fused medially to the synarcual is a synapomorphy of the Rajiformes. The medial and lateral fusion of the suprascapula and synarcual is a synapomorphy of Myliobatiformes.

MLtr: The presence of a suprascapula fused medially to the synarcual is not recovered as a synapomorphy of the Rajiformes, as there are uncertainties regarding the basal state, being inapplicable for the

Jurassic batoids as this cartilage is missing. The medial and lateral fusion of the suprascapula and synarcual is a synapomorphy of Myliobatiformes.

96. **Suprascapula-scapula articulation:** (0) Curved; (1) crenate; (2) ball and socket; (3) straight. Modified from Aschliman et al. [1] (char. 53).

Ptr: A crenated articulation between the scapula and suprascapula is the common feature of the batomorph crown group, being present in Torpediniformes = *Platyrhina*, †*Eoplatyrhina*, *Platyrhinoidis*, †*Tingitanius* and Rhinopristiformes = "†*Rhinobatos*" *maronita*, "†*R.*" *latus*, †*Stahlraja*, †*Tlalocbatus*, *Pristis*, *Rhynchobatus*, *Glaucostegus*, Rhina, *Rhinobatos*, *Pseudobatos*, †*Eorhinobatos*, †*Pseudorhinobatos*, *Trygonorrhina*, *Zapteryx*, *Aptychotrema* and †*Iansan*. A straight-shape articulation surface is a synapomorphy of clade 18 (Figure 1). A curved-shape articulation surface is a synapomorphy of the Rajiformes. A ball-and-socket articulation is a synapomorphy of the Myliobatiformes.

MLtr: There is uncertainty regarding the basal state for the batomorphs crown group as the placement of Rajiformes, at the base of this group creates conflict within the curved and crenate states. For the reaming batomorphs, both topologies recovered similar character reconstructions, with the crenated articulation as the basal state for the batomorphs crown group, and the straight and ball-and-socket articulations being synapomorphies for the electric skates and Myliobatiformes clade, respectively.

97. **Crenated suprascapula (variations):** (0) With lateral projections; (1) thin upper and lower lobes; (2) upper lobe wider than lower; (3) of similar size and wide (New).

Ptr and MLtr: A suprascapula with a narrow and larger upper lobe, the basal state for the Rhinopristiformes, being present in *Rhynchobatus*, *Glaucostegus*, *Rhinobatos*, *Pseudobatos*, *Aptychotrema* and †*Stahlraja*. Within the Rhinopristiformes, a suprascapula with an upper lobe wider than the lower lobe is a synapomorphy of clade 13 (Figures 1,2), while the presence of a suprascapula with both lobes of similar size is a synapomorphy of the (*Zapteryx*, *Trygonorrhina*) clade.

Ptr: The presence of lateral projections on the lower lobe of the suprascapula is a shared feature between *Platyrhina* and *Platyrhinoidis*. We could not determine the state in the fossil thornbacks (†*Tethybatis*, †*Tingitanius* and †*Eoplatyrhina*), causing uncertainty in the state reconstructions for their clade.

MLtr: The presence of a suprascapula with thin upper and lower lobes is the basal state of clade 23 (Figure 2). There is a subsequent independent gain of the lateral projections state for clade 27 (Figure 2) caused by the paraphyletic state of the extant thornbacks (*Platyrhina* and *Platyrhinoidis*).

98. **Scapular process-scapula:** (0) Fused; (1) articulated (New).

Ptr and MLtr: The fusion between the scapular process and the scapula is the basal state within the chondrichthyan tree. The genus *Squalus* presents variation the species *Squalus acanthias* and *S. megalops* present a fused process, while *S. mitsukurii* and S. *brevirostris* present an articulation between the process and the scapula. This variation is also present in Hexanchiformes, with *Hexanchus griseus* presenting the fused state and *Chlamydoselachus anguineus* presenting the articulated state. The

articulated state is a shared feature between *Heterodontus* and *Chlamydoselachus*.

99. **Scapular process:** (0) Short and dorsally directed; (1) long, U-curved and posteriorly directed; (2) short and posterodorsally directed. Modified from Aschliman et al. [1] (char. 86). An extra state was included to account for the variation observed in *Pseudobatos*.

Ptr and MLtr: The presence of a short, posterodorsally directed scapular process is an autapomorphy of *Pseudobatos*. The presence of a short and dorsally directed scapular process is the basal state for the chondrichthyan tree. The presence of a long, U-shaped and posterodorsally directed scapular process, is a synapomorphy of clade 18 (Figures 1,2).

100. **Scapular process:** (0) Without fossa; (1) with fossa. Taken from Aschliman et al. [1] (char. 57).

Ptr: The presence of a scapular process without fossa is the basal state for the chondrichthyan tree. The presence of a fossa or foramen is a shared feature of clade 14 (Figure 1).

MLtr: The presence of a scapular process without fossa is the basal state for the chondrichthyan tree. The presence of a fossa or foramen is a shared feature of clade 29 (Figure 2).

101. **Scapulocoracoid/pterygia articulation:** (0) Facets; (1) facets/condyles; (2) condyles (new). Based on observations by da Silva and de Carvalho [34].

Ptr: There is uncertainty regarding the basal-state reconstruction in the chondrichthyan tree as †*Doliodus*, †*Ozarcus*, †*Cobelodus*, *Chimaera*, and *Harriotta* states are unknown. The presence of an articulation between the scapulocoracoid and pectoral elements composed by facets is a synapomorphy of the (*Scyliorhinus*, *Mustelus*) clade, with independent gains in †*Hybodus* and †*Tribodus*. The combination of facets and condyles in the articulation between the pterygia and the scapulocoracoid is a synapomorphy of clade 4 (Figure 1) with an independent gain of the facet-state *Hexanchus*. Within Elasmobranchii, the basal state is the presence of condyles as the means of articulation of the pectoral elements, present in selachians (*Heterodontus*, *Squatina*, *Pristiophorus*, *Ginglymostoma*, *Hemiscyllium*) and batomorphs.

MLtr: The presence of facets for the articulations of the proximal pectoral elements is the basal state for Euselachian. The combination of facets and condyles for the articulation of the pterygia is a synapomorphy of clade 4 (Figure 2), with the subsequent independent gains of the only full condyle articulation as a synapomorphy in clades 2 and 5, and in the batomorphs.

102. **Condyles:** (0) Single condyle; (1) pro+mesocondyle; (2) meso+metacondyle; (3) three condyles (new). Based on observations by da Silva and de Carvalho [34].

Ptr: The presence of a single condyle is a shared feature between *Heterodontus* and *Hemiscyllium*. The presence of two condyles—one for the articulation of the of the pro+mesopterygium, and the metapterygium—is a shared feature between *Squatina* and *Ginglymostoma*. The presence of a single condyle for the articulation of the meso+metapterygium and a facet for propterygium is a synapomorphy

for clade 4 (Figure 1) (this is not recovered in the ML tree). The presence of three separated condyles is the basal feature in the shark+batoids clade, present in *Pristiophorus* and all batomorphs, and is also a synapomorphy and an independent gain of clade 4 (Figure 1).

MLtr: The presence of three separated condyles is recovered as the basal state for the batomorphs clade with an independent gain in *Pristiophorus*.

103. **Mesocondyle:** (0) Single and small; (1) segmented and small; (2) forming an elongated ridge. Modified from Aschliman et al. [1] (char. 59).

Ptr: A single, small and rounded mesocondyle is the basal state for batomorphs, being present in taxa of all major groups: Rajiformes = *Raja, Bathyraja,* †*Ostarriraja,* †*Cyclobatis,* †*Ptychotrygon,* †*Sclerorhynchus,* †*Libanopristis* and †*Asflapristis*; Torpediniformes = †*Titanonarke, Torpedo, Hypnos, Narcine, Narke, Temera, Platyrhina,* †*Eoplatyrhina, Platyrhinoidis,* †*Tingitanius*; Rhinopristiformes =“†*Rhinobatos*” *maronita,* “†*R.*” *latus, Pristis, Rhynchobatus, Glaucostegus, Rhina, Rhinobatos, Pseudobatos,* †*Britobatos,* †*Iansan* and †*Rhombopterygia*; Myliobatiformes = *Urolophus, Urobatis, Urotrygon, Hexatrygon, Plesiobatis, Hypanus, Potamotrygon*; and Jurassic batomorphs = †*Kimmerobatis,* †*Asterodermus,* †Spathobatis, †*Belemnobatis*. An elongated mesocondyle forming a ridge articulating the pectoral radials is recovered as a shared feature between *Trygonorrhina* and *Zapteryx Zanobatus, Plesiozanobatus, Hypanus, Neotrygon, Gymnura, Myliobatis, Aetobatus, Rhinoptera* and *Mobula*. A segmented mesocondyle split into two elements with the posterior one serving as articulation for a group of pectoral radials anteriorly to the metacondyle is a shared feature (possibly an independent gain) between †*Tethybatis,* †*Stahlraja,* †*Tlalocbatos* and *Aptychotrema*.

MLtr: Presents a similar basal-node reconstruction for this character as the parsimony tree. The separation of Trygonorrhinidae from the main Rhinopristiformes clade places the elongated mesocondyle, forming a sort of ridge for the articulation of the pectoral radials sate, as an independent gain and a synapomorphy for the (*Trygonorrhina, Zapteryx*) and the (*Zanobatus, Plesiozanobatus*) clades, with additional independent gains in clade 32 (Figure 2).

104. **Scapulocoracoid condyles:** (0) not horizontal, (1) horizontal. Taken from Aschliman et al. [1] (char. 59).

Ptr and MLtr: The lack of a horizontal arrangement of the pectoral condyles is a common feature between *Torpedo, Narcine, Narke* and *Temera*. There is an independent gain of the nonhorizontal sate in the selachians, being present in *Squatina, Pristiophorus* and *Ginglymostoma*. A horizontal arrangement of the scapulocoracoid condyles parallel to the anteroposterior axis of the body is the basal state for batomorphs.

105. **Ventral antimeres of scapulocoracoid:** (0) Fused/articulated; (1) separated. Taken from Villalobos-Segura et al. [4] (char. 85).

Ptr and MLtr: The fusion/articulation of the ventral antimeres of the pectoral girdle is the basal feature for Elasmobranchii. The separation of the ventral antimeres of the pectoral girdle scapula is the basal state for the chondrichthyan being present †*Hybodus,* †*Tribodus,* †*Doliodus,* †*Cobelodus* and †*Hamiltonichthys*. With an independent gain and a

synapomorphy for clade 18 (Figures 1,2) and the (*Chlamydoselachus*, *Hexanchus*) clade (not present in the MLtr).

106. **Elongated mesopterygium:** (0) Absent; (1) present. Based on Villalobos-Segura et al., [4] (char. 93).

Ptr: The lack of an elongated mesopterygium (Figure 7A–B) is the basal state for euselachians. The presence of a mesopterygium with its distal portion projected anteriorly following the external contour of the propterygium, giving them a relatively similar shape (Figure 7C–E), is a synapomorphy of the (†*Belemnobatis*, (†*Spathobatis*, (†*Kimmerobatis*, †*Asterodermus*))) clade with an independent gain in †*Hamiltonichthys*.

MLtr: The presence of a mesopterygium with its distal portion projected anteriorly following the external contour of the propterygium, giving them a relatively similar shape, is recovered as the basal state of the batomorphs clade with an independent gain in †*Hamiltonichthys* and the subsequent gain of the nonelongated state as a synapomorphy of the batomorphs crown group (clade 6, Figure 2).

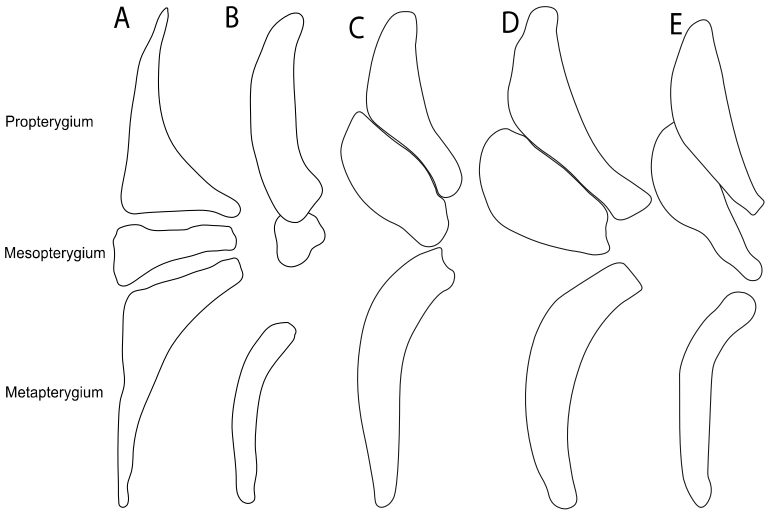

Figure S7 Interpretative drawings of the dorsal view of the scapulacoracoid and pectoral elements. State (0): A, †*Libanopristis hiram* (NHMUK PV P 75075); B, *Trygonorrhina fasciata* (MCZ 982S) from McEachran et al. [29] (Text-Figure 9A). State (1): C, †*Spathobatis bugesiacus* (NHUMK PV P 12067); D, †*Asterodermus platypterus* (JM-SOS-3647); E, †*Belemnobatis sismondae* (MNHN 15.753).

107. **Propterygium extending anteriorly:** (0) Absent; (1) present. Modified from Aschliman et al. [1] (char. 62).

Ptr and MLtr: The lack of an anteriorly elongated propterygium is the basal state for the chondrichthyan tree. The presence of an anterior elongated propterygium is a synapomorphy for the batomorphs (Figures 1,2).

108. **First segment of propterygium (if propterygium extends anteriorly)**: (0) Not reaching the nasal capsules; (1) reaches the level of nasal capsules; (2) extending well beyond the nasal capsules.

Ptr: A first segment of the propterygium not reaching the nasal capsules is the basal state for batomorphs, being present in several taxa across all batomorphs groups: Rajiformes = *Raja*, *Bathyraja*, †*Ostarriraja*, †*Ptychotrygon*, †*Sclerorhynchus*, and †*Libanopristis*; Torpediniformes =

†*Titanonarke, Torpedo, Hypnos, Narcine, Narke, Temera, Platyrhina,* †*Eoplatyrhina* and *Platyrhinoidis*; *Rhinopristiformes*; "†*Rhinobatos*" *maronita,* "†R." *latus,* †*Stahlraja,* †*Tlalocbatus, Pristis, Rhynchobatus, Glaucostegus, Rhina, Rhinobatos, Pseudobatos,* †*Eorhinobatos,* †*Pseudorhinobatos, Trygonorrhina, Zapteryx, Aptychotrema, Britobatos,* †*Iansan* and †*Rhombopterygia*; Myliobatiformes = *Zanobatus,* †*Plesiozanobatus* and Jurassic batomorphs = †*Kimmerobatis,* †*Asterodermus,* †*Spathobatis* and †*Belemnobatis*. A first segment of the propterygium reaching the nasal capsules is a synapomorphy for clade 14 (Figure 1). With the subsequent gain of the state "first segment of the propterygium extending beyond the level of the nasal capsule" as the basal state in clade 15 (Figure 1), with an independent gain in †*Cyclobatis*. This feature is also present in †*Asterotrygon,* †*Heliobatis, Hypanus* and *Gymnura*, but their polytomic arrangement make the reconstruction difficult.

MLtr: Presents a similar reconstruction to the parsimony tree. However, this topology recovers the first segment of the propterygium reaching the level of nasal capsules as a synapomorphy of clade 14 (Figure 2), with the subsequent gain of the first segment of the propterygium extending beyond the level of nasal capsules as a synapomorphy of clade 33 (Figure 2).

109.**Interaction between mesopterygium and propterygium:** (0) fused, (1) separated. (New). In batomorphs *Zanobatus,* †*Plesiozanobatus, Aetobatus* and *Rhinoptera* and also *Harriotta* and *Chimaera* this interaction is very different (-).

Ptr: A separated mesopterygium and propterygium is the basal state for euselachians. The fusion between mesopterygium and propterygium is a synapomorphy of the (*Hemiscyllium, Ginglymostoma*).

110. **Position of the articulation of the antorbital cartilage on nasal capsule:** (0) Lateral; (1) anterolateral; (2) posterolateral. Modified from de Carvalho [36] (char. 2).

Ptr and MLtr: The lateral position of the articulation between the antorbital cartilage and the nasal capsule is the basal state of batomorphs, being present among several taxa and groups: Rajiformes = *Raja, Bathyraja,* †*Ostarriraja,* †*Cyclobatis,* †*Ptychotrygon,* †*Sclerorhynchus,* †*Libanopristis,* †*Asflapristis* and †*Onchopristis*; Torpediniformes: *Platyrhina,* †*Eoplatyrhina, Platyrhinoidis* and †*Tingitanius*; Rhinopristiformes = †*Stahlraja,* †*Tlalocbatus, Trygonorrhina, Zapteryx, Aptychotrema* and †*Britobatos*; Myliobatiformes = *Zanobatus,* †*Plesiozanobatus,* †*Asterotrygon,* †*Heliobatis, Urolophus, Urobatis, Urotrygon, Plesiobatis, Hexatrygon, Hypanus, Potamotrygon, Neotrygon, Gymnura, Myliobatis, Aetobatus, Rhinoptera, Mobula,* †*Arechia,* †*Lessiniabatis,* †*Tethytrygon,* †*Arechia,* †*Lessiniabatis,* †*Promyliobatis* and †*Tethytrygon*; Jurassic batomorphs = †*Kimmerobatis,* †*Asterodermus,* †*Spathobatis,* †*Belemnobatis*. An anterolateral position of the articulation between the antorbital cartilage and the nasal capsules is a synapomorphy of clade 18 (Figures 1,2). The posterolateral placement of the articulation between the antorbital cartilage and the nasal capsules is a synapomorphy of clade 10 (Figures 1,2).

111.**Cross-bracing of pectoral radials**: (0) Absent; (1) present. Modified from Aschliman et al. [1] (char. 67). Based on Schaefer and Summers' [33] observations.

Ptr: The presence of inter-radial connections (cross-braces) between radials of the pectoral fin is a shared feature of clade 15 (Figure 1) with independent gains in †*Britobatos, Urotrygon, Gymnura*. The absence of cross-branching is a placed basal state for the chondrichthyan tree.

MLtr: The presence of inter-radial connections (cross-braces) between radials of the pectoral fin is a synapomorphy of clade 15 (Figure 2) with independent gains in †*Britobatos, Urotrygon, Gymnura*.

112. **Proximal section of propterygium:** (0) Does not surpass the procondyle; (1) extends posterior to procondyle. Taken from Aschliman et al. [1] (char. 64).

Ptr: The lack of a proximal projection of the propterygium of the pectoral girdle extending posteriorly to the procondyle and articulating between the procondyle and mesocondyle is the basal state of the elasmobranch clade. The presence of these projections is a synapomorphy of the Myliobatiformes, with an independent gain in *Platyrhina*.

MLtr: The presence of these projections is a synapomorphy of clade 28 (Fig 2).

113. **Mesopterygium:** (0) Present; (1) absent. Taken from Claeson et al. [5] (char. 34).

Ptr and MLtr: The lack of a mesopterygium is the basal state for the chondrichthyan tree, being absent in †*Doliodus*, †*Cobelodus*, *Harriotta* and *Chimaera*. The presence of a mesopterygium is a synapomorphy of euselachians. The subsequent loss of the mesopterygium is a synapomorphy of the (*Zanobatus*, †*Plesiozanobatus*) clade, with additional independent losses in *Aetobatus* and *Rhinoptera*.

114. **Pectoral fin radials:** (0) All articulate to pterygia; (1) some articulate directly with scapulocoracoid or to the ridge. Based on Villalobos-Segura et al. [4] (char. 65).

Ptr: The basal state for euselachian is the articulation of radials only to the basal elements of the pectoral fin. A direct articulation or a ridge for the attachment of the pectoral radials is a synapomorphy and an independent gain of clades 7, 10′ and 16 (Figs 1,2) and (*Zanobatus*, †*Plesiozanobatus*). "†*Rhinobatos*" *maronita*, "†*R.*" *latus*, "†*R.*" *hakelensis*, *Pristis*, *Rhynchobatus*, *Glaucostegus*, *Rhina*, *Rhinobatos*, *Pseudobatos* and *Gymnura* also present this feature.

MLtr: In addition to being a synapomorphy previously mentioned clades in the parsimony tree. The presence of a direct articulation or a ridge for the attachment of the pectoral radials as a synapomorphy of clade 35 (Figure 2).

115. **Paired fin rays:** (0) Aplesodic; (1) plesodic. Modified from Aschliman et al. [1] (char. 68). Based on da Silva and de Carvalho [33] (Text-Figure 19F), the coding of *Pristis* has been changed from aplesodic to plesodic (1).

Ptr and MLtr: The plesodic state is a synapomorphy for batomorphs. The aplesodic state is a shared state for holocephalians, hybodonts and selachians.

116. **Pectoral pterygial elements expanded distally and paddle-like**: (0) Absent; (1) present. Based on Villalobos-Segura et al. [4] (char. 39).

Ptr and MLtr: The presence of paddle-like elements is a synapomorphy of sclerorhynchoids (clade 8; Figures 1,2).

117. **Lateral prepelvic processes:** (0) Absent; (1) present. The modification of this character from the multistate coding used in McEachran and Dunn [37] (char. 36) is because the three states proposed by the authors (i.e., short to moderately long; extremely long with acute tips; and extremely long with biramous tips) are difficult to interpret in the fossil specimens. Because of this, binary coding (presence/absence) is used.

Ptr and MLtr: The absence of lateral prepelvic processes is the basal state for the chondrichthyan tree. The presence of this lateral process is and independent gain and a synapomorphy of clades 7 and 18 (Figures 1,2).

118. **Postpelvic processes:** (0) Absent; (1) present. Modified from Claeson et al. [5] (char. 37). Initially observed in *Platyrhina* and *Platyrhinoidis* only by de Carvalho [36], the coding of this character was changed for *Pseudobatos* and *Torpedo* based on da Silva et al.'s [38] observations, as these two taxa also show postpelvic processes.

Ptr and MLtr: The lack of postpelvic processes is the basal state of the chondrichthyan tree.

Ptr: The presence of these processes is a synapomorphy of the Torpediniformes and the Jurassic batomorphs clade, with additional independent gains in *Hemiscyllium* and Rhinopristiformes, being present in †*Tlalocbatus*, *Pseudobatos*, *Rhinobatos*, *Glaucostegus*, *Zapteryx* and *Aptychotrema*.

MLtr: The presence of these processes is a synapomorphy of the batomorphs clade, with a subsequent loss in the crown group. Additional independent gains of the postpelvic processes are recovered as synapomorphies of clades 22 and 24 (Figure 2), with a subsequent loss and a synapomorphy in the (*Temera*, *Narke*) clade and in the Myliobatiformes.

119. **Posterior margin of puboischiadic bar:** (0) Straight or bending anteriorly; (1) convex (New).

Ptr and MLtr: The presence of an anterior margin roughly straight or bending anteriorly is the basal state for the chondrichthyan tree. The presence of a puboischiadic bar bending towards the tail is a synapomorphy of the (*Squatina*, †*Pseudorhina*) clade and clade 18 (Figures 1,2).

120. **Anterior margin of puboischiadic bar (if posterior margin straight or concave):** (0) Straight; (1) anteriorly arched (New). This character is proposed to group the different patterns of those taxa that present a posterior margin of the puboischiadic bar straight or bending anteriorly.

Ptr and MLtr: The presence of a straight puboischiadic bar is the basal state for the chondrichthyan tree. Arching of the anterior margin of the puboischiadic bar is a synapomorphy of clade 12 (Figures 1,2), with independent gains as a synapomorphy of the Myliobatiformes and autapomorphy of *Heterodontus* and *Mustelus*.

121. **Medial process of puboischiadic bar:** (0) Absent; (1) present. The original multistate coding proposed by Aschliman et al. [1] (char. 70) was changed to a binary one to increase the grouping information on the presence of the process.

Ptr: The absence of a middle process in the puboischiadic bar is the basal feature of the chondrichthyan tree. The presence of a middle process in the pelvic girdle is a synapomorphy of clade 13 (Figure 1) with an independent gain in clade 14 (Figure 1) and possibly a synapomorphy if the fossil species are deactivated.

MLtr: The presence of a middle process is an independent gain and a synapomorphy of clades 13, 21 and 36 (Figure 2).

122. **Overdevelopment of first pelvic radial:** (0) Absent; (1) present (New).

Ptr and MLtr: The lack of an overdeveloped first pelvic radial is the basal feature of the chondrichthyan tree. The presence of overdevelopment in the first pelvic radial, which in the extant taxa results in a pelvic fin with two lobes, is a synapomorphy of clade 7 (Figures 1,2).

123. **First pelvic radial (if not overdeveloped):** (0) Band-like; (1) slightly expanded distally, articulating with several segments in a parallel fashion; (2) rod-like and articulates with a single radial segment. Taken from Aschliman et al. [1] (char. 71).

Ptr: A first pelvic radial with a band-like shape is the basal state for the chondrichthyan tree (Figure 8A). A distal expanded first radial that articulates with at least two radials in parallel fashion is a synapomorphy of the (†*Kimmerobatis*, †*Asterodermus*) clade and with an independent gain and a synapomorphy of the crown batomorphs group (clade 6; Figure 1), being present in *Torpedo*, *Hypnos*, *Platyrhina*, *Platyrhinoidis*, "†*Rhinobatos*" *maronita*, "†*R.*" *latus*, †*Stahlraja*, †*Tlalocbatus*, *Pristis*, *Rhynchobatus*, *Glaucostegus*, *Rhina*, Rhinobatos, *Pseudobatos*, *Trygonorrhina*, *Zapteryx*, *Aptychotrema*, *Urobatis*, *Urotrygon*, *Hexatrygon*, *Hypanus*, *Potamotrygon*, *Gymnura*, *Myliobatis*, *Aetobatus*, *Rhinoptera*, *Mobula*, †*Sclerorhynchus*, †*Libanopristis* and †*Promyliobatis* (Figure 8B). A narrow, long first pelvic radial that articulates with a single radial segment is a synapomorphy of clade 19 (Figure 1), with an independent gain in Myliobatiformes, being a synapomorphy of the (*Zanobatus*, †*Plesiozanobatus*) clade, but also is present in †*Asterotrygon*, †*Heliobatis*, *Urolophus* and *Neotrygon* (Figure 8C).

MLtr: A distal expanded first radial that articulates with at least two radials parallelly is a synapomorphy of clade 37 (Figure 2). A narrow, long first pelvic radial that articulates with a single radial segment is an independent gain in the (*Zanobatus*, †*Plesiozanobatus*) and clade 34.

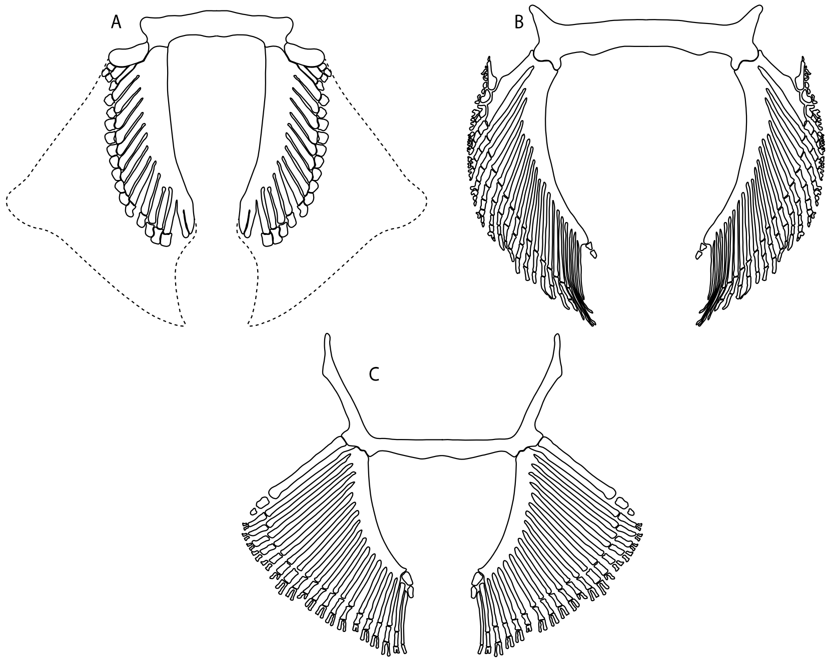

Figure S8 Dorsal view of pelvic gridle. State (0): A, *Squalus brevirostris* (AMNH 258171, https://sharksrays.org/), State (1): B, *Zapteryx brevirostris* (uncatalogued material). State (2): C, *Torpedo torpedo* (AMNH 4128) from da Silva et al. [38] (Text-Figure 8B).

124. **<u>Pelvic girdle halves</u>:** (0) Separated; (1) fused. Modified from Maisey [2] (char, 37), based on Klug et al. [12] (Text-Figure 2), Stumpf et al. [39] and Coates et al.'s [40] observations. Current fossil evidence suggests that the separation of two halves or at least a not very well-mineralized mid-bar of the pelvic girdle is the basal state across hybodont-like sharks (SMNS 10062) (NHMUK PV P 339).

Ptr and MLtr: The presence of two pelvic halves is a shared feature between holocephalians and hybodonts. A fused pelvis is a shared feature among elasmobranchs, and due to the uncertainty at the base of the tree recovered, it is not a synapomorphy for Elasmobranchii in the current trees.

125. **<u>Reduced number of cartilages between basipterygium and clasper</u>:** (0) Absent; (1) present. Taken from Maisey [2] (char. 48).

Ptr and MLtr: The reduced number of segments between the basipterygium and mixipterygial cartilage (Figure 9B–C) is a shared feature among Elasmobranchii. Due to the uncertainty associated with base of the tree retrieved, it is not a synapomorphy for Elasmobranchii.

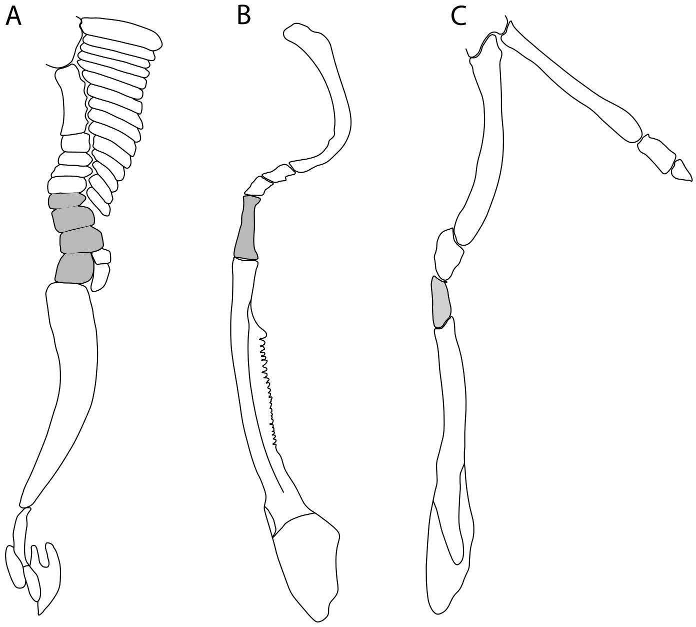

Figure S9 Dorsal view of pelvic gridle. State (0): A, †*Hybodus hauffianus* (SMNS 10062). State (1): B, *Zanobatus schoenleinii* (MNHN N/C) from da Silva et al. [38] (Text-Figure 4A); C, *Zapteryx brevirostris* (uncatalogued).

126. **Clasper length:** (0) Short; (1) long. Taken from Aschliman et al. [1] (char. 73).

Ptr: The presence of long claspers is the basal feature of the chondrichthyan tree, with an independent gain in thornbacks (clade 23; Figure 1). The presence of short claspers is a synapomorphy for the selachians, with additional gains *Harriotta* and as a synapomorphy of clade 26 (Figure 1).

MLtr: The presence of short claspers is recovered as a synapomorphy for selachians with an additional gain in clade 25 (Figure 2) and the Myliobatiformes.

127. **Dorsal margin of clasper cartilages:** (0) Lacks medial flange; (1) possesses medial flange. Taken from Aschliman et al. [1] (char. 75).

Ptr and MLtr: A medial flange in the dorsal margin of the clasper cartilage extending for most of the clasper length is a synapomorphy of the Myliobatiformes, with an independent gain in *Narcine* and *Narke*.

128. **Ventral terminal cartilages:** (0) Simple; (1) ventral terminal cartilages are free distally and form sentinel components or are fused with ventral marginal cartilages; (2) ventral terminal cartilages folded ventrally along its long axis to form a convex flange. Taken from Aschliman et al. [1] (char. 78).

Ptr and MLtr: A simple ventral terminal cartilage in the claspers is the basal feature of the chondrichthyan tree, with the subsequent gain of the ventral terminal cartilages being distally free, forming sentinel components or fused with ventral marginal cartilages state in *Raja* and *Bathyraja*. The presence of ventral terminal cartilages folded ventrally along the long axis to form a convex flange is a synapomorphy for the Myliobatiformes.

129.**Ventral terminal cartilages:** (0) Attached over length to axial cartilage; (1) free of axial cartilage. Taken from Aschliman et al. [1] (char. 79).

Ptr: A ventral terminal cartilage free of the axial cartilage is a shared feature of clade 7 (Figures 1,2) if *Ostarriraja* is deactivated. This state is also present in *Hypanus*, †*Tethytrygon* and *Neotrygon*, which could represent an independent gain considering the state placement in different clades.

130.**Two dorsal fin spines:** (0) Absent; (1) present. Taken from Maisey [2] (char. 17).

Ptr and MLtr: The presence of two dorsal-fin spines is a synapomorphy of the Hybodontiformes and the (*Squalus*, †*Protospinax*) clades, with additional independent gains in *Heterodontus* and †*Belemnobatis*.

131.**Enameloid layer on fin spines (if two dorsal spines):** (0) Absent; (1) present. Taken from Maisey [2].

Ptr: The presence of a shiny enameloid layer is the basal state of the selachian clade being present in *Heterodontus*, *Squalus*, †*Protospinax* and †*Belemnobatis*.

132. **Serrated tail sting:** (0) Absent; (1) present. Taken from Aschliman et al. [1] (char. 14).

Ptr: The presence of a serrated tail sting is a shared feature between †*Asterotrygon*, †*Heliobatis*, *Hexatrygon*, *Urolophus*, *Urobatis*, *Urotrygon*, *Plesiobatis*, *Hypanus*, *Potamotrygon*, *Neotrygon*, *Gymnura*, †*Arechia*, †*Promyliobatis*, †*Tethytrygon*, *Myliobatis*, *Aetobatus*, *Rhinoptera* and *Mobula*. Its absence in †*Lessiniabatis* is a secondary loss.

MLtr: The presence of a serrated tail sting is a synapomorphy of clade 14 (Figure 2).

133. **Placoid scales:** (0) Scarce or absent; (1) present. The original multistate coding proposed by Aschliman et al. [1] (char. 15), was difficult to determinate on fossil species. Considering this and to maximize the grouping information, a binary coding was used.

Ptr: The presence of placoid scales is the basal feature in the chondrichthyan tree. The loss of placoid scales is a synapomorphy of holocephalians, with independent gains in clades 18 and 14 (Figure 1).

MLtr: The loss of placoid scales is a synapomorphy of holocephalians, with independent gains in clades 18 and 30 (Figure 2).

134.**Malar and alar thorns:** (0) Absent; (1) present. Taken from Aschliman et al. [1] (char. 17).

Ptr and MLtr: The presence of alar and malar thorns in mature males is a shared feature between *Raja* and *Bathyraja*. It is not a synapomorphy as the state for fossil skates and †*Cyclobatis*, and †*Ostarriraja* remains undetermined.

135.**Enlarged placoid scales (thorns):** (0) Absent; (1) present. Taken from Aschliman et al. [1] (char. 16).

Ptr: The presence of enlarged placoid scales is a synapomorphy of the (†*Onchopristis*, †*Ischyrhiza*) clade, with independent gains as a

synapomorphy of clades 7, 10′ and 30 (Figure 1); this feature is also present in †*Hamiltonichthys*, *Platyrhina*, *Platyrhinoidis*, †*Belemnobatis*, *Glaucostegus*, *Rhina*, *Rhinobatos*, *Pseudobatos* and *Zanobatus*.

MLtr: The absence of enlarged placoid scales is the basal state for the chondrichthyan tree. The presence of enlarged placoid scales is a synapomorphy of clade (†*Onchopristis*, †*Ischyrhiza*), with independent gains as a synapomorphy of clades 7, 10′ and 30 (Figure 2).

136.**Lateral rostral dermal denticles:** (0) Absent; (1) present. Taken from Villalobos-Segura et al. [4] (char. 90).

Ptr: The presence of lateral series of enlarged rostral denticles is a synapomorphy of clade 9 (Figures 1,2) with independent gains in *Pristiophorus* and *Pristis*.

137.**Central-ventral rostral dermal denticle series:** (0) Present; (1) absent. Modified from Villalobos-Segura et al. [41] (char. 8), based on Welten et al.'s [42] observations.

Ptr and MLtr: The presence of a central-ventral series of enlarged dermal denticles in the rostrum is a synapomorphy of the (†*Sclerorhynchus*, †*Libanopristis*) clade.

138. **Second transverse keel on teeth:** (0) Absent; (1) present. Taken from Herman et al. [43] and Marramà et al. [44] (char. 98).

Ptr: A second transverse keel in the crown of teeth is a synapomorphy of the (†*Ptychotrygon*, †*Asflapristis*) with an independent gain in †*Libanopristis*; this character is also present in *Urobatis*, *Urolophus* and †*Arechia*.

MLtr: Presents a similar reconstruction for this feature as the parsimony tree. However, with *Urobatis* and *Urolophus*, †*Arechia* in a clade, this feature is a synapomorphy for clade 34 (Figure 2).

139. **File of enlarged "caniniform" teeth in the upper jaw:** (0) Absent; (1) present. Taken from Marramà et al. [44] (char. 102).

Ptr and MLtr: The presence of a file of enlarged caniniform teeth on the upper jaw is a synapomorphy of the (*Neotrygon*, †*Tethytrygon*) clade.

140. **Gill skeleton position:** (0) Partly beneath otico-occipital regions; (1) posterior to occipital region. Taken from Coates et al. [45] (char. 66).

Ptr and MLtr: A gill skeleton partly beneath the otico-occipital region is a synapomorphy of the holocephalian clade.

141. **Tribasal pectoral fin:** (0) Absent; (1) present. †*Doliodus* and †*Cobelodus* codification changed from Frey's et al. [46] was changed as there is no evidence to support the presence of tribasal pectoral fin for †*Doliodus* (unibasal) [48,49]. The pectoral remains of †*Cobelodus* (FMNH PF 8009; Zangerl and Case [50] (Text-Figure 21)) also suggest a different configuration, possibly dibasal.

Ptr and MLtr: The presence of a tribasal pectoral fin is a synapomorphy of the euselachian clade (however, this is very variable in other fossil groups).

142.**Precerebral fontanelle:** (0) Absent; (1) present. Taken from Coates et al. [13] (char. 62).

Ptr and MLtr: The absence of a precerebral fontanelle is a synapomorphy of the (*Chimaera*, *Harriotta*) clade.

References

1. Aschliman, N.C., Claeson, K.M., McEachran, J.D. Phylogeny of Batoidea. In Biology of Sharks and Their Relatives. (In: *Biology of Sharks and Their Relatives,* Carrier, J.C., Musick, J.A., Heithaus, M.R., Eds.; CRC Press: FL, USA, 2012, Volume 2, pp 57–95.
2. Maisey, J.G. Chondrichthyan phylogeny: a look at the evidence. *J. Vertebr. Paleontol*. **1984**, 4, 359–371.
3. Maisey, J.G. Higher elasmobranch phylogeny and biostratigraphy. *Zool. J. Linn. Soc*. **1984**, 82, 33–54.
4. Villalobos–Segura, E., Underwood, C.J., Ward, D.J., Claeson, K.M. The first three–dimensional fossils of Cretaceous sclerorhynchid sawfish: *Asflapristis cristadentis* gen. et sp. nov., and implications for the phylogenetic relations of the Sclerorhynchoidei (Chondrichthyes). *J. Syst. Palaeontol.*, **2019**, 17: 1847–1870. 10.1080/14772019.2019.1578832.
5. Claeson, K.M., Underwood, C.J., Ward, D. †*Tingitanius tenuimandibulus*, a new platyrhinid batoid from the Turonian (Cretaceous) of Morocco and the Cretaceous radiation of the Platyrhinidae. *J. Vertebr. Paleontol*. **2013**, 33, 1019–1036. 10.1080/02724634.2013.767266.
6. Maisey, J.G. The anatomy and interrelationships of Mesozoic hybodont sharks. *Am. Mus. Novit*. **1982**, 1–17.
7. Maisey, J.G. An evaluation of jaw suspension in sharks. *Am. Mus. Novit.***1980**, 1–48.
8. Wilga, C.A., Ferry, L.A. Functional anatomy and biomechanics of feeding in elasmobranchs. In *Fish physiology*. Shadwick, R.E., Farrell, A.P., Brauner, C.J., Eds.; Academic Press. London, UK, 2015, Vol. 34B, pp 153–187. 10.1016/B978-0-12-801289-5.00004-3
9. Maisey, J.G. The postorbital palatoquadrate articulation in elasmobranchs. *J. Morphol*. **2008**, 269, 1022–1040. 10.1002/jmor.10642. Maisey, J.G. The postorbital palatoquadrate articulation in elasmobranchs. *J. Morphol*. **2008**, 269, 1022–1040. 10.1002/jmor.10642.
10. Shirai, S. 1992. Squalean phylogeny: a new framework of" squaloid" sharks and related taxa. Hokkaido University Press: Japan, 1992. 212pp.
11. Lane, J.A., Maisey, J.G. 2012 The visceral skeleton and jaw suspension in the durophagous hybodontid shark *Tribodus limae* from the Lower Cretaceous of Brazil. *J. Paleontol*. **2012**, 86, 886–905. doi:10.1666/11–139.1. 10.1666/11-139.1.
12. Klug S. Monophyly, phylogeny and systematic position of the †Synechodontiformes (Chondrichthyes, Neoselachii). *Zool. Scr.* **2010**, 39, 37–49. 10.1111/j.1463-6409.2009.00399.x.
13. Pradel, A., Tafforeau, P., Maisey, J.G., Janvier, P. A new Paleozoic Symmoriiformes (Chondrichthyes) from the Late Carboniferous of Kansas (USA) and cladistic analysis of early chondrichthyans. *PLOS ONE* **2011**, 6, e24938. 10.1371/journal.pone.0024938.
14. Pradel, A., Maisey, J.G., Tafforeau, P., Mapes, R.H., Mallatt, J. A Palaeozoic shark with osteichthyan-like branchial arches. *Nature* **2014**, 509, 608–611. 10.1038/nature13195.
15. de Carvalho, M.R. Higher-level elasmobranch phylogeny, basal squaleans, and paraphyly. In *Interrelationships of fishes,* Stiassny, M.L., Parenti, L.R., Johnson, G.D., Eds.; Atlantic Press: NY, USA, 1996, pp 593–660.7
16. Maisey, J.G., Ehret, D.J., Denton, J.S. A new genus of Late Cretaceous angel shark (Elasmobranchii; Squatinidae), with comments on squatinid phylogeny. *Am. Mus. Novit*. **2020**, 1–29. 10.1206/3954.1.
17. Heemstra, P.C., Smith, M.M. Hexatrygonidae, a new family of stingrays (Myliobatiformes: Batoidea) from South Africa, with comments on the classification of batoid fishes. *Ichthyol. Bull*. **1980**, 43, 1–17.
18. de Carvalho, M., Bockmann, F.A., de Carvalho, M.R. Homology of the fifth epibranchial and accessory elements of the ceratobranchials among Gnathostomes: insights from the development of ostariophysans. *PLOS ONE* **2013** 8, e62389. 10.1371/journal.pone.0062389.
19. Shirai S. Phylogenetic relationships of the angel sharks, with comments on elasmobranch phylogeny (Chondrichthyes, Squatinidae). *Copeia* **1992**, 505–518.
20. de Carvalho M.R., Maisey J.G. The Phylogenetic Relationship of the Late Jurassic Shark *Protospinax* Woodward 1919 (Chondrichthyes: Elasmobranchii). In *Mesozoic Fishes Systematics and Paleoecology*, Arriatia, G., Viohl, G., Eds.; Verlag Dr. Friedrich Pfeil: München, Germany, 1996, pp. 9–46.
21. Miyake, T., McEachran, J.D. The morphology and evolution of the ventral gill arch skeleton in batoid fishes (Chondrichthyes: Batoidea). *Zool. J. Linn. Soc.* **1991**, 102, 75–100. 10.1111/j.1096-3642.1991.tb01537.x
22. Nishida, K. Phylogeny of the suborder Myliobatidoidei. *Memoirs of the faculty of fisheries Hokkaido University* **1990**, 37, 1–108.
23. Lovejoy, N.R. Systematics of myliobatoid elasmobranchs: with emphasis on the phylogeny and historical biogeography of neotropical freshwater stingrays (Potamotrygonidae: Rajiformes). *Zool. J. Linn. Soc.* **1996**, 117, 207–257. 10.1111/j.1096-3642.1996.tb02189.x.

24. Johanson, Z., Trinajstic, K., Carr, R., Ritchie, A. Evolution and development of the synarcual in early vertebrates. *Zoomorphology*. **2013**, 132, 95–110. 10.1007/s00435-012-0169-9.
25. Brito, P., Leal, M., Gallo, V. A new lower Cretaceous guitarfish (Chondrichthyes, Batoidea) from the Santana formation, Northeastern Brazil. *Boletim do Museu Nacional, Geologia* **2013**, 75, 1–13.
26. Marramà, G., Klug, S., De Vos, J., Kriwet, J. Anatomy, relationships and palaeobiogeographic implications of the first Neogene holomorphic stingray (Myliobatiformes: Dasyatidae) from the early Miocene of Sulawesi, Indonesia, SE Asia. *Zool. J. Linn. Soc.* **2018**, 184, 1142–1168. 10.1093/zoolinnean/zly020.
27. Reif, W.E**.** Tooth enameloid as a taxonomic criterion: 1. A new eusalachian shark from the Rhaetic-Liassic boundary. *Neues Jahrb. fur Geol. Palaontol. Monatshefte*, **1977,** 565–576.
28. Riley, C., Cloutier, R., Grogan, E.D. Similarity of morphological composition and developmental patterning in paired fins of the elephant shark. *Sci. Rep.* **2017**, 7, 1–10. 10.1038/s41598-017-10538-0.
29. McEachran, J.D., Dunn, K.A., Miyake, T. Interrelationships of the batoid fishes (Chondrichthyes: Batoidea). In *Interrelationships of fishes,* Stiassny, M.L., Parenti, L.R., Johnson, G.D., Eds.; Atlantic Press: NY, USA, 1996, pp 63–84.
30. Aschliman, N.C. Interrelationships of the durophagous stingrays (Batoidea: Myliobatidae). E*nviron. Biol. Fishes*. **2014**, 97, 967–979. 10.1007/s10641-014-0261-8.
31. Wueringer, B., Squire, L., Collin, S. The biology of extinct and extant sawfish (Batoidea: Sclerorhynchidae and Pristidae). *Rev. Fish Biol*. **2009**, 19,445–465, 10.1007/s11160-009-9112-7. 10.1007/s11160-009-9112-7.
32. Marramà, G., Carnevale, G., Claeson, K.M., Naylor, G.J., Kriwet, J. Revision of the Eocene "*Platyrhina*" species from the Bolca Lagerstätte (Italy) reveals the first panray (Batomorphii: Zanobatidae) in the fossil record. *J. Syst. Palaeontol*. **2020a**.18, 1519–1542. 10.1080/14772019.2020.1783380
33. Schaefer, J.T., Summers, A.P. Batoid wing skeletal structure: novel morphologies, mechanical implications, and phylogenetic patterns. *J. Morphol*. **2005,** 264, 34298–313. 10.1002/jmor.10331
34. da Silva, J.P.C., Carvalho, M.R. Morphology and phylogenetic significance of the pectoral articular region in elasmobranchs (Chondrichthyes). *Zool. J. Linn. Soc*. **2015**, 175, 525–568. 10.1111/zoj.12287.
35. da Silva, J.P.C., Vaz, D.F., Carvalho., M.R. Phylogenetic inferences on the systematics of squaliform sharks based on elasmobranch scapular morphology (Chondrichthyes: Elasmobranchii). *Zool. J. Linn. Soc*. **2018**, 182, 614–630. 10.1093/zoolinnean/zlx051.
36. de Carvalho, M.R. A Late Cretaceous thornback ray from southern Italy, with a phylogenetic reappraisal of the Platyrhinidae (Chondrichthyes: Batoidea). In *Mesozoic fishes Systematics Paleoenvironments and Biodiversity*, Arriatia, G., Tintori, A., Eds.; Verlag Dr. Friedrich Pfeil: München, Germany, 2004; Volume 3, pp. 75–100.
37. McEachran, J.D., Dunn, K.A. Phylogenetic analysis of skates, a morphologically conservative clade of elasmobranchs (Chondrichthyes: Rajidae). *Copeia* **1998**, pp 271–290. 10.2307/1447424
38. da Silva, J.P.C., Silva–Loboda, T., Souza, R.R. A new synapomorphy in the pelvic girdle reinforces a close relationship of *Zanobatus* and Myliobatiformes (Chondrichthyes: Batoidea). *J. Anat*. **2021**, 238: 874–885. 10.1111/joa.13354
39. Stumpf, S., Etches, S., Underwood, C.J., Kriwet, J. *Durnonovariaodus maiseyi* gen. et sp. nov., a new hybodontiform shark-like chondrichthyan from the Upper Jurassic Kimmeridge Clay Formation of England. *PeerJ* **2021** 9, e11362. 10.7717/peerj.11362.
40. Coates, M.I., Gess, R.W. A new reconstruction of *Onychoselache traquairi*, comments on early chondrichthyan pectoral girdles and hybodontiform phylogeny. *Palaeontology* **2007,** 50, 1421–1446. 10.1111/j.1475-4983.2007.00719.
41. Villalobos–Segura E, Kriwet J, Vullo R, Stumpf S, Ward DJ, Underwood CJ. The skeletal remains of the euryhaline sclerorhynchoid† *Onchopristis* (Elasmobranchii) from the "Mid"–Cretaceous and their palaeontological implications. *Zool. J. Linn. Soc*. **2021b**, 193, 746–771. 10.1093/zoolinnean/zlaa166.
42. Welten, M., Smith, M., Underwood, C.J., Johanson, Z. Evolutionary origins and development of saw-teeth on the sawfish and sawshark rostrum (Elasmobranchii; Chondrichthyes). *R. Soc. Open Sci.* **2015**, 2, 2–19. 10.1098/rsos.150189.
43. Herman, J., Hovestadt–Euler, M., Hovestadt, D.C., Stehmann, M. Contributions to the study of the comparative morphology of teeth and other relevant ichthyodorulites in living supraspecific taxa of Chondrichthyan fishes. Part B: Batomorphii 4c: Order Rajiformes-Suborder Myliobatoidei-Superfamily Dasyatoidea-Family Dasyatidae-Subfamily Dasyatinae-Genus: *Urobatis*, Subfamily Potamotrygoninae-Genus: *Paratrygon*, Superfamily Plesiobatoidea-Family Plesiobatidae-Genus: *Plesiobatis*, Superfamily Myliobatoidea-Family Myliobatidae-Subfamily Myliobatinae-Genera: *Aetobatus*, *Aetomylaeus*, *Myliobatis* and *Pteromylaeus*, Subfamily Rhinopterinae–Genus: *Rhinoptera* and Subfamily Mobulinae - Genera: *Manta* and *Mobula*. Addendum 1 to 4a: erratum to Genus *Pteroplatytrygon*. *Bulletin van het Koninklijk Belgisch Instituut voor Natuurwetenschappen-Biologie* **2000**, 70, 5–67.
44. Marramà, G., Carnevale, G., Naylor, G.J., Kriwet. J. Skeletal Anatomy, Phylogenetic Relationships, and Paleoecology of the Eocene Urolophid Stingray *Arechia crassicaudata* (Blainville, 1818) from Monte Postale (Bolca Lagerstätte, Italy). *Zool. J. Linn. Soc***. 2020,** 40, e1803339. 10.1080/02724634.2020.1803339.

45. Coates, M.I., Gess, R.W., Finarelli, J.A., Criswell, K.E., Tietjen, K. A symmoriiform chondrichthyan braincase and the origin of chimaeroid fishes. *Nature* **2017**, 541, 208–211. 10.1038/nature20806.
46. Frey, L., Coates, M.I., Tietjen, K., Rücklin, M., Klug, C. A symmoriiform from the Late Devonian of Morocco demonstrates a derived jaw function in ancient chondrichthyans. *Commun. Biol*. **2019**, 3, 1–10. 10.1038/s42003-020-01394-2.
47. Maisey, J.G., Janvier, P., Pradel, A., Denton, J.S.S., Bronson, A., Miller, R., Burrow, C.J. *Doliodus* and pucapampellids: contrasting perspectives on stem chondrichthyan morphology. In *Evolution and Development of Fishes*, Johanson, Z., Underwood, C., Richter, M., Eds.; Cambridge University Press: Cambridge, UK. 2019, pp 87–109.
48. Maisey, J.G., Miller, R., Pradel, A., Denton, J.S., Bronson, A., Janvier, P. Pectoral morphology in *Doliodus*: bridging the 'acanthodian'-chondrichthyan divide. *Am. Mus. Novit.* **2017**, pp 1–15. 10.1206/3875.1
49. Zangerl, R., Case, G.R. *Cobelodus aculeatus* (Cope), an Anacanthous shark from Pennsylvanian black shales of North America. *Paleontolographica, Abt. A.* **1976**, 154, 107–157.
50. Dean, M.N., Summers, A.P., Ferry, L.A. Very low pressures drive ventilatory flow in chimaeroid fishes. *Journal of Morphology*. **2011,** 273, 461–479. doi:10.1002/jmor.11035
51. Miller, R.F., Cloutier, R., Turner, S. The oldest articulated chondrichthyan from the Early Devonian period. *Nature.* **2003**, 425, 501–504.
52. Marramà, G., Carnevale, G., Naylor, G.J., Varese, M., Giusberti, L., Kriwet, J. Anatomy, taxonomy and phylogeny of the Eocene guitarfishes from the Bolca Lagerstätten, Italy, provide new insights into the relationships of the Rhinopristiformes (Elasmobranchii: Batomorphii). *Zoological Journal of the Linnean Society*. **2020**, 192, 1090-1110.https://doi.org/10.1093/zoolinnean/zlaa125
53. Pradeep, H.D., Swapnil, S.S., Nashad, M., Venu, S., Ravi, R.K., Sumitha, G., Monalisha, D.S., Farejiya, M.K. First record and DNA Barcoding of Oman cownose ray, *Rhinoptera jayakari* Boulenger, 1895 from Andaman Sea, India. *Zoosystema* **2018**, 40, 67–74, 10.5252/zoosystema2018v40a4.
