# Supplementary material for: The Phylogeny of Rays and Skates (Chondrichthyes: Elasmobranchii) Based on Morphological Characters Revisited
Source: Diversity (Basel). Author manuscript; Available in PMC 2022 Jun 22. (PMC7612883; doi:10.3390/d14060456)
Supplement: Supplementary Information [file EMS145860-supplement-Supplementary_Information.zip › Suplementary_material_index.pdf]

SEM.1 Index for the supplement (File name Suplementary_material_index)
SEM.2 Full character list and specimens studied (File name diversity-1700431-Characters)
SEM.3 Phylogenetic matrix in nexus format used in the analyses in the article (File name "Phylogeny_Batomorphii_morphological_characters_revisited_DATA_MATRIX.nex").
SEM.4 Phylogenetic matrix in TNT format used in the parsimony analyses in the article (File name "TNT_Data_matrix.tnt").
SEM.5 Commands used in the Parsimony analysis, in an executable script for TNT (File name "TNT_ANALYSIS_SCRIPT.tnt").
SEM.6 Commands used to estimate the Bremer support of the groups recovered by the parsimony analysis, in an executable script for TNT (File name "TNT_BREMER_SCRIPT.tnt").
SEM.7 Command for calculating the tree metrics on TNT (File name "STATS.RUN")
SEM.8 Commands used in the Maximum Likelihood analysis, in an executable script for PAUP (File name "PAUP_ANALYSIS_SCRIPT.paup").
SEM.9 Commands used to map the characters in the ML tree, in an executable script for TNT (File name "TNT_map_Script.tnt").
SEM.10 Commands used in the Bayesian inference analysis, in an executable script for MrBayes (File name "Mr_Bayes_Script.bay").
SEM.11 Log file of the parsimony analysis (File name "TNT_ANALYSIS.log").
SEM.12 Log file of the ML analysis (File name "PAUP_ANALYSIS.log").
SEM.13 Log file of the BI analysis (File name "Mr_Bayes_ANALYSIS.log").
SEM.14 Log file of the mapping of characters for the parsimony tree (File name "TNT_map.log").
SEM.15 Log file of the mapping of characters for the ML tree (File name "PAUP_map.log").
